# Latest developments in coumarin-based anticancer agents: mechanism of action and structure–activity relationship studies

**DOI:** 10.1039/d3md00511a

**Published:** 2023-10-20

**Authors:** Manankar Koley, Jianlin Han, Vadim A. Soloshonok, Subhajit Mojumder, Ramin Javahershenas, Ata Makarem

**Affiliations:** a CSIR-Central Glass & Ceramic Research Institute Kolkata India; b College of Chemical Engineering, Nanjing Forestry University Nanjing China; c Department of Organic Chemistry I, University of the Basque Country San Sebastián Spain; d IKERBASQUE, Basque Foundation for Science Bilbao Spain; e Department of Organic Chemistry, Faculty of Chemistry, Urmia University Urmia Iran; f Institute of Pharmacy, University of Hamburg Hamburg Germany ata.makarem@uni-hamburg.de

## Abstract

Many researchers around the world are working on the development of novel anticancer drugs with different mechanisms of action. In this case, coumarin is a highly promising pharmacophore for the development of novel anticancer drugs. Besides, the hybridization of this moiety with other anticancer pharmacophores has emerged as a potent breakthrough in the treatment of cancer to decrease its side effects and increase its efficiency. This review aims to provide a comprehensive overview of the recent development of coumarin derivatives and their application as novel anticancer drugs. Herein, we highlight and describe the largest number of research works reported in this field from 2015 to August 2023, along with their mechanisms of action and structure–activity relationship studies, making this review different from the other review articles published on this topic to date.

## Abstract 2

Recent advances in coumarin-based anticancer agents.

## Graphical abstract

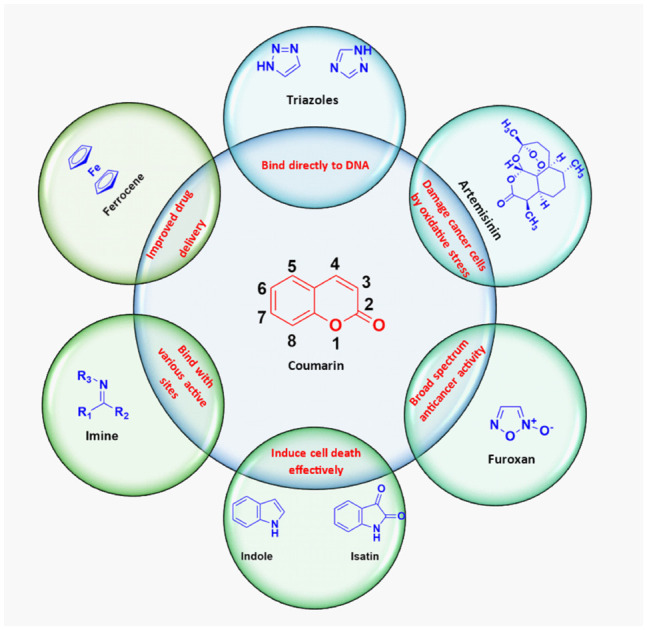

## 1. Introduction

Cancer can occur in any organ or tissue of the body, and the two properties that make cancer cells particularly dangerous are that they can abnormally divide and colonize regions normally reserved for normal cells. According to recent WHO reports,^1^ in 2020, 10 million deaths were caused by cancer. In 2020, the most common (in terms of new cases of cancer) was breast cancer with 2.26 million new cases and the most common cause of death due to cancer was lung cancer with 1.80 million deaths. Besides, according to the American Cancer Society, an estimated 1.9 million new cancer cases will be diagnosed and 609 360 deaths will occur in the United States alone.^2^ Cancers of female breasts (2.26 million), lung (2.21 million), and colon and rectum (1.93 million) were the top three cancer types in terms of incidence in 2020, while in terms of mortality, lung (1.80 million), colon and rectum (916 000), and liver (830 000) cancers topped the list. Therefore, it remains a challenge for medicinal chemists to develop new strategies for preparing novel anticancer drugs to fight this fatal disease.

**Fig. 1:**
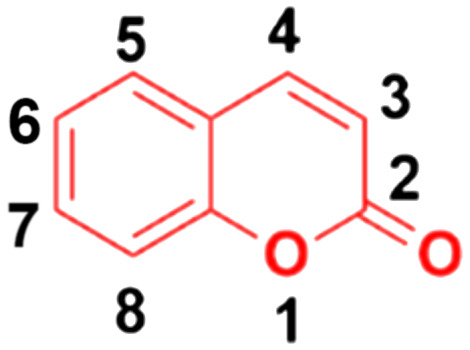
Chemical structure of coumarin.

Around 80% of the approved anticancer drugs are natural products.^3^ Coumarin (2*H*-1-benzopyran-2-one, Fig. 1) and its derivatives possess a wide variety of pharmacological properties, such as anti-inflammatory,^4,5^ antibacterial,^6–9^ antiviral,^10^ antioxidant,^11–13^ antirhombotic,^14^ anti-Alzheimer,^15,16^ and anticancer^17,18^ activities. Accordingly, many researchers have thoroughly investigated the various mechanisms of action of different classes of coumarin-based anticancer agents, such as alkylating agents, angiogenesis inhibitors, kinase inhibitors, topoisomerase inhibitors, telomerase inhibitors, antimitotic activity, human carbonic anhydrase inhibitors, aromatase inhibitors, monocarboxylate transporter inhibitors, and hormonal antagonists.

In the search for novel anticancer drugs, natural products are always a major source. Many coumarin derivatives derived from natural sources show potential *in vitro* and *in vivo* anticancer activity.^19–22^ Moreover, the concept of molecular hybridization, where two or more potential pharmacophores are combined into a single molecular framework, may provide fruitful results in the treatment with the coumarin moiety given that it may lead to the generation of new anticancer drugs with low toxicity, improved specificity, and enhanced effectivity.^23^

In this review, we aim to provide an overview of the recent developments of coumarin-derived hybrids as potentially important anticancer drugs, focusing on their structure–activity relationship and mechanisms of action and highlighting articles published between 2015 and August 2023. In contrast to other review articles published on this topic, our article presents the greatest number of examples reported in this field during the past 8 years. Although the recently published reviews presented an interesting overview regarding the progress of coumarin-based anticancer agents in the past,^24,25^ they only discussed a selected number of coumarin hybrids, not describing this topic as extensively as done herein. However, in this context, recently Cardona-Galeano and co-workers provided an interesting bibliometric analysis of coumarin hybrids.^26^ In another review article, Al-Warhi *et al.* discussed a group of coumarin anticancer hybrids based on their mechanism of action.^27^

## 2. Coumarin hybrids

### 2.1. Coumarin-artemisinin hybrids

**Fig. 2:**
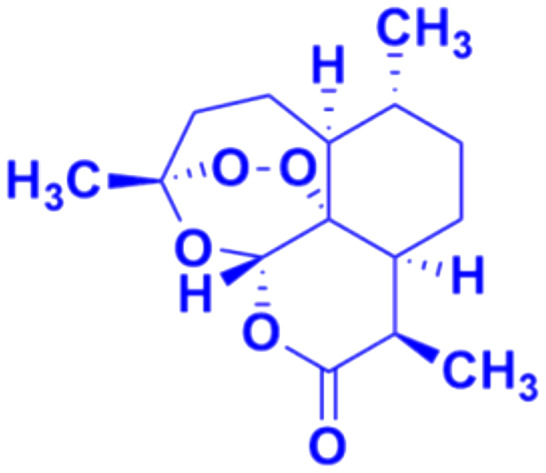
Chemical structure of artemisinin.

Artemisinin (Fig. 2), having an endoperoxide-bridged sesquiterpene lactone architecture, was discovered in 1972 by Tu Youyou. It is extracted from the plant *Artemisia annua*, a herb used in traditional Chinese medicine and has been widely used in the treatment of malaria, which is caused by to *Plasmodium falciparum*. Furthermore, artemisinin also exhibits potent activity against cancer *in vivo*.^28,29^ The activity of artemisinin and its derivatives is attributed to the presence of the endoperoxide 1,2,4-trioxane ring, which can cause oxidative stress and damage to cancer cells.^30^ Hence, the hybridization of coumarin with artemisinin is a useful strategy to design anticancer drugs with enhanced effectiveness.

**Fig. 3:**
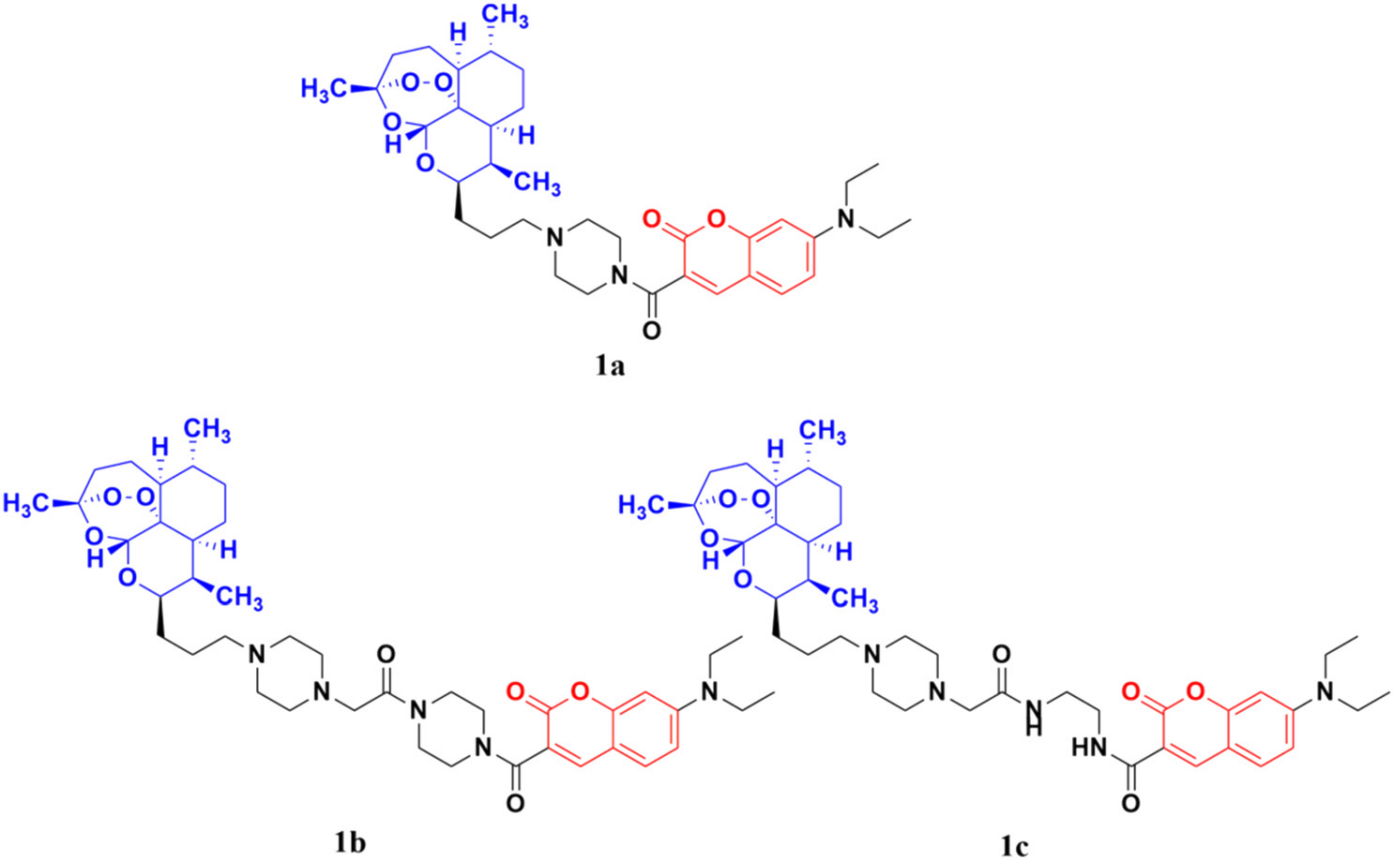
Chemical structures of coumarin-artemisinin hybrid 1a–c.

The coumarin-artemisinin hybrid 1a (Fig. 3) showed potential activities against four cancer cell lines, *i.e.*, HepG2 (IC_50_ = 3.05 ± 1.60 μM), Hep3B (IC_50_ = 3.76 ± 1.76 μM), A2780 (IC_50_ = 5.82 ± 2.28 μM), and OVCAR-3 (IC_50_ = 4.60 ± 1.81 μM). It was also reported that hybrid 1a is more potent than hybrids 1b and c, which has a linker between the piperazinyl and carbonyl groups. Fluorescence images revealed that the hybrids localized mainly in the mitochondria and their enhanced potency is due to their strong ability to accumulate in the mitochondria, which enhances the intracellular reactive oxygen species (ROS) level and triggers cell death.^31^

**Fig. 4:**
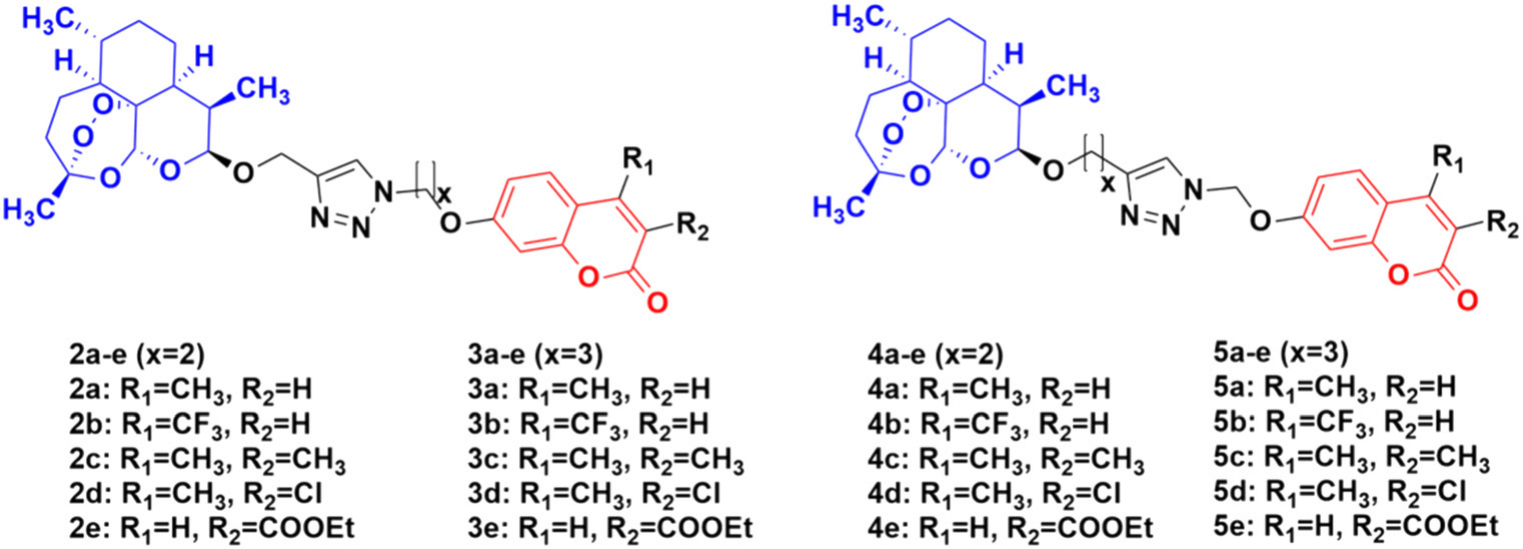
Chemical structures of coumarin-artemisinin hybrids 2a–e, 3a–e, 4a–e and 5a–e.

Four series of twenty coumarin-artemisinin hybrids were developed using click chemistry, which showed efficient activity (IC_50_ = 0.05–125.40 μM) when assessed (MTT assay) under normoxic or anoxic conditions against three cancer cell lines (HCT-116, MDA-MB-231, and HT-29).^32,33^ It is worth noting that click chemistry has a wide application in medicinal chemistry.^34–37^ The structure–activity relationship (SAR) studies showed that the 3-chloro and 4-methyl substituents in the coumarin moiety exhibited greater activity, whereas the 3-ethoxycarbonyl group in the coumarin ring exhibited reduced effectivity. It was also found that all these hybrids exhibited greater activity against the HT-29 cancer cell line under anoxic conditions. The first series of compounds (2a–e) (Fig. 4) exhibited IC_50_ values in the range of 0.05–91.21 μM, while the hybrids in the second (3a–e), third (4a–e), and fourth (5a–e) series showed IC_50_ values in the range of 1.22–120.72 μM, 2.46–125.40 μM, and 0.43 > 100 μM, respectively. It was examined that the cytotoxic activities of most of these targeted compounds are 1–10-fold greater under anoxic conditions than that under normoxic conditions.

**Fig. 5:**
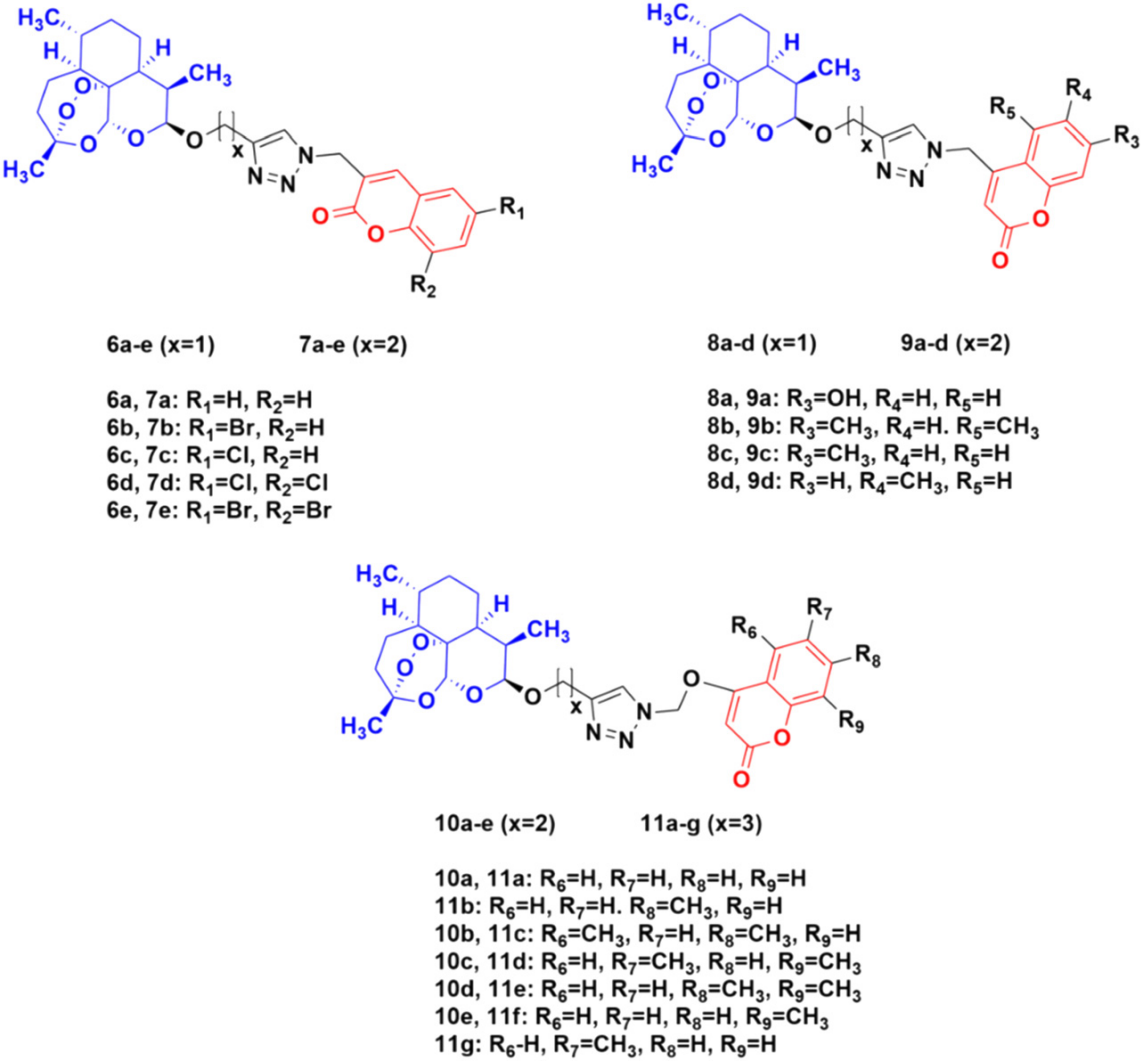
Chemical structures of coumarin-artemisinin hybrid 6a–e, 7a–e, 8a–d, 9a–d, 10a–e and 11a–g.

The potential activity of the hybrids when the linker is moved from the C-7 position to the C-4 position of the coumarin moiety was also examined and compared to doxorubicin (DOX) and DHA.^38^ A total of thirty novel hybrids were designed and developed and their cytotoxicity against four cancer cell lines (HCT-116, MDA-MB- 231, HT-29, and A-549) was investigated. It was proven that the series of compounds 10 (IC_50_ = 1.282 > 100 μM) and 11 (IC_50_ = 0.039–93.53 μM) showed a better cytotoxicity effect than the others, indicating that the 4-oxygen group in the coumarin ring as a part of the linker can enhance the potency (Fig. 5). The compounds in series 8 (8.57 > 100 μM) and 9 (9.33 > 100 μM) also showed significant activity against the HT-29 cancer cell line. In contrast, the cytotoxicity effect of compounds in series 6 and 7 was not very promising.

These hybrids inhibited the proliferation of HT-29 cells, arrested their G0/G1 phase, reduced the migration of tumor cells, and induced both apoptosis and ferroptosis in the HT-29 cancer cell line.

### 2.2. Coumarin-azole hybrids

**Fig. 6:**
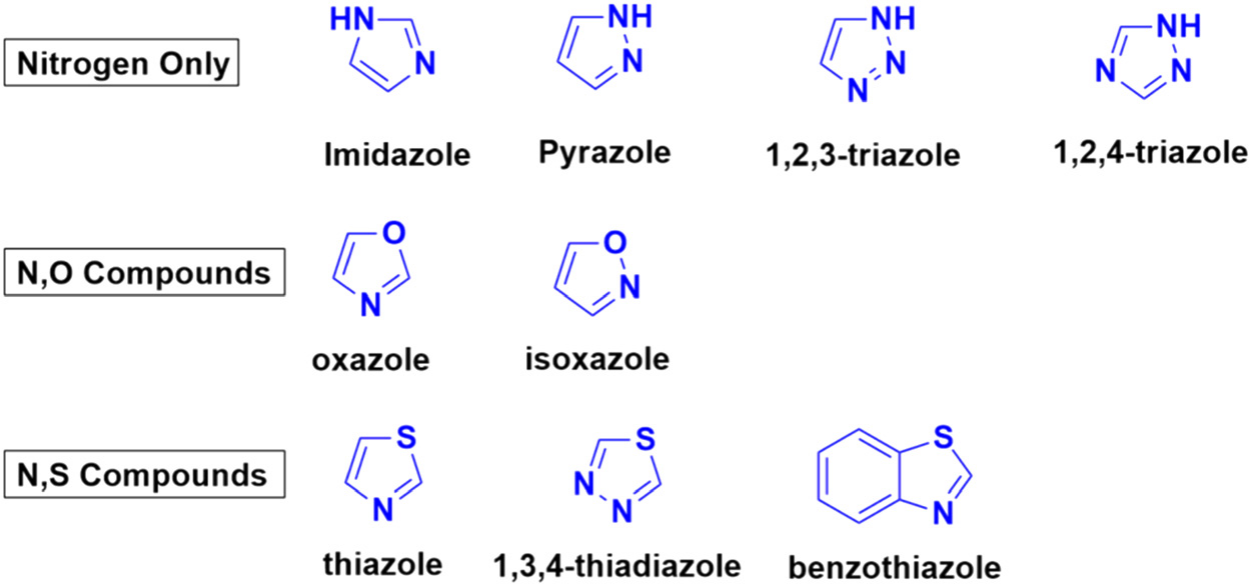
Chemical structures of some azoles possessing biological importance.

Azoles are considered one of the most important heterocycles, consisting of a five-membered ring containing one nitrogen atom and at least one other non-carbon atom (*i.e.*, nitrogen, oxygen, and sulfur). Different types of azoles (Fig. 6) possess significant biological effects^39^ and they have been fused with the coumarin moiety to develop novel anticancer drugs with increased anticancer properties.

#### 2.2.1. Coumarin–triazole hybrids

Triazoles such as 1,2,3-triazole have been shown to be a very common pharmacophore in various antitubercular, antifungal, anti-bacterial, and antitumor drugs.^40^ Their activities are due to their various non-covalent interactions, which enhance their solubility and binding ability. Besides 1,2,3-triazole, 1,2,4-triazole also possesses various pharmacological properties and this moiety can positively affect various parameters of a particular drug to increase its efficiency.^41^ Thus, these two triazoles have emerged as common choices for hybridization by medicinal chemists together with the coumarin moiety to design more potent anticancer agents.

**Fig. 7:**
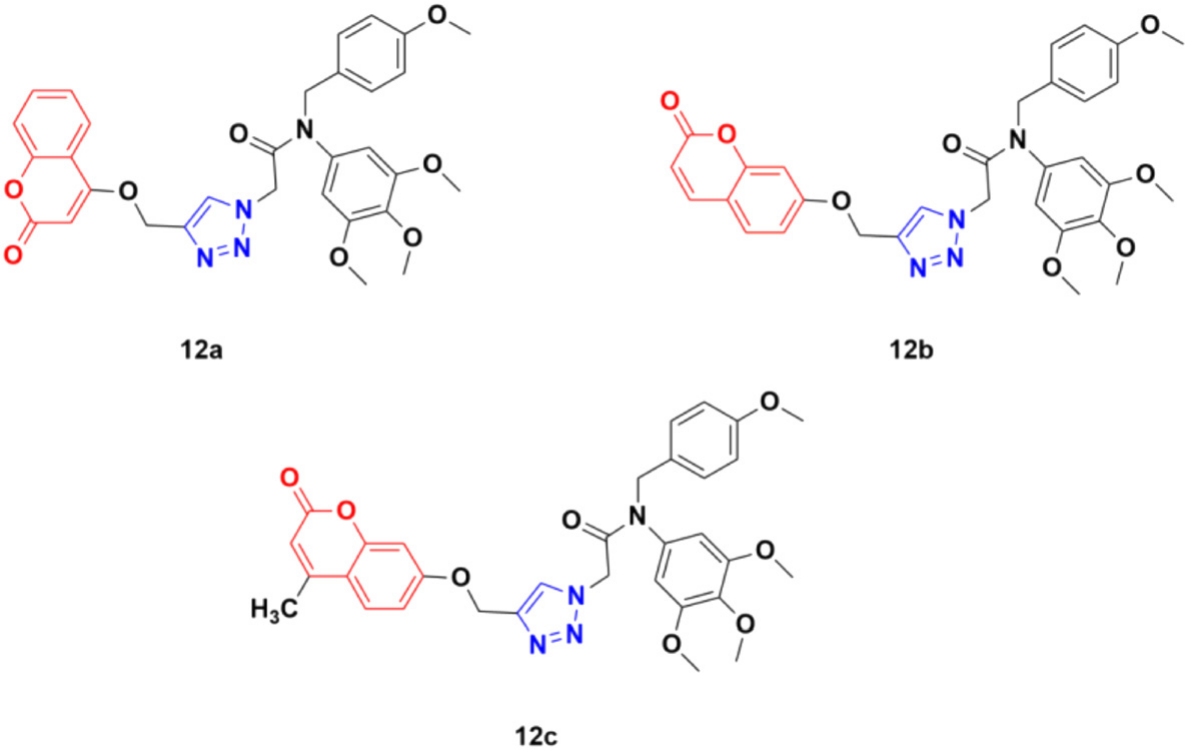
Chemical structures of coumarin–1,2,3-triazole hybrid 12a–c.

Three coumarin–1,2,3-triazole hybrids (12a–c) (Fig. 7) were synthesized and examined for their potent activity against three cancer cell lines (PC3, MGC803, and HepG2).^42^ Among them, 12c was found to be the most effective with IC_50_ values of 0.34 ± 0.04 μM, 0.13 ± 0.01 μM, and 1.74 ± 0.54 μM against PC3, MGC803, and HepG2 cancer cell lines, respectively. This hybrid was found to inhibit MGC803 cell growth, induce G2/M phase arrest and apoptosis, and regulate the expression of apoptosis-related proteins.

**Fig. 8:**
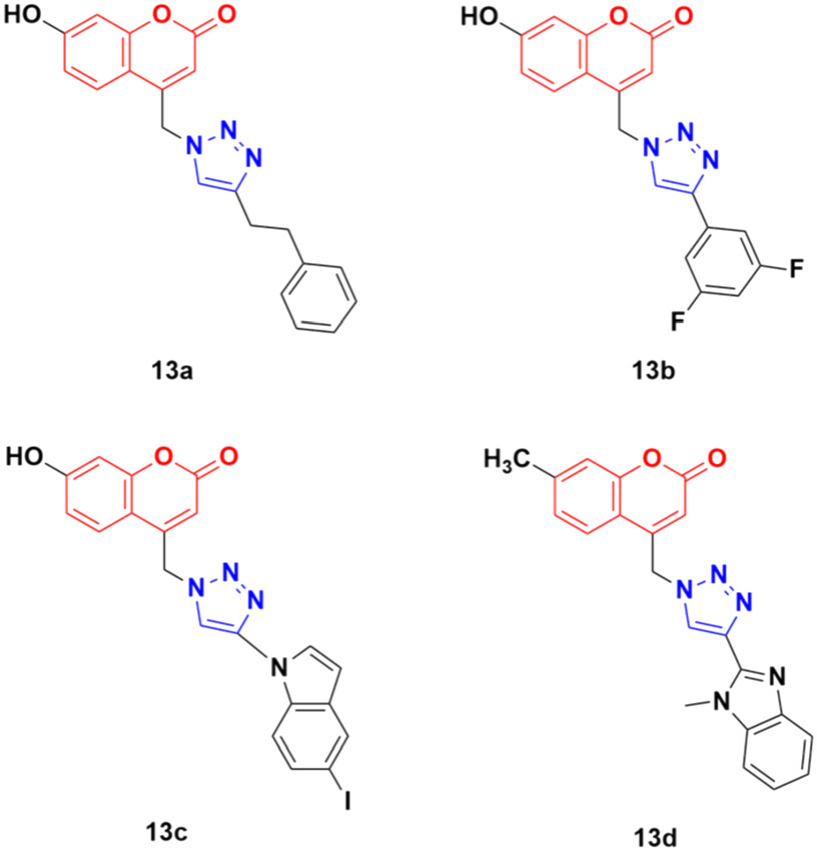
Chemical structures of coumarin–1,2,3-triazole hybrid 13a–d.

Thirty-two novel coumarin–1,2,3-triazole hybrids (Fig. 8) were designed and synthesized *via* the eco-friendly copper(i) catalyzed Huisgen 1,3-dipolar cycloaddition under microwave irradiation and their potency was evaluated against five cancer cell lines (A549, HepG2, CFPAC-1, HeLa, and SW620).^43^ Among them, the hybrids containing phenylethyl (13a) and 3,5-difluorophenyl (13b) showed the maximum potent activity against the A549 cell line (IC_50_ = 24.78 μM and 21.06 μM, respectively). Also, 13c containing 5-iodoindole exhibited significant potency against the HepG2 cancer cell line with an IC_50_ value of 8.57 μM. Compound 13d was highlighted as a lead with the highest cytotoxicity against the HepG2 cell line and an IC_50_ value of 0.90 μM. This antiproliferative activity of 13d is due to the suppression of 5-lipoxygenase activity and perturbation of sphingolipid signaling by interfering with intracellular acid ceramidase activity. It induced cell death by early apoptosis.

**Fig. 9:**
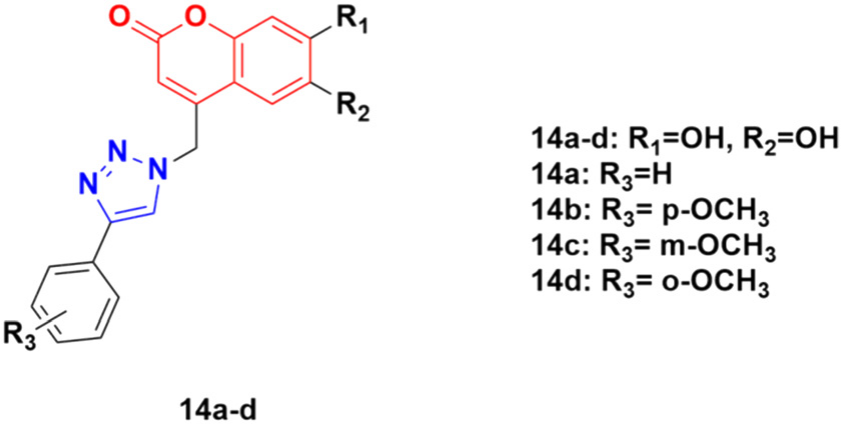
Chemical structures of coumarin–1,2,3-triazole hybrid 14a–d.

A series of other coumarin–1,2,3-triazole hybrids was developed *via* a similar procedure to that previously mentioned and their antiproliferative activities against three cancer cell lines (HeLa, CaCo-2, and K562) and normal kidney MDCK1 cells were investigated.^44^ Among them, compounds 14a–d (Fig. 9) showed the most pronounced activity, although they were also found to be cytotoxic against normal MDCK1 cells. 14c possessed high activity against the K562 cell line with an IC_50_ of value 17.9 ± 5.0 μM, whereas 14d showed significant activity against CaCo-2 cell lines with IC_50_ value 9.7 ± 1.3 μM.

**Fig. 10:**
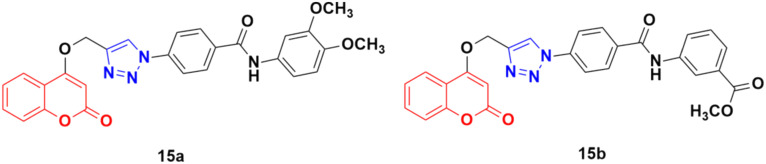
Chemical structures of coumarin–1,2,3-triazole hybrid 15a and b.

Fifteen amide-containing coumarin–1,2,3-triazole hybrids were synthesized and tested *in vitro* for their anticancer activity against the MDA-MB-231 cancer cell line under both normoxic and hypoxic conditions.^45^ Among them, compound 15a (Fig. 10) showed the maximum effectivity against the MDA-MB-231 cell line both under hypoxia (IC_50_ = 0.03 μM) and normoxia (IC_50_ = 1.34 μM), and it was proven to be more potent than doxorubicin (IC_50_ = 0.60 μM under hypoxia and IC_50_ = 1.07 μM under normoxia), *cis*-platin (IC_50_ = 4.68 μM under hypoxia and IC_50_ = 7.87 μM under normoxia), and hydroxycoumarin (IC_50_ ≥ 100 μM under hypoxia and IC_50_ ≥ 100 μM under normoxia). Compound 15b also showed moderate activity under hypoxia with an IC_50_ value of 0.25 μM. Molecular docking analysis revealed that the anticancer activity of 15a is attributed to its potential to inhibit carbonic anhydrase IX.

**Fig. 11:**
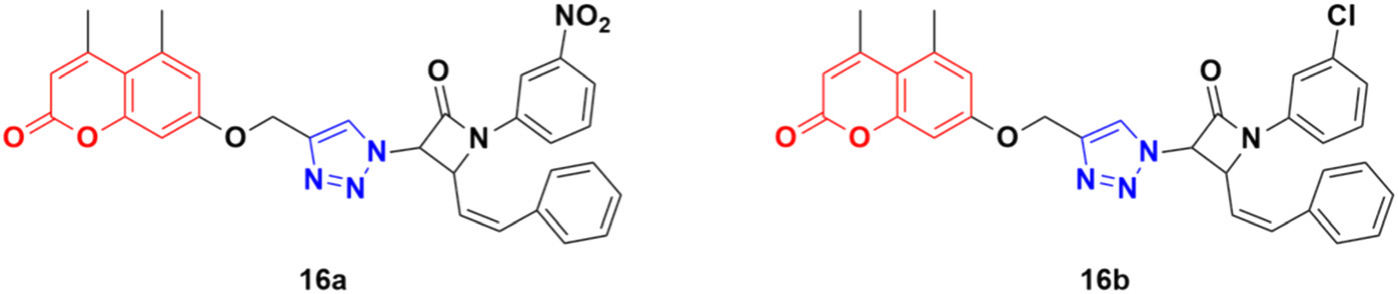
Chemical structures of coumarin–1,2,3-triazole hybrid 16a and b.

A total of fifteen coumarin-tagged β-lactam 1,2,3-triazole hybrids was designed and their anticancer activities evaluated against three cancer cell lines (A549, MCF-7, and MDA-MB-231) together with the HEK-293 normal cell line.^46^ Among them, 16a and 16b (Fig. 11) showed prominent antiproliferative activity against the MCF-7 cell line with IC_50_ = 53.55 and 58.62 μM, respectively. Molecular docking studies revealed that these two compounds target estrogen receptor-α.

**Fig. 12:**
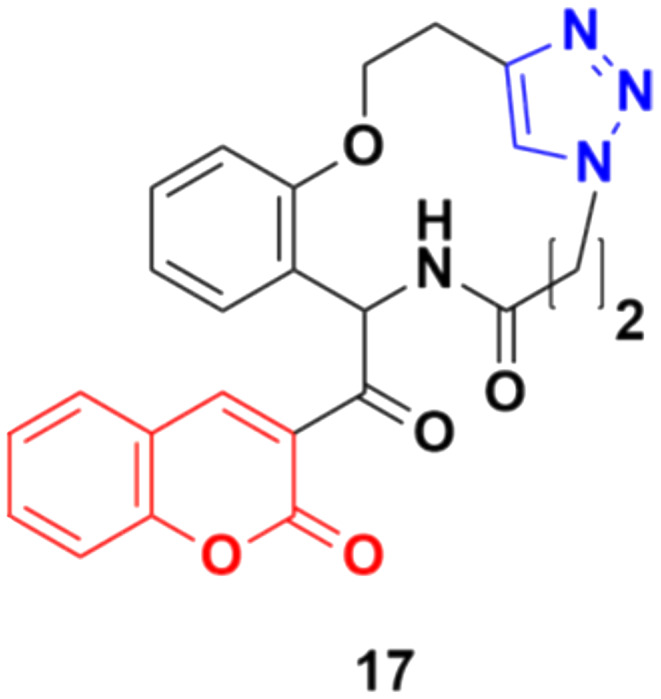
Chemical structure of coumarin–1,2,3-triazole macrocycle hybrid 17.

Some other coumarin fused 1,2,3-triazole macrocycles were also developed and found to be active against cancer cells.^47–50^ The most active hybrid 17 (Fig. 12) with IC_50_ = 49 μM only showed moderate activity against the MCF-7 cancer cell line.

**Fig. 13:**
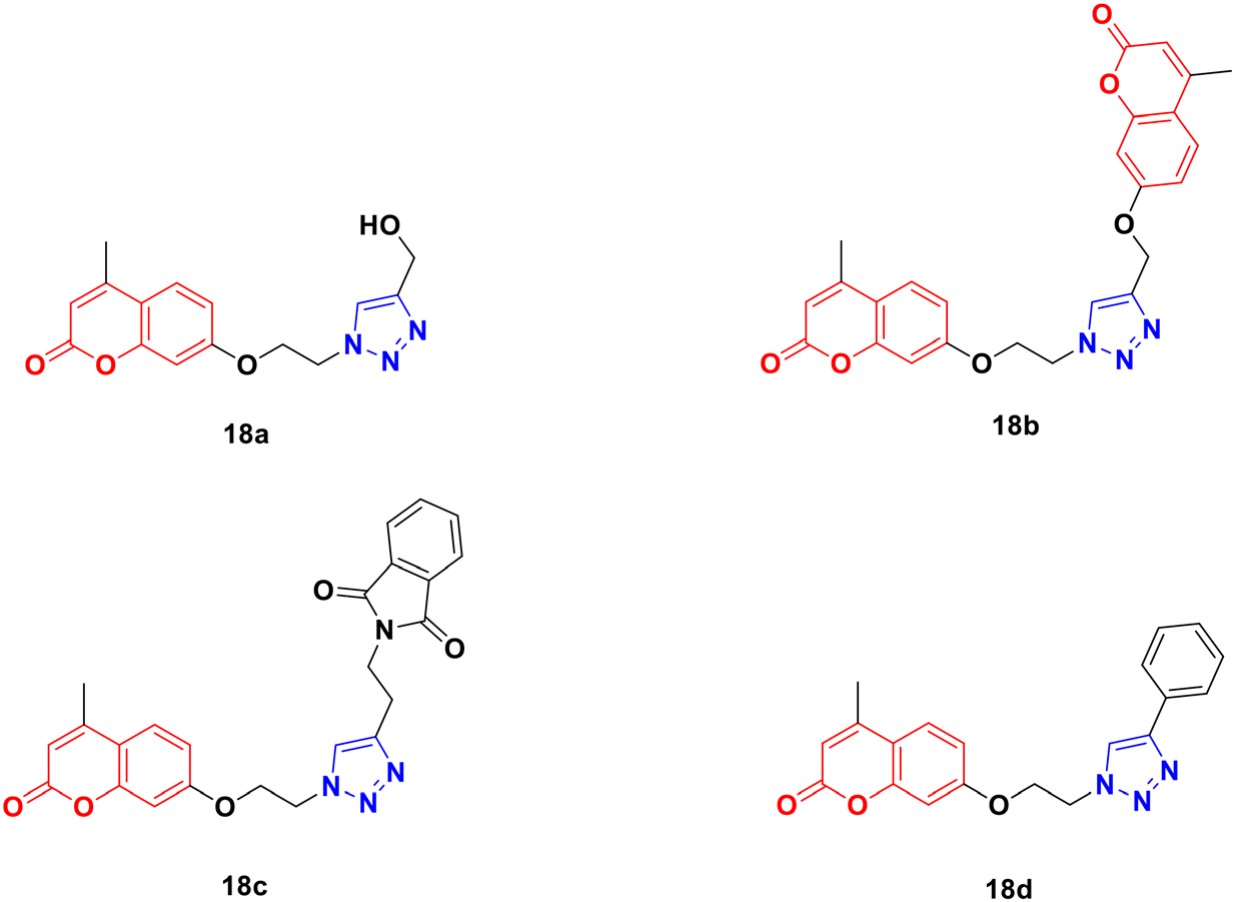
Chemical structures of coumarin–1,2,3-triazole hybrid 18a–d.

Four coumarin–triazole hybrids were chosen and examined for their cytotoxic activity on several cancer cell lines and their *in vitro* toxicity was evaluated on the 3T3 (healthy fibroblasts) cell line.^51^ They all showed significant cytotoxic activity against the MCF7 breast cancer cell line with an IC_50_ value lower than that of cisplatin, while 18c (Fig. 13) was the best among them with IC_50_ = 2.66 μM.

**Fig. 14:**
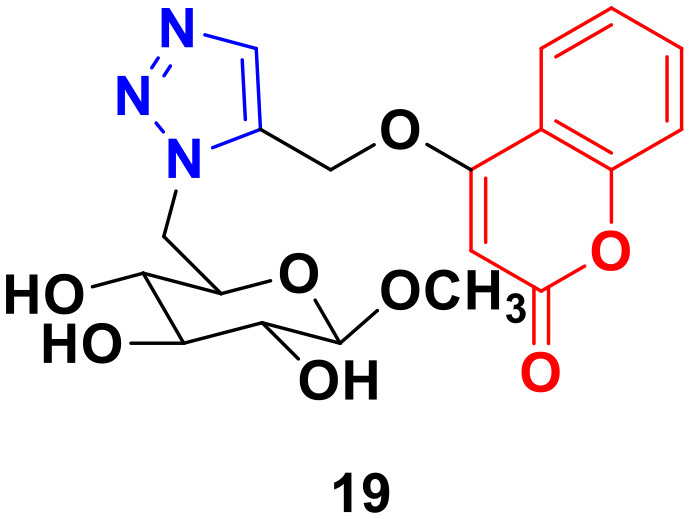
Chemical structure of coumarin–1,2,3-triazole hybrid 19.

A 1,2,3-triazole-tagged glycoside of 4-hydroxy coumarin base (19) (Fig. 14) was synthesized utilizing click chemistry and its cytotoxicity was tested against liver cancer cell lines.^52^ The IC_50_ value was found to be 106.81 μg mL^−1^.

1,2,4-Triazole has been evaluated as a novel anticancer,^53,54^ antifungal,^55^ and antibacterial^56^ activities. This nucleus is stable to metabolic degradation and is target-specific with a broad spectrum of pharmacological activities. Furthermore, given that it is polar, it can enhance the solubility of the ligand and improve its activity. Thus, this system is a likely choice for the preparation of novel anticancer agents.

**Fig. 15:**
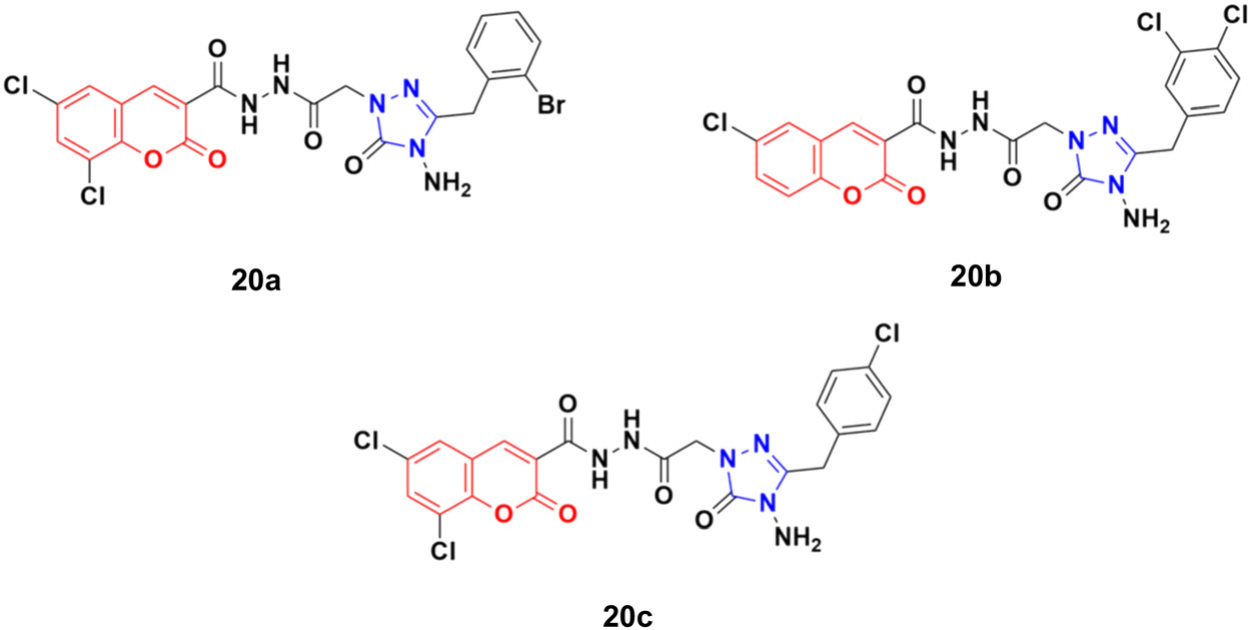
Chemical structures of coumarin–1,2,4-triazole hybrid 20a–c.

Eighteen coumarin–1,2,4-triazole hybrids were synthesized under microwave irradiation and conventional heating techniques.^57^ The compounds were investigated for their anticancer activities against four cancer cell lines (BT20 human breast carcinoma, SK-Me1 128 melanoma, DU-145 prostate carcinoma, and A549 lung carcinoma) and HFC normal cell line together with the evaluation of the selectivity index (SI). Among the hybrids, 20a (Fig. 15) showed the highest potency against the BT20 cell line with an IC_50_ value of 6.4 μg mL^−1^ and SI = 5.2. 20b was proven to be the most effective against the DU-145 (IC_50_ = 3.7 μg mL^−1^ and SI = 9.9) and SK-Me1 128 cell lines (IC_50_ = 12.3 μg mL^−1^ and SI = 3.0). 20c was the most effective against the A549 cancer cell line (IC_50_ = 7.5 μg mL^−1^ and SI = 4.2). The hybrids showed comparable activity to the reference *cis*-platin, but in general, they were not superior to the reference against the tested cancer cell lines.

#### 2.2.2. Coumarin–imidazole hybrids

**Fig. 16:**
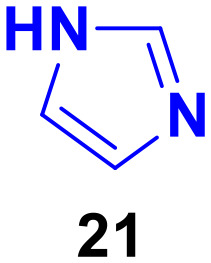
Chemical structure of imidazole.

Imidazole (21) derivatives are pharmacologically important scaffolds having anticancer, antifungal, antiprotozoal, and antihypertensive activities.^58^ Thus, coumarin-tagged imidazole hybrids can act as novel anticancer candidates and may be beneficial against both drug-sensitive and drug-resistant cancer (Fig. 16).

**Fig. 17:**
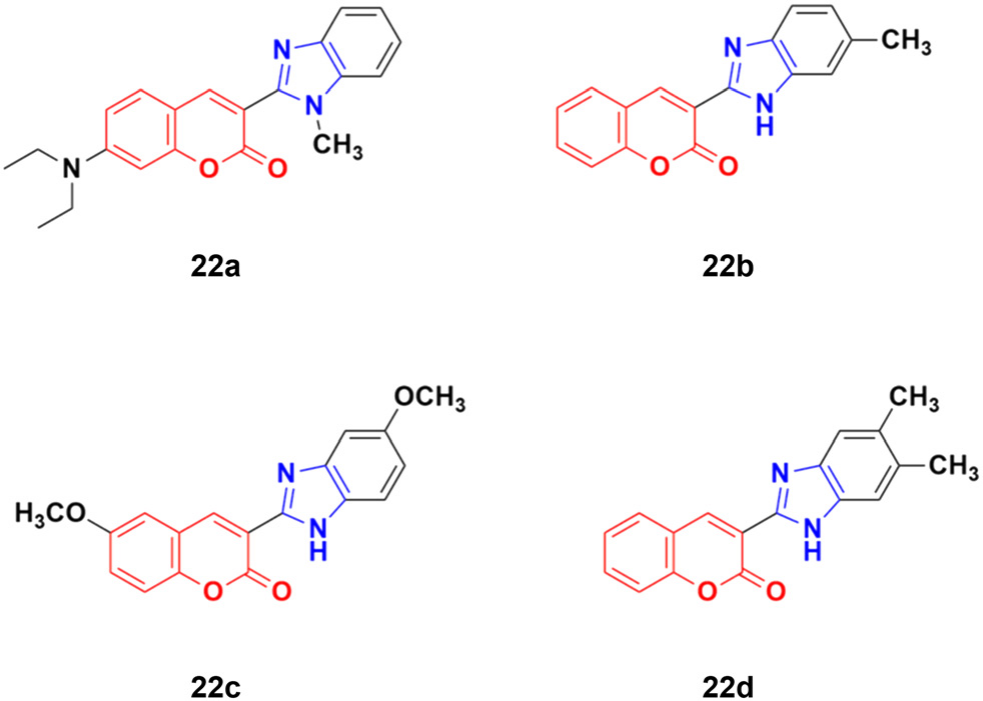
Chemical structures of coumarin–benzimidazole hybrid 22a–d.

Four coumarin–benzimidazole hybrids 22a–d (Fig. 17) were screened for their anticancer activity *via* the evaluation of their GI_50_ values against fourteen cancer cell lines, including AGS, KATO-III, SNU-1 (stomach cancer), SKOV3, OVCAR-8 (ovarian cancer), BXPC-3, PANC-1 (pancreatic cancer), T24 (bladder cancer), WiDr (colon cancer), HePG2 (liver cancer), SN12C (lung cancer), K562 (leukemia), MCF-7 (breast cancer) and HeLa.^59^ They were all fairly potent against most of the cell lines excluding MCF-7. Compound 22a possessed maximum activity against thirteen of the fourteen cell lines (GI_50_ below 0.41 μmol L^−1^). Further investigation showed that the hybrids have potent activity in inhibiting the PI3K-AKT-mTOR pathway and inducing cancer cell apoptosis.

**Fig. 18:**
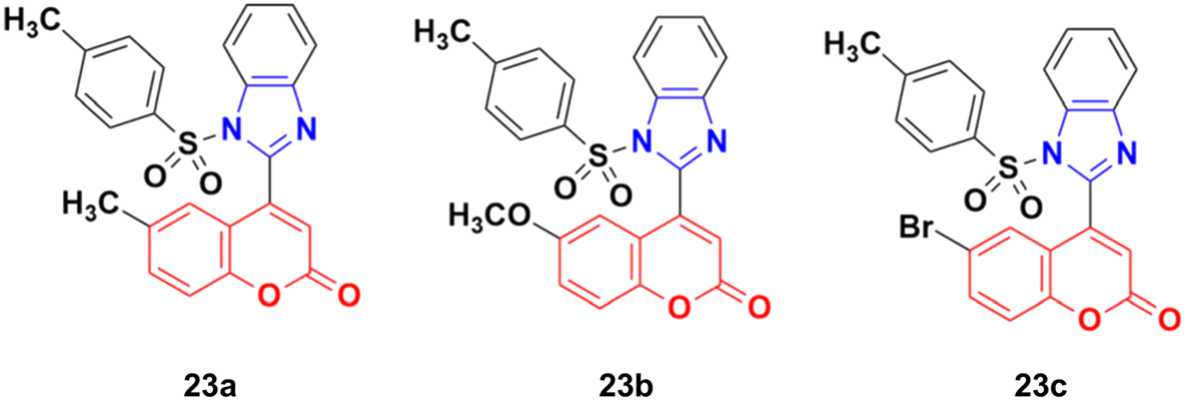
Chemical structures of coumarin–benzimidazole hybrid 23a–c.

A new series of coumarin–benzimidazole hybrids was designed and their anticancer activity was investigated in HeLa and HT29 cancer cell lines.^60^ Among them, 23a and 23b (Fig. 18) showed good potency against the HeLa cell line with GI_50_ values of 36.2 and 35.3, respectively, whereas 23a and 23c possessed marked activity against the HT 29 cell line.

**Fig. 19:**
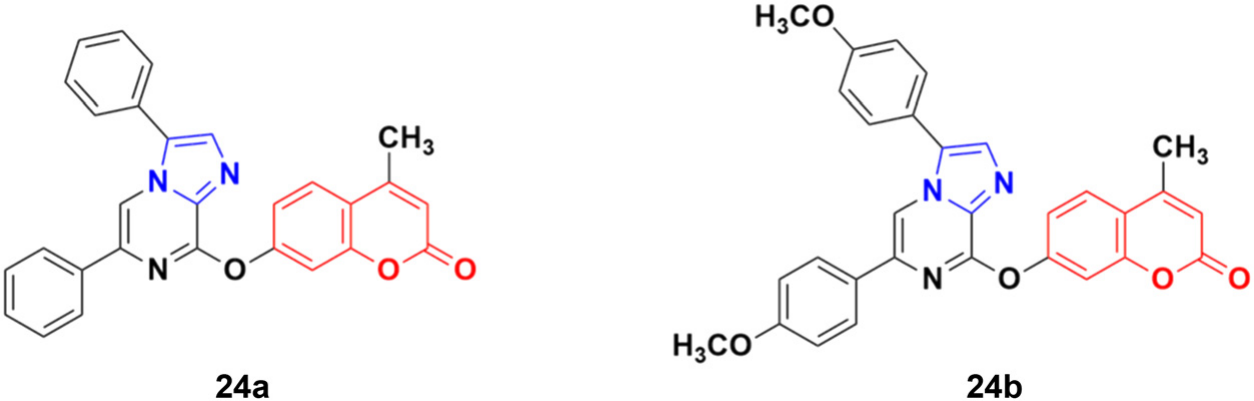
Chemical structures of imidazo[1,2-*a*]pyrazine–coumarin hybrid 24a and b.

A series of imidazo[1,2-*a*]pyrazine–coumarin hybrids was synthesized *via* the Suzuki–Miyaura coupling reaction and screened for their *in vitro* anticancer activity against sixty human cancer cell lines.^61^ Among them, compounds 24a and 24b (Fig. 19) showed a broad spectrum of activity against most of the cell lines and found to be more active than 5-fluorouracil.

**Fig. 20:**
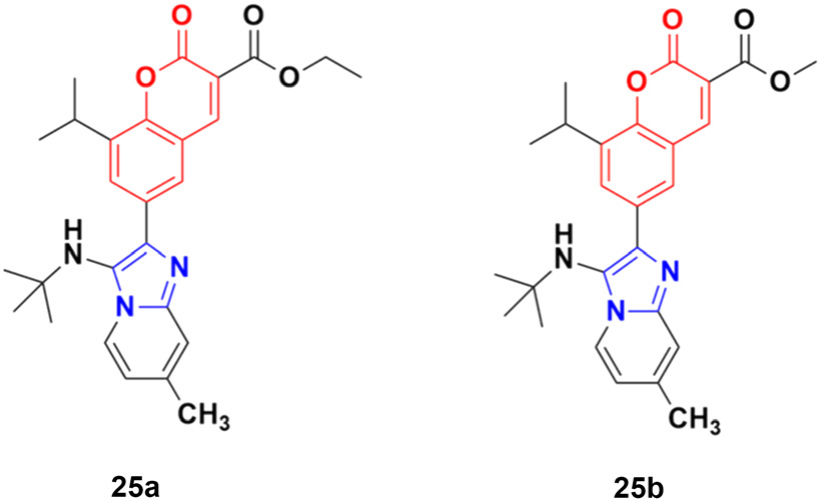
Chemical structures of imidazo[1,2-*a*]pyridine–coumarin hybrid 25a and b.

A novel series of coumarin–imidazo[1,2-*a*]pyridine derivatives was developed using silver(i)-catalyzed Groebke–Blackburn–Bienaymé multicomponent reaction and their antitumor activity was analyzed against three cancer cell lines (MCF-7, MDAMB-231, and Ishikawa).^62^ Among them, compounds 25a and 25b (Fig. 20) showed the maximum potency. Also, 25b having IC_50_ = 14.12 ± 3.69 μM against MDA-MB-231 not only induced apoptosis in cells but also induced cell cycle arrest at the G0|G1 phase in the cell. It is worth noting that we recently published some interesting review articles regarding multicomponent reactions.^63,64^

**Fig. 21:**
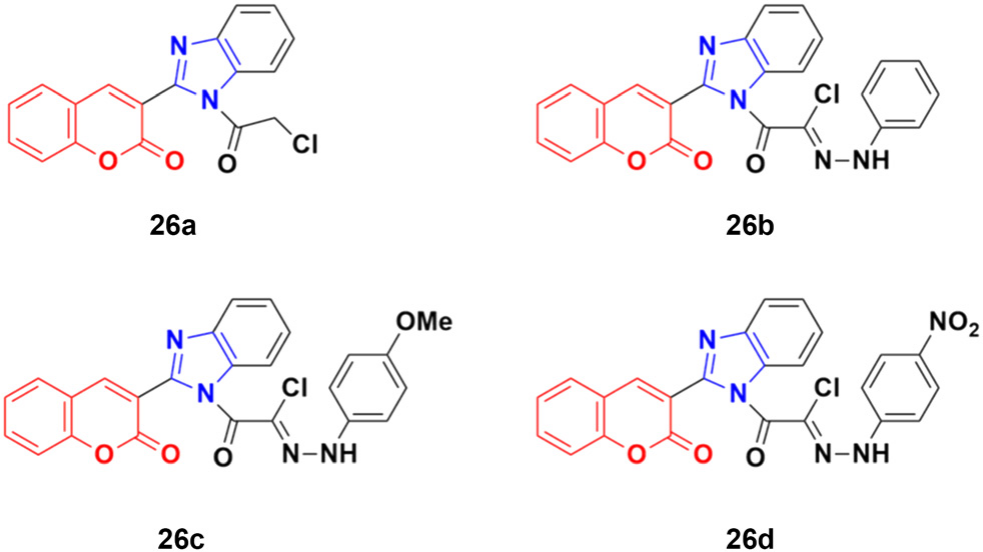
Chemical structures of coumarin–benzimidazole hybrid 26a–d.

A novel series of coumarin–benzimidazole hybrids was obtained and their growth inhibitory effect (*in vitro*) was studied against six cancer cell lines (A549, H460, HT29, MKN-45, U87MG, and SMMC-7721) using foretinib as the standard reference.^65^ The studies indicated that compounds 26a–d (Fig. 21) were the most potent against the six cancer cell lines, while compound 26a was very toxic and 26b was harmful against the tested organism. Among the non-toxic compounds, compound 26d showed the highest potency against the A549 (IC_50_ = 0.28 ± 0.04 μM) cancer cell line.

**Fig. 22:**
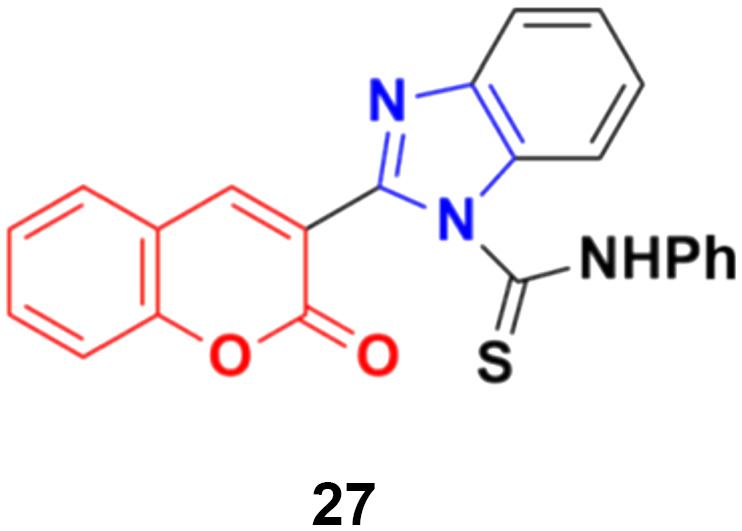
Chemical structure of coumarin–benzimidazole hybrid 27.

Another coumarin-tagged benzimidazole derivative, 2-(2-oxo-2*H*-chromen-3-yl)-*N*-phenyl-1*H-*benzo[*d*]imidazole-1-carbothioamide (27) (Fig. 22), was synthesized and its activity was investigated in three cancer cell lines (MCF-7, NCI-H460, and SF-268).^66^ This compound showed almost similar activity to the reference doxorubicin but also toxicity in normal cell lines.

**Fig. 23:**
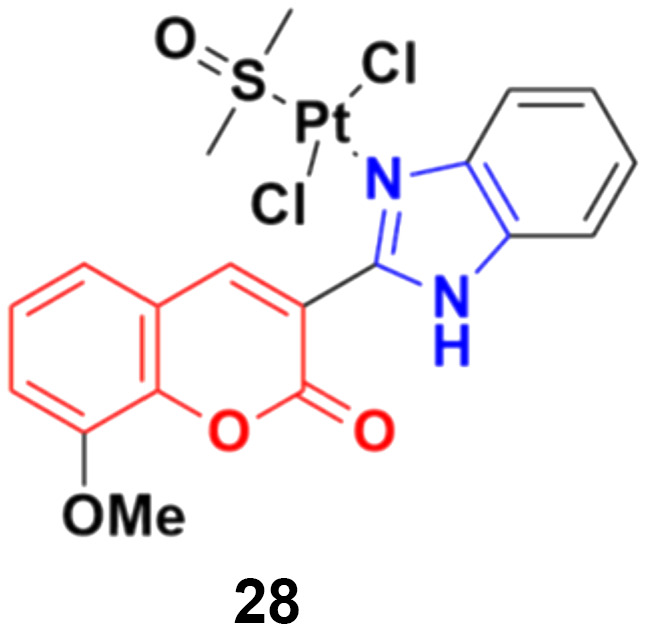
Chemical structure of platinum(ii) complex-tagged coumarin–benzimidazole hybrid 28.

Eight platinum(ii) complexes with a coumarin–benzimidazole hybrid were synthesized and their biological activity was evaluated against several cancer cell lines (HeLa, Hep-G2, and SK-OV-3/DDP).^67^ Among them, compound 28 (Fig. 23) showed excellent potency against the SK-OV-3/DDP cell line with IC_50_ = 1.01 ± 0.27 μM and was better in comparison to the reference *cis*-platin. Further investigation indicated that this compound induced apoptosis in SK-OV-3/DDP cells *via* mitochondria dysfunction signaling pathways and was a telomerase inhibitor targeting c-myc promoter elements.

**Fig. 24:**
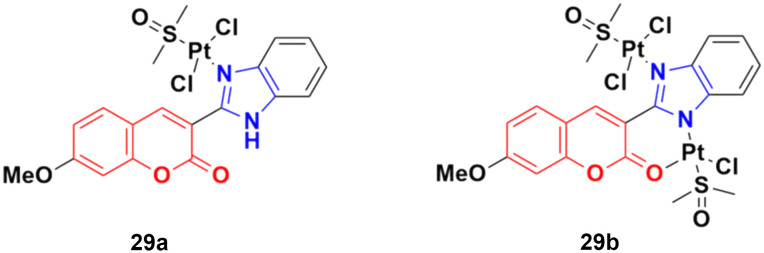
Chemical structures of platinum(ii) complex-tagged coumarin–benzimidazole hybrid 29a and b.

Two other platinum(ii) complexes, 29a and 29b (Fig. 24), with a coumarin–benzimidazole moiety also possessed excellent anticancer activity against SK-OV-3/DDP cell lines with IC_50_ values of 10.3 ± 0.3 and 0.5 ± 0.2 μM, respectively.^68^ Cytotoxic mechanism studies indicated that these two complexes act similarly to the previous platinum-based complex by inhibiting cell cycle progression at the G2/M phase and changing the expression of cell cycle-related proteins.

**Fig. 25:**
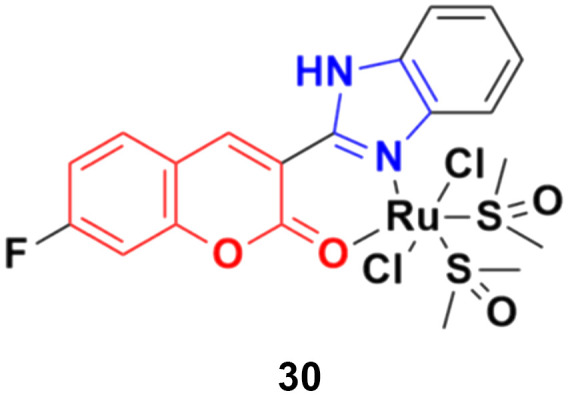
Chemical structure of ruthenium(ii) complex-tagged coumarin–benzimidazole hybrid 30.

Three ruthenium(ii) complexes with a coumarin–benzimidazole hybrid were designed and their antitumor efficiency was studied.^69^ Among them, compound 30 (Fig. 25) showed marked antitumor activity against the NCI-H460 cancer cell line (IC_50_ = 0.30 ± 0.02 μM) with high selectivity. MTT assay studies revealed that this complex induced apoptosis *via* telomerase inhibition.

**Fig. 26:**
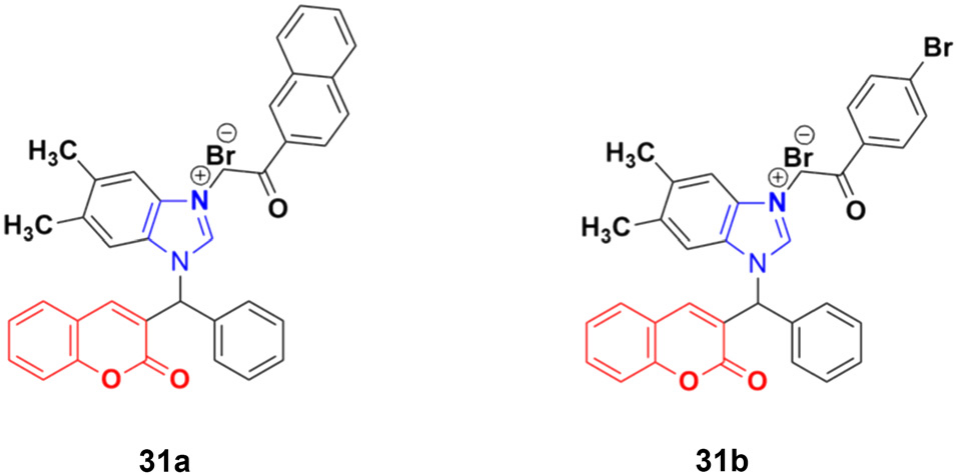
Chemical structures of 3-benzylcoumarin imidazolium salt 31a and b.

A new series of novel 3-benzylcoumarin imidazolium salts was prepared together with the evaluation of their anticancer properties against five cancer cell lines (HL-60, SMMC-7721, A-549, MCF-7, and SW-480).^70^ Among them, compound 31a (Fig. 26) showed the highest efficiency with IC_50_ values in the range of 2.04–4.51 μM against five human tumor cell lines. Compound 31b was more selective to the SW-480 cell line with an IC_50_ value 40.0-fold lower than DDP. SAR studies indicated that compound 31a can cause G0/G1 phase cell cycle arrest and apoptosis in the SMMC-7721 cell line.

**Fig. 27:**
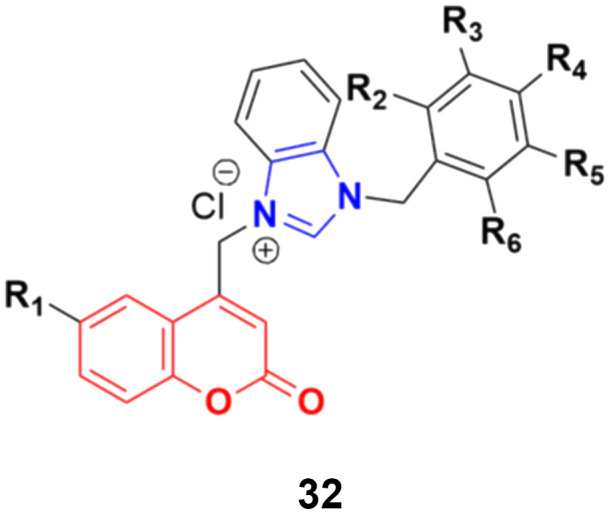
General chemical structure of a coumarin-substituted benzimidazolium salt.

A large series of coumarin-substituted benzimidazolium salts (32) (Fig. 27) was developed and their cytotoxic properties studied against PC-3 and A2780 cancer cell lines.^71^ All the salts showed moderate activity and were less active than docetaxel.

**Fig. 28:**
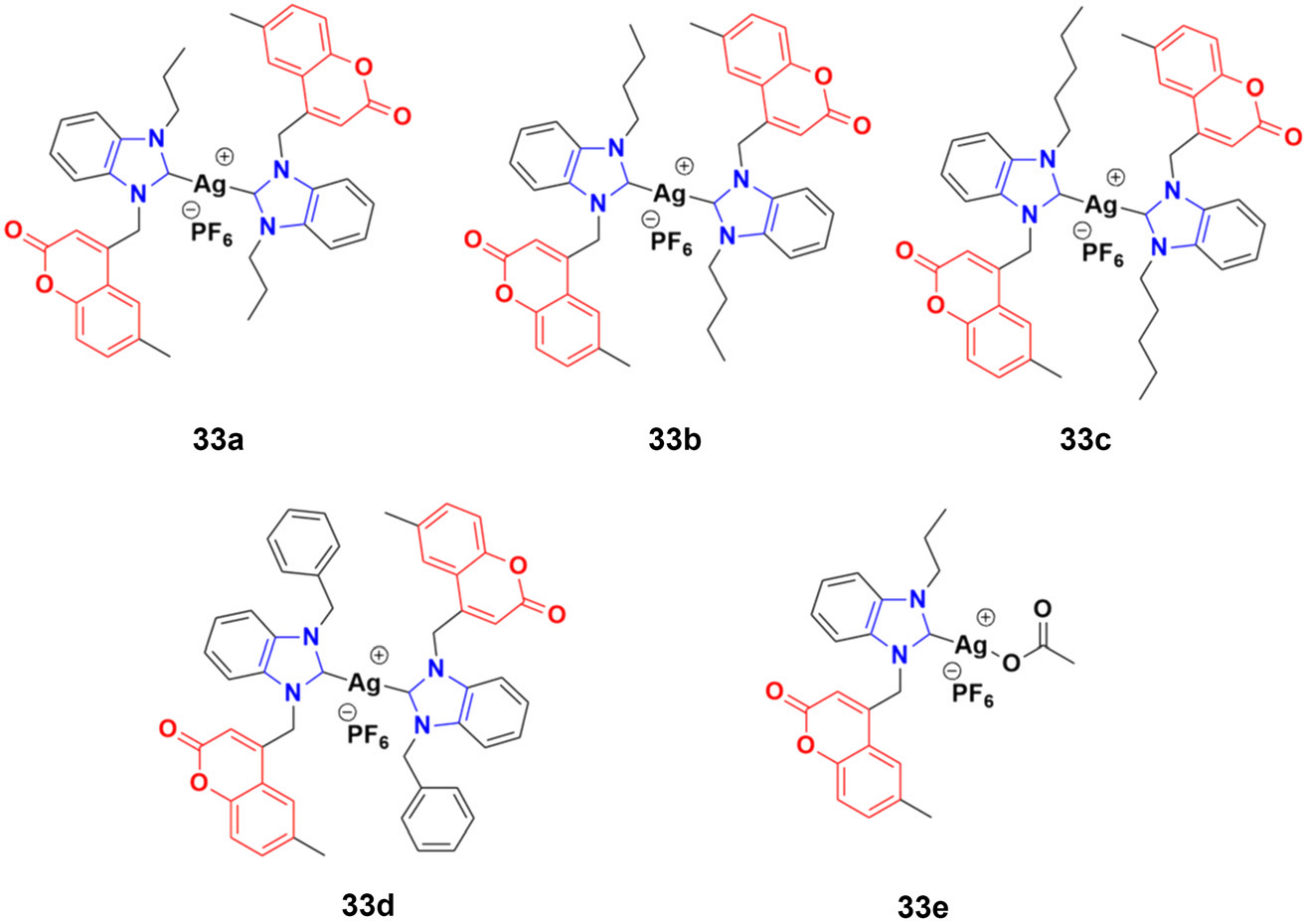
Chemical structures of silver(i)-NHC complexes with coumarin–benzimidazole hybrid 33a–e.

A novel series of sterically encumbered silver(i)-N-heterocyclic carbene complexes with coumarin–benzimidazole hybrids was designed and their cytotoxic activities studied against A549 and H1975 cancer cell lines.^72^ Complexes 33a–d (Fig. 28) showed promising activity against the above-mentioned cell lines, while complex 33e possessed a promising drug window with the IC_50_ value of 13.7 ± 2.70 and 14.5 ± 1.20 μM against the H1975 and A549 cancer cell lines, respectively.

#### 2.2.3. Coumarin–pyrazole hybrids

Pyrazole, another sophisticated heterocyclic moiety, is an important scaffold in various drugs, *e.g.*, celecoxib and anabolic steroid stanozolol. Thus, it can be a promising strategy to tag pyrazole with the coumarin moiety to design new anticancer agents with increased potential.

**Fig. 29:**
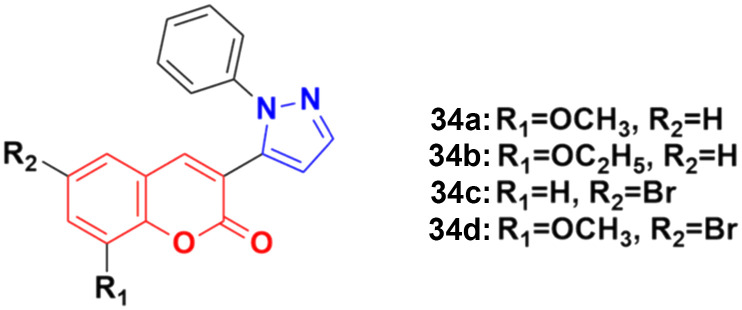
Chemical structures of coumarin–pyrazole hybrid 34a–d.

A new series of coumarin–pyrazole hybrids (34a–d) (Fig. 29) was synthesized and their activity screened against two cancer cell lines, *i.e.*, Hep-G2 and MCF-7.^73^ However, they were not very pharmacologically important given that they were all were less effective than the reference *cis*-platin and possessed moderate activity against the above-mentioned two cell lines.

**Fig. 30:**
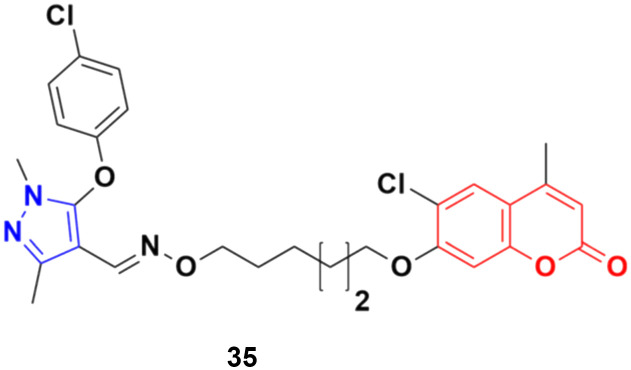
Chemical structure of coumarin–pyrazole hybrid 35.

A series of twenty-two coumarin–pyrazole hybrids was designed and their antiproliferative activities studied *in vitro* against four cancer cell lines (HepG2, SMMC-7721, U87, and H1299).^74^ Among them, compound 35 showed excellent anticancer activity against all the cell lines with IC_50_ values of 2.96 ± 0.25, 2.08 ± 0.32, 3.85 ± 0.41, and 5.36 ± 0.60 μM against the HepG2, SMMC-7721, U87, and H1299 cancer cell lines, respectively. SAR studies revealed that hybrid 35 (Fig. 30) displayed significant anti-metastasis effects by inhibiting cell migration and invasion in the highly metastatic SMMC-7721 cell line and dose-dependent reversed TGF-β1-induced epithelial-mesenchymal transition (EMT). Also, 35 showed low acute toxicity and possible tumor growth inhibitory properties against the SMMC-7721 cell line *in vivo*.

**Fig. 31:**
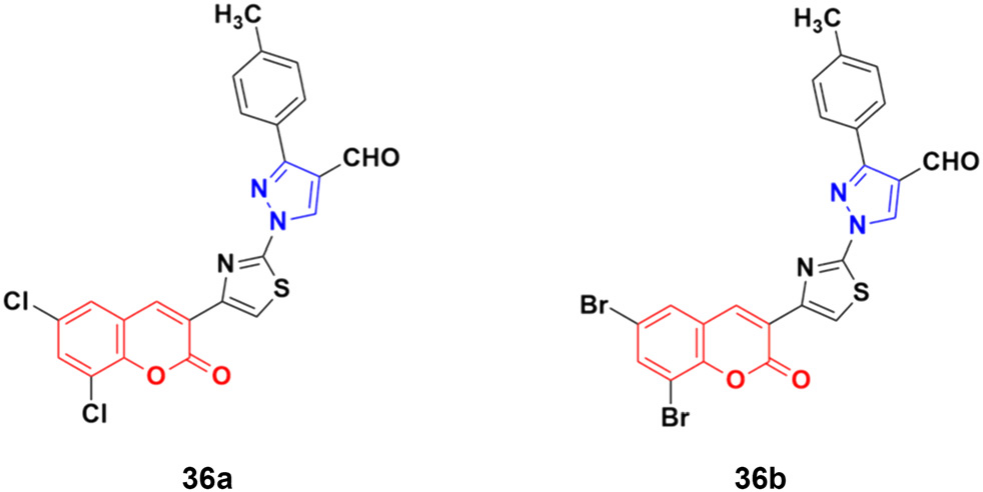
Chemical structures of coumarin–pyrazole hybrid 36a and b.

A new series of fifteen coumarin–pyrazole hybrids was synthesized *via* a one-pot multicomponent Vilsmeier–Haack reaction with good yield and their anticancer activities were screened against three cancer cell lines, *i.e.*, DU-145, MCF-7, and HeLa.^75^ All the derivatives exhibited appreciable cytotoxic activity but were not better than the reference doxorubicin. Compounds 36a and 36b (Fig. 31) showed good activity against the HeLa cell line with IC_50_ values of 5.75 and 6.25 μM, respectively.

**Fig. 32:**
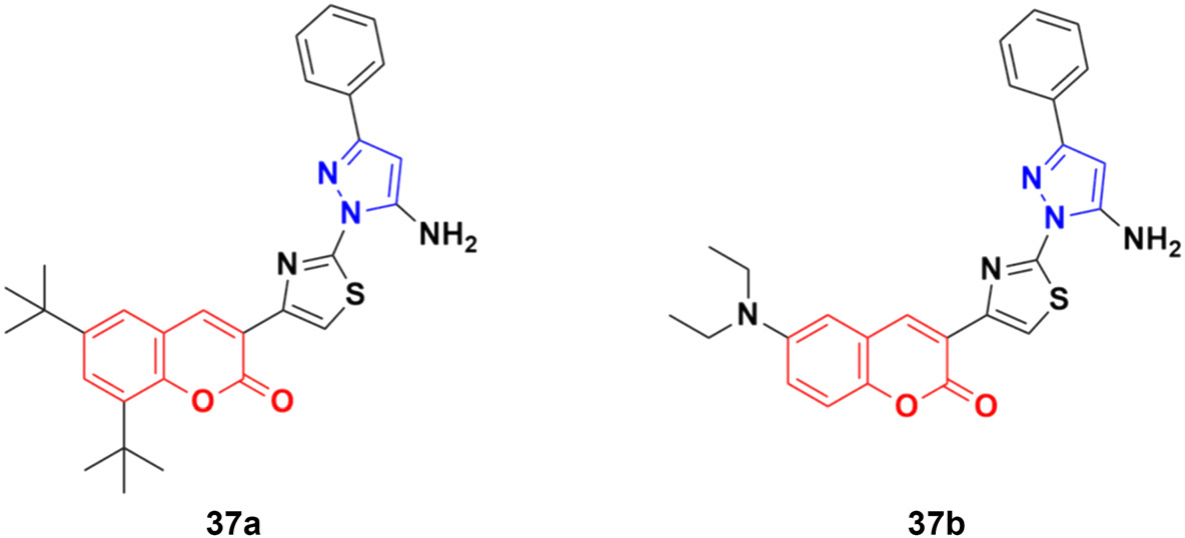
Chemical structures of coumarin–pyrazole hybrid 37a and b.

A similar series of coumarin–pyrazole hybrids was designed and their anticancer properties screened against five cancer cell lines (L1210, CEM, DU- 145, HeLa, and MCF-7).^76^ Among them, compound 37a (Fig. 32) possessed the maximum potency against the DU-145 cell line with an IC_50_ value of 7 ± 1 μM, while compound 37b showed the maximum efficiency against the MCF-7 cell line with an IC_50_ value of 8 ± 2 μM.

#### 2.2.4. Coumarin–oxa(dia)zole hybrids

Oxa(dia)zoles are privileged heterocyclic compounds and present in various biologically important compounds such as antiviral, antibacterial, antifungal, anti-inflammatory, and antibiotic compounds.

**Fig. 33:**
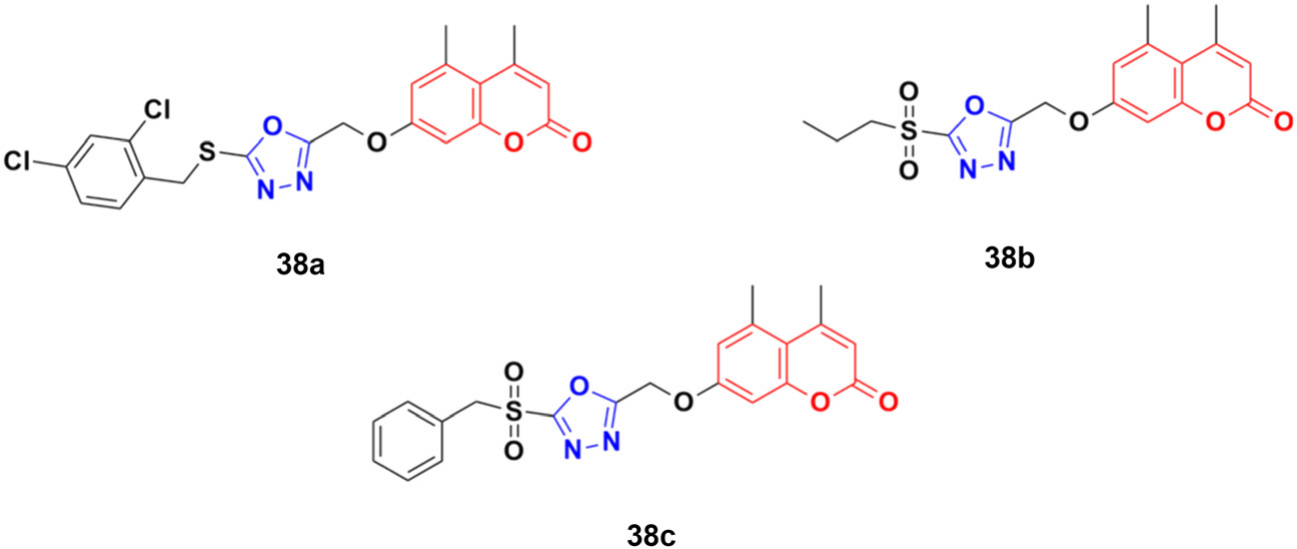
Chemical structures of coumarin–1,3,4-oxadiazole hybrid 38a–c.

A novel group of coumarin-tagged 1,3,4-oxadizaole hybrids was prepared and their biological activities studied against the MDA-MB-231 and MCF-7 breast cancer cell lines.^77^ Compound 38a (Fig. 33) showed excellent cytotoxicity against the MCF-7 cell line with an IC_50_ value of less than 5 μM, whereas compounds 38b and 38c possessed significant potency against the MDA-MB-231 cell line with an IC_50_ value of 7.07 μM for both of them. Docking studies revealed that the stronger binding affinity of the designed derivatives is due to the presence of a sulfone unit attached to the substituted benzyl group in their pharmacophores. It is worth noting that oxadiazolyl sulfones are also an emerging tool in bioconjugation methodologies.^78,79^

**Fig. 34:**
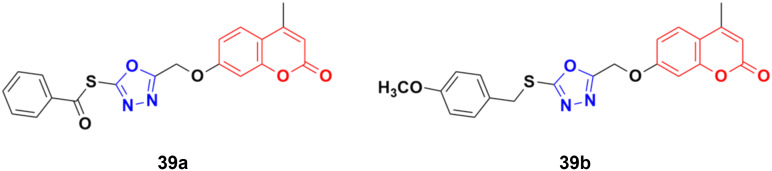
Chemical structures of coumarin–1,3,4-oxadiazole hybrid 39a and b.

A library of twenty coumarin–1,3,4-oxadiazole conjugates was synthesized and analyzed for their inhibitory activity against the four physiologically relevant human carbonic anhydrase (hCA) isoforms CA I, CA II, CA IX, and CA XII.^80^ Among them, compounds 39a and 39b (Fig. 34) exhibited significant inhibition in lower micromolar potency against hCA XII (*K*_i_ of 0.16 μM) and hCA IX (*K*_i_ of 2.34 μM), respectively. Hence, these two compounds can serve as promising leads for designing novel anticancer agents by acting through hCA IX and XII inhibition. Besides their pharmaceutical properties, thioethers and thioesters are very useful building blocks in synthetic methodologies, providing unique pathways for building new molecules.^81^

#### 2.2.5. Coumarin–thia(dia)zole hybrids

Thiazole is considered one of the most important heterocycles, which is present in various natural and synthetic compounds. Thus, it is a useful building block to prepare a new generation of potential drugs. There are numerous thiazole-based systems present that exhibit anticancer (tiazofurin), antimicrobial (sulfathiazole), antileukemia (dasatinib), immunomodulator (Fentizol), antiretroviral (ritonavir), and antifungal (ravuconazole), antiparasitic (nitazoxanide) activities.^82–85^ Hence, hybridization of the thiazole moiety with coumarin is a suitable strategy to design potential anticancer drugs.

**Fig. 35:**
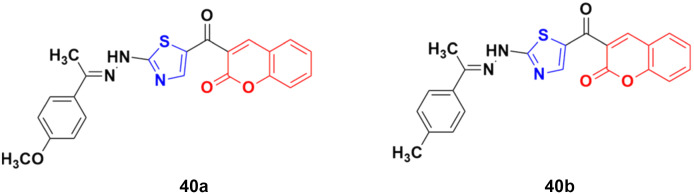
Chemical structures of coumarin–thiazole hybrid 40a and b.

A novel series of coumarin–thiazole hybrids was designed and tested by employing human colon (DLD-1) and liver cancer cell lines (HepG2).^86^ Among the nine compounds, 40a (Fig. 35) was the most effective against DLD-1 with an EC_50_ value of 5.79 μM, while compound 40b showed the maximum potency against the HepG2 cell line with an EC_50_ value of 3.70 μM. The designed compounds act by blocking Hsp90 function and were determined to be valuable C-terminal Hsp90 inhibitors.

**Fig. 36:**
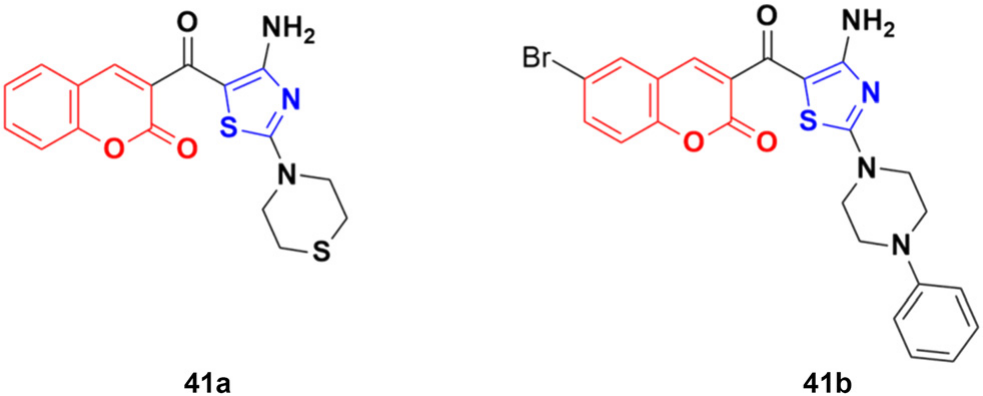
Chemical structures of coumarin–thiazole hybrid 41a and b.

A similar series of coumarin–thiazole conjugates was developed and their cytotoxic activity examined against three human cancer cell lines, *i.e.*, MCF-7, HepG2 and SW480.^87^ All the compounds showed moderate to low activity against the above-mentioned cell lines but none of them were more potent than the reference etoposide. Compound 41a (Fig. 36) showed significant efficiency (IC_50_ values of 7.5 ± 0.7, 16.9 ± 0.7, and 13.0 ± 0.6 μg mL^−1^ against MCF-7, HepG2, and SW480, respectively). Compound 41b possessed the maximum potency against the HepG2 cell line with IC_50_ = 12.2 ± 2.3 μg mL^−1^.

**Fig. 37:**
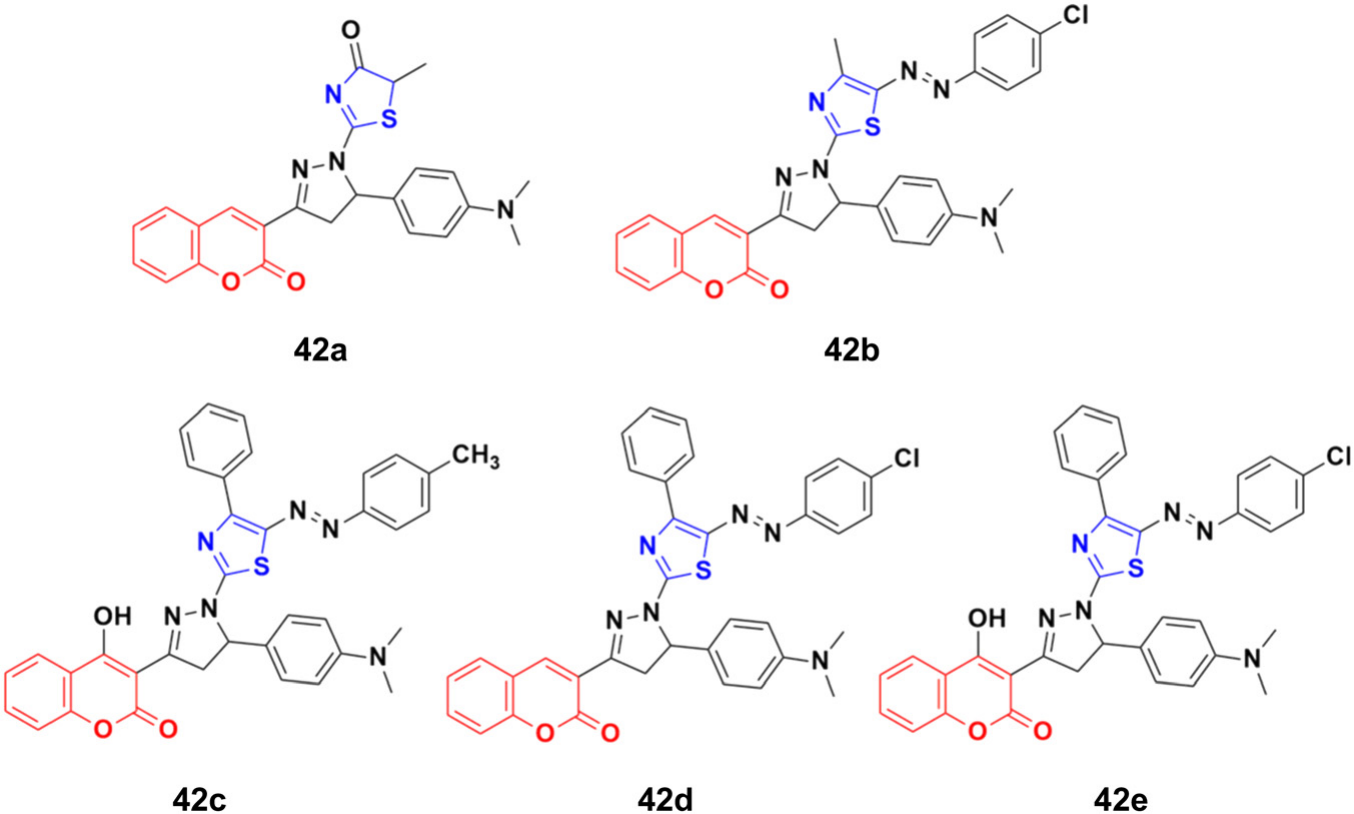
Chemical structures of coumarin–thiazole hybrid 42a–e.

A novel series of thiazolopyrazolyl coumarin derivatives was designed and their cytotoxicity examined against four cancer cell lines (MCF-7, A549, PC3, and HepG2) together with a normal cell line, HFB4.^88^ Among the synthesized hybrids, compounds 42a–e (Fig. 37) showed significant activity towards the MCF-7 cell line (IC_50_ = 5.41–10.75 μM) together with a low cytotoxic effect on the normal cell line. Several theoretical and experimental studies revealed the molecular mechanisms that control breast carcinoma metastasis. The mechanistic effectiveness in cell cycle progression, apoptotic induction, and gene regulation was analyzed for compound 42e due to its significant cytotoxicity against MCF-7 and potent VEGFR-2 inhibition. Flow cytometric analysis showed that compound 42e induced cell growth cessation at the G2/M phase and enhanced the percentage of cells in the pre-G1 phase, stimulating the apoptotic death of MCF-7 cells. Furthermore, real-time PCR assay showed that compound 42e upregulated p53 gene expression and elevated the Bax/Bcl-2 ratio. Moreover, the apoptotic induction of MCF-7 breast cancer cells was enhanced effectively through the activation of caspase-7 and 9 by compound 42e. Therefore, 42e can be considered a potent lead for the development of anti-breast cancer candidates.

**Fig. 38:**
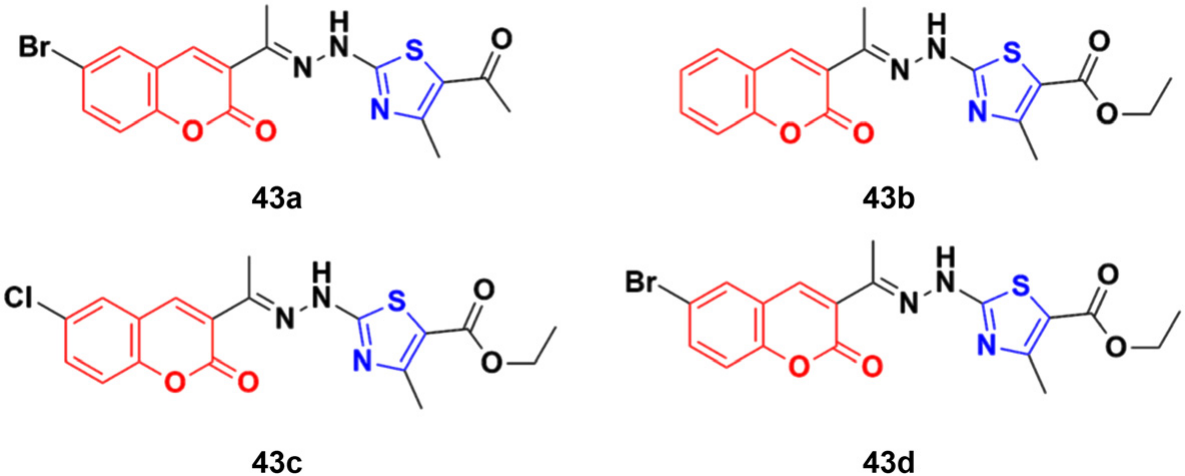
Chemical structures of coumarin–thiazole hybrid 43a–d.

A set of coumarin–thiazole hybrids 43a–d (Fig. 38) showed significant potency against HeLa cells, which was observed to be more potent than the reference doxorubicin.^89^ Compound 43d possessed maximum antiproliferative activity against the cancer cell lines with an IC_50_ value of 0.0091 ± 0.0007 cM. The cell cycle investigation showed that compound 43b led to cell cycle cessation at the G0/G1 phase, indicating that the CDK2/E1 complex can be the plausible biological target. The RT-PCR gene expression assay showed that compound 43b increased the levels of the nuclear CDK2 regulators P21 and P27 by 2.30- and 5.7-fold, respectively. The ELISA technique showed also that compound 43b led to the remarkable activation of caspase-9 and -3, inducing cell apoptosis. The molecular docking study for 43a–d rationalized their superior CDK2 inhibitory activity through their hydrogen bonding and hydrophobic interactions with the key amino acids in the CDK2 binding site.

**Fig. 39:**
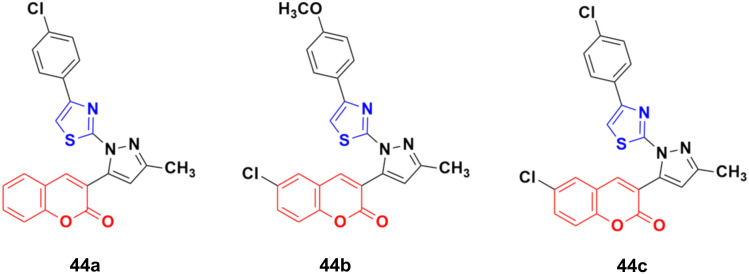
Chemical structures of 1-thiazolyl-5-coumarin-3-yl-pyrazole derivative 44a–c.

Twelve novel 1-thiazolyl-5-coumarin-3-yl-pyrazole derivatives were developed *via* a one-pot multicomponent reaction and their anticancer activity was investigated on two cancer cell lines (HepG2 and MCF7).^90^ Compound 44a (Fig. 39) showed significant cytotoxic activity against the HepG2 cell line with an IC_50_ value of 3.74 ± 0.02 μM and 44b possessed significant activity against MCF-7 with an IC_50_ value of 4.03 ± 0.02 μM. 44c was potent against both the HepG2 and MCF-7 cell lines with an IC_50_ value of 3.06 ± 0.01 μM and 4.42 ± 0.02 μM, respectively.

**Fig. 40:**
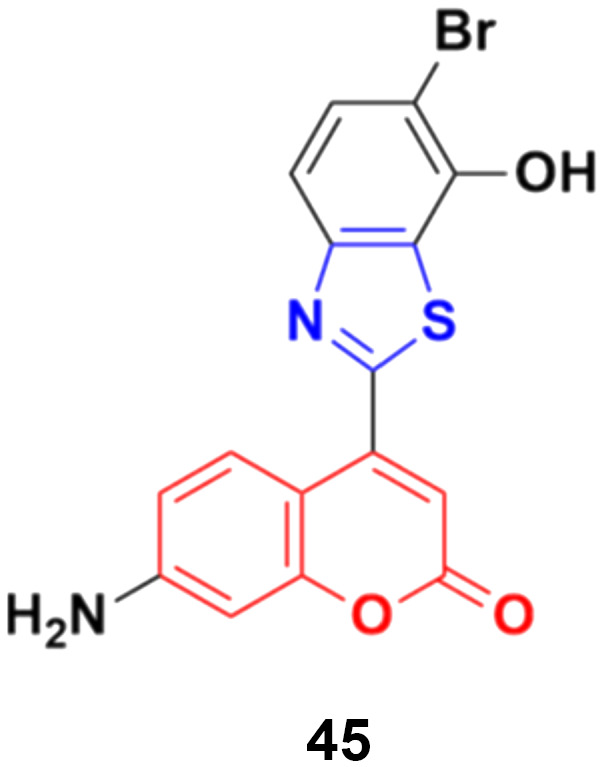
Chemical structure of coumarin–benzothiazole hybrid 45.

Coumarin–benzothiazole hybrids were screened for their antitumor activity at a single dose (10 μm) against a panel of 60 cancer cell lines.^91^ The most active compound 45 (Fig. 40) was further screened at a five-dose level. It displayed half-maximal growth inhibition (GI50) values of 0.24 and 0.33 μm against the central nervous system (CNS) cancer (SNB-75) and ovarian cancer (OVCAR-4) cell lines, respectively.

**Fig. 41:**
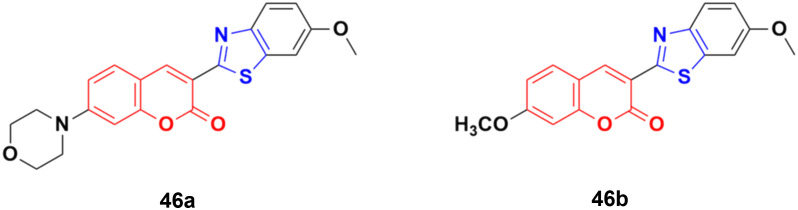
Chemical structures of coumarin–benzothiazole derivative 46a and b.

Compounds 46a and 46b (Fig. 41) were tested against the most common secondary ALK mutants such as L1196M, G1269A, and G1202R.^92^ Compound 46a showed potent inhibitory activities against three ALK mutants, L1196M, G1269A, and G1202R, with IC_50_ of 0.27 μM, 0.30 μM, and 0.59 μM, respectively. Compound 46b displayed an IC_50_ value of 0.45 μM for L1196M and compatible enzymatic inhibitory activity against G1269A and G1202R with that against WT ALK.

**Fig. 42:**
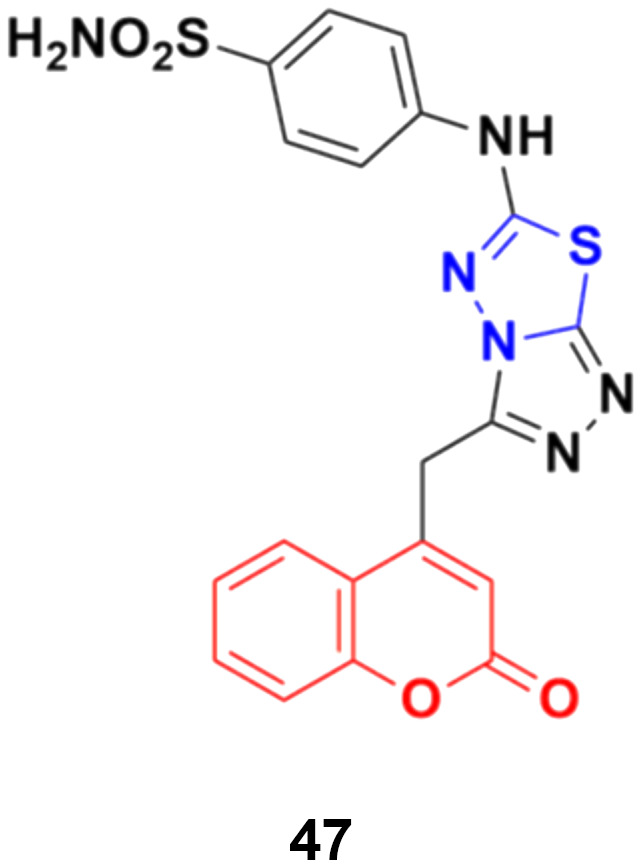
Chemical structure of coumarin–thiadiazole hybrid 47.

A series of novel coumarin derivatives having 1,2,4-triazolo[3,4-*b*][1,3,4]thiadiazole moieties was developed and analyzed *in vitro* for their anticancer activity against the HCT116 cell line.^93^ Compound 47 (Fig. 42) possessed significant anticancer activity with an IC_50_ value of 2.656 μM. Molecular docking studies suggested its possible interaction with tyrosine kinases (CDK2).

**Fig. 43:**
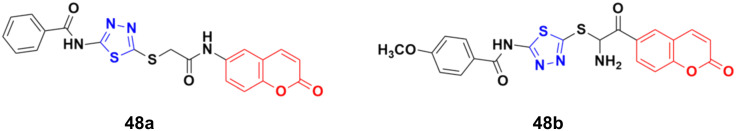
Chemical structures of coumarin–thiadiazole derivative 48a and b.

A novel series of coumarin–thiadiazole hybrids was developed using nucleophilic substitution reaction and their anticancer activity was tested against HCT-116, MCF-7, and HepG2 but none of them were more potent than the reference *cis*-platin.^94^ Compound 48a (Fig. 43) showed maximum potency against the HCT-116 and MCF-7 cell lines with IC_50_ values of 30.7 and 54.9 μg mL^−1^, respectively, while 48b was the most potent against HepG2 with an IC_50_ value of 24.9 μg mL^−1^.

**Fig. 44:**
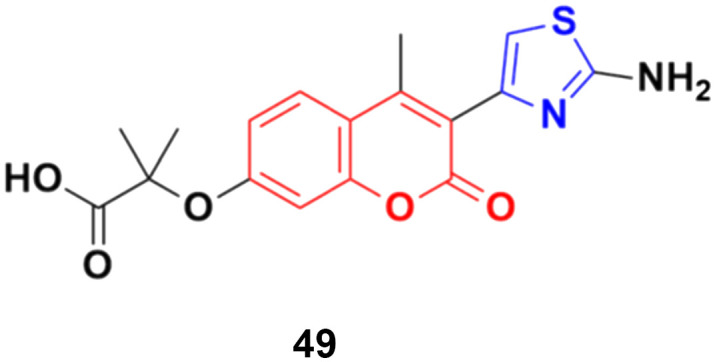
Chemical structure of coumarin–thiazole derivative 49.

A series of 2-(3-substituted-4-methyl-2-oxo-2*H*-chromen-7-yloxy)-2-methylpropanoic acid derivatives was developed by base-catalyzed dehydrohalogenative cyclization followed by Hantzsch synthesis and their *in vitro* cytotoxicity examined against the MCF-7, MDA-231, and HT29 cancer cell lines.^95^ Thiazole derivative 49 (Fig. 44) possessed the maximum potency against the MDA-231 and MCF-7 cell lines with IC_50_ values of 4.84 ± 0.17 and 2.39 ± 0.03 μM, respectively.

**Fig. 45:**
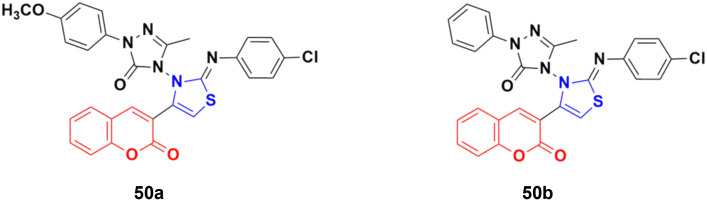
Chemical structures of coumarin–thiazole derivative 50a and b.

A series of coumarin-3-yl-thiazol-3-yl-1,2,4-triazolin-3-ones was synthesized using a microwave-assisted multicomponent protocol and their anticancer activity investigated against four cancer cell lines, including A549, MDA-MBA-231, HeLa and K562.^96^ Most of the derivatives showed better or comparative cytotoxic effects against all the cancer cell lines compared to the reference doxorubicin. Among them, compound 50a (Fig. 45) was more potent against three of the four cancer cell lines with IC_50_ = 0.16 μM against MDA-MBA-231, IC_50_ = 0.17 μM against A549, IC_50_ = 0.31 μM against K562, and IC_50_ = 0.25 μM against the HeLa cell line. Compound 50b showed the maximum efficiency against the HeLa cell line with IC_50_ = 0.21 μM.

**Fig. 46:**
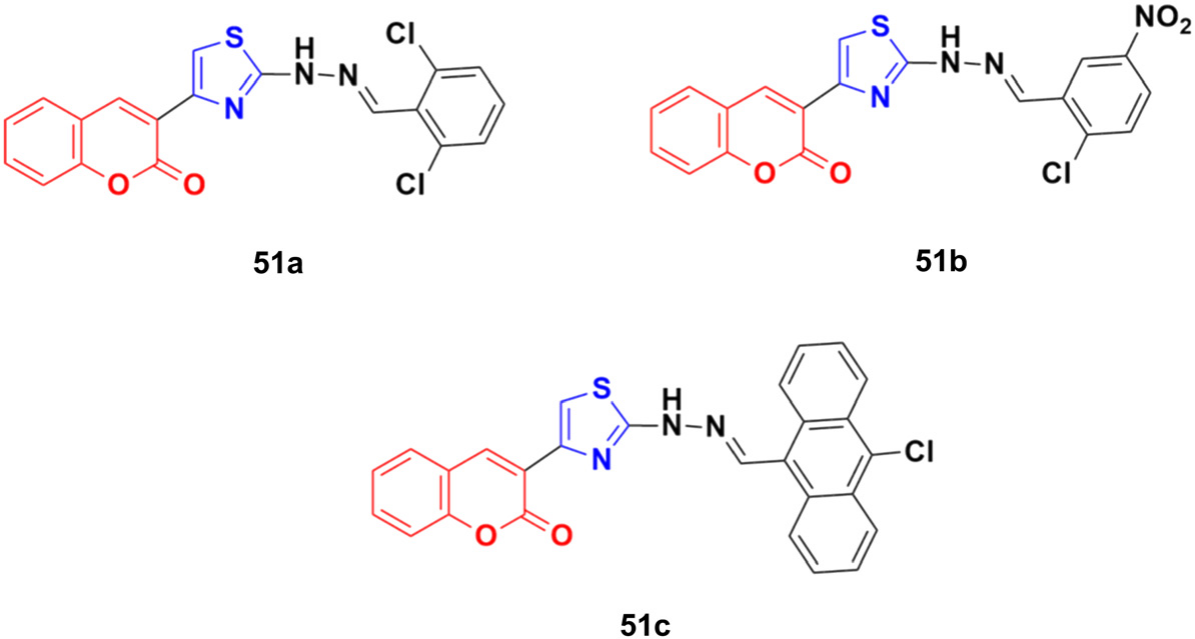
Chemical structures of coumarin–thiazole derivative 51a–c.

The anticancer activity of a series of coumarin–thiazole derivatives was screened *in vitro* against two cancer cell lines (HeLa and COS-7) together with a normal cell line (W138).^97^ Three compounds, 51a–c (Fig. 46), showed better cytotoxic ability than doxorubicin. Also, 51c possessed the best potency against the HeLa cell line with IC_50_ = 1.29 μM, while 51b and 51c showed significant cytotoxicity against COS-7 (IC_50_ = 1.96 and 1.66 μM, respectively). *In silico* studies revealed that the compounds meet the optimal needs for good oral absorption with no expected toxicity hazards.

**Fig. 47:**
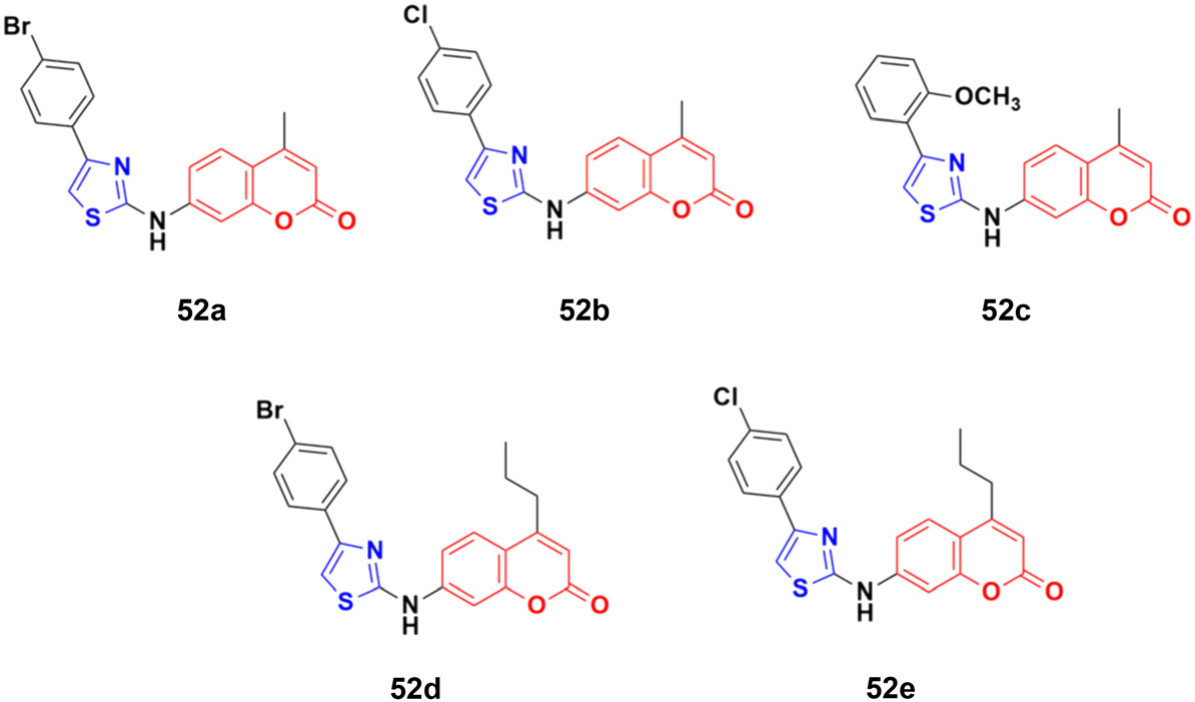
Chemical structures of coumarin–aminothiazole derivative 52a–e.

The coumarin–aminothiazole hybrids were examined for their cytotoxic activity against the HCT-116 and HT-29 cancer cell lines.^98^ Among them, compounds 52a–e (Fig. 47) were effective against both cell lines with IC_50_ values in the range of 0.25 to 0.38 μM. However, compound 52d was the most potent with IC_50_ = 0.25 ± 0.004 μM against HT-29 and IC_50_ = 0.26 ± 0.016 μM against HCT-116. Further biological investigation of 52a using Western blotting, caspase activity, glucose uptake, ROS production, and NADPH/NADP levels showed the ability of this lead drug candidate to induce cancer cell death *via* energy restriction. Moreover, the assessment of the synergistic activity of 52a with cisplatin showed promising outcomes.

**Fig. 48:**
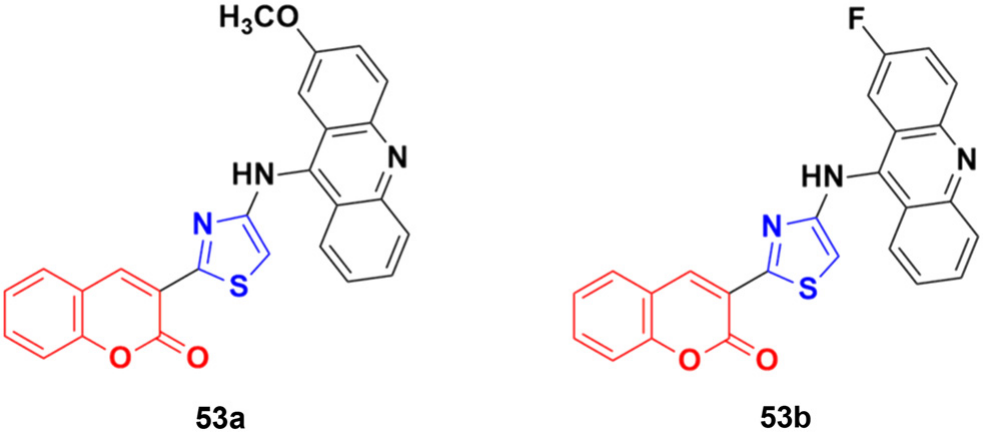
Chemical structures of coumarin–acridine–thiazole derivative 53a and b.

A series of nine novel acridine–thiazole bridged coumarin derivatives was prepared and evaluated for their *in vitro* antiproliferative activity on MDA-MB-231, A-549, and HT29 cell lines.^99^ All the compounds showed a significant cytotoxicity effect but none of them were more potent than the reference cisplatin. Compound 53a (Fig. 48) showed the maximum efficacy against the MDA-MB-231 cell line with IC_50_ = 8.03 ± 0.81 μM. Compound 53b was significantly active against A-549 and HT-29 with IC_50_ values 5.18 ± 1.04 μM and 23.09 ± 1.17 μM, respectively.

**Fig. 49:**
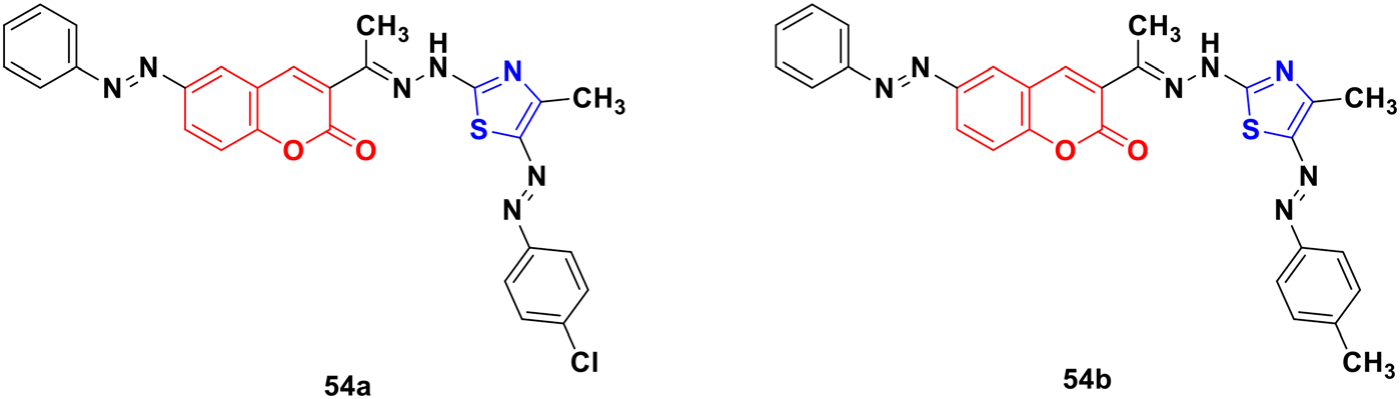
Chemical structures of coumarin–thiazole derivative 54a and b.

Several coumarin–thiazole derivatives were designed and synthesized and their cytotoxicity assessed on MCF-7 cancer cell lines using sorafenib as the reference drug.^100^ Among them, 54a and 54b (Fig. 49) demonstrated higher anticancer activities (IC_50_ = 10.5 ± 0.71 and 11.2 ± 0.80 μM, respectively) than sorafenib (IC_50_ = 5.10 ± 0.49 μM). These hybrids are thought to inhibit the vascular endothelial growth factor receptor (VEGFR-2) signaling system.

#### 2.2.6. Miscellaneous coumarin-azole hybrids

**Fig. 50:**
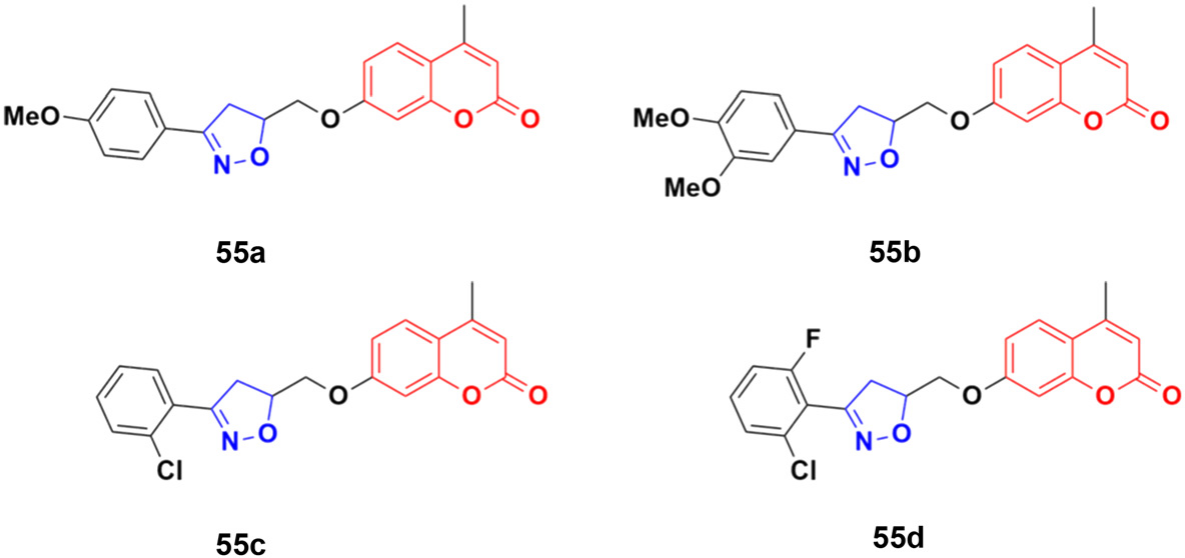
Chemical structures of coumarin-isoxazoline derivative 55a–d.

A series of twelve coumarin-tethered isoxazolines was designed and analyzed for their antiproliferative activity against the human melanoma cancer cell line (UACC 903) and fibroblast normal cell line (FF2441).^101^ Among them, compounds 55a–d (Fig. 50) showed significant efficiency against human melanoma cancer (UACC 903) with IC_50_ values of 8.8, 10.5, 9.2, and 4.5 μM, respectively. The non-toxic compound 55b, which was regarded as the lead, significantly decreased the cell survival, body weight, and ascites volume and downregulated the formation of neovasculature such as the deterioration of tumor volume.

**Fig. 51:**
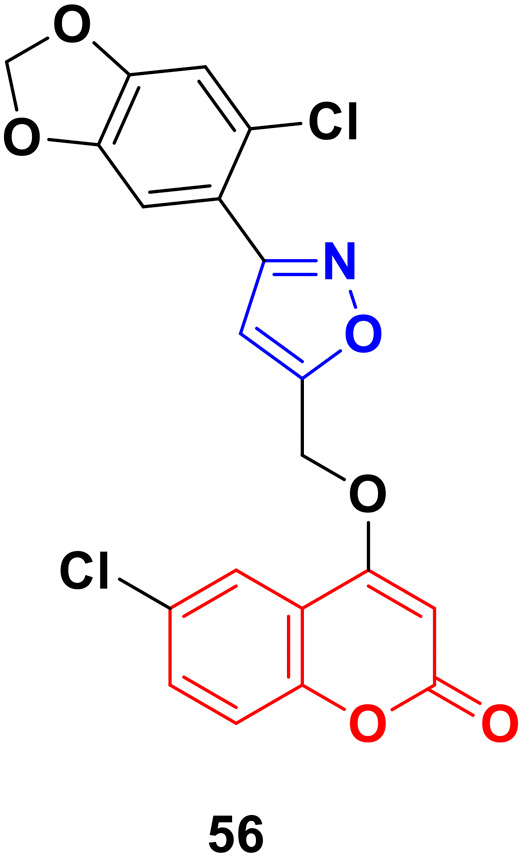
Chemical structure of coumarin–isoxazole derivative 56.

A series of coumarin–isoxazole derivatives was synthesized from imidoyl chlorides and various substituted 4-(prop-2-yn-1-yloxy)-2*H*-chromen-2-one and their biological activity evaluated against the HepG2 cell line.^102^ Among them, 56 (Fig. 51) possessed the best activity (IC_50_ = 12.85 μM L^−1^) against the HepG2 cell line. Its toxicity against Vero cells (IC_50_ = 144.32 μM L^−1^) was lower than that of *cis*-platin (IC_50_ = 28.63 μM L^−1^). It was observed that the presence of Cl or Br at the 6-position of the coumarin moiety enhances the bioactivity. The docking result showed that the compound can fruitfully interact with the protein.

**Fig. 52:**
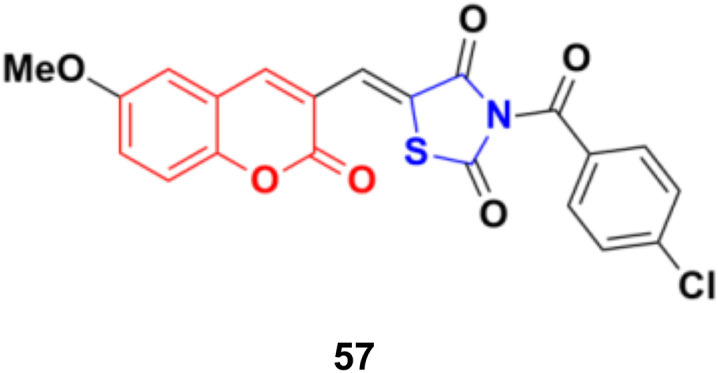
Chemical structure of coumarin–thiazolidin-2,4-dione hybrid 57.

A series of coumarin–thiazolidin-2,4-dione hybrids was tested for their anticancer activity against the MCF-7, HeLa, HT29, A549, and PC3 cancer cell lines, but none of them were more effective than the reference doxorubicin.^103^ Among them, compound 57 (Fig. 52) was the most potent against the MCF-7, HeLa, and A549 cell lines with IC_50_ values in the range of 0.95 to 3.20 μM.

**Fig. 53:**
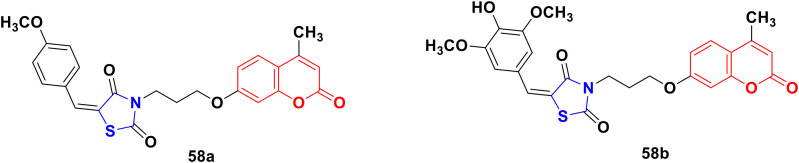
Chemical structures of coumarin–thiazolidinone 58a and b.

A novel series of coumarin–thiazolidinone derivatives was synthesized using the coupling methodology and their *in vitro* cytotoxicity screened against the MCF-7 cancer cell line.^104^ Among them, compounds 58a and 58b (Fig. 53) possessed noteworthy activity with IC_50_ values of 15.65 ± 0.28 μg mL^−1^ and 12.15 ± 0.05 μg mL^−1^, respectively. The structure–activity relationship studies indicated that the presence of an electron-releasing methoxy group increases the cytotoxic activity. Kinase inhibition and suitable binding are responsible for their significant biological property.

### 2.3. Coumarin–furoxan hybrids

**Fig. 54:**
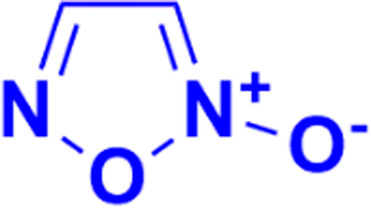
Chemical structure of furoxan.

Furoxan (1,2,5-oxadiazole-2-oxide), a nitric oxide donor, is a heterocycle of the isoxazole family and an amine oxide derivative of furazan (Fig. 54). Nitric oxide plays an essential role in cardiovascular regulation, nerve transmission delivery, and immune response, and a high concentration of nitric oxide has potentially significant anticancer effects. Hence, the hybridization of coumarin and furoxan may be a lead for generating new anticancer agents.

**Fig. 55:**
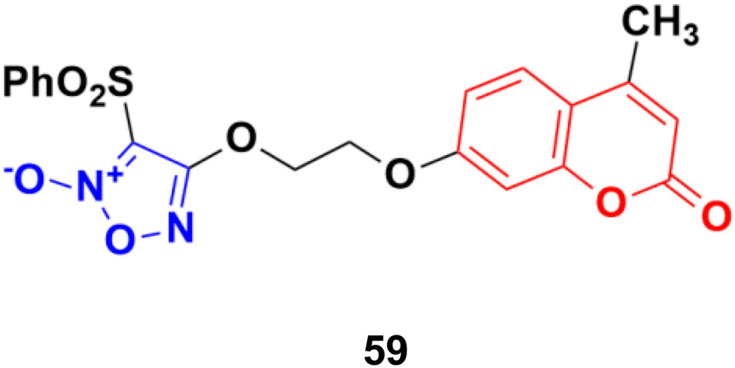
Chemical structure of coumarin–furoxan hybrid 59.

A series of sixteen furoxan-based coumarin derivatives was synthesized and their antiproliferative activities investigated against several cancer cell lines including A549, HeLa, A2780, A2780/CDDP, and HUVEC.^105^ They all showed moderate to excellent anticancer activity against the above-mentioned cell lines, but compound 59 (Fig. 55) was observed to be the most potent with IC_50_ values of 0.024, 0.053, 0.014, 0.062, and 0.034 μM, respectively. This compound was again investigated for its biological activity against some drug-resistant cell lines (MDA-MB-231, MDA-MB-231/Gem, SKOV3, and SKOV3/CDDP) and proved to be very effective against them also. Furthermore, 55 inhibited the growth of A2780 *in vivo* and displayed lower toxicity on non-tumorigenesis T29. Preliminary pharmacological studies revealed that 59 acts by inducing apoptosis, arresting the cell cycle at the G2/M phase in the A2780 cell line, and disrupting the phosphorylation of MEK1 and ERK1. Compound 59 was further studied to reveal its potential for apoptosis and autophagy induction in lung adenocarcinoma cells.^106^ The cytotoxicity and apoptosis of A549 and H1299 cells induced by compound 59 were detected by MTT, microscopy, and western blot analysis. Significant growth inhibition and caspase-dependent apoptosis were observed in the compound 59-treated A549 and H1299 cells. Then, it was confirmed that this compound induced autophagy by autophagosome formation, upregulated the expression of autophagy-related protein LC3-II, and autophagic flux. Importantly, abolishing autophagy using inhibitors and ATG5 siRNA enhanced the cytotoxicity of compound 59, indicating the cytoprotective role of autophagy in lung adenocarcinoma. Further mechanistic investigations suggested that the Akt/mTOR and Erk signaling pathways contributed to autophagy induction by compound 59.

**Fig. 56:**
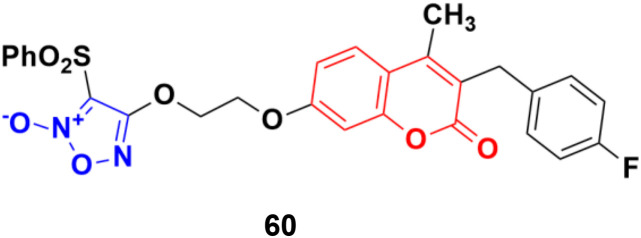
Chemical structure of 3-benzylcoumarin–furoxan hybrid 60.

Five phenylsulfonylfuroxan-merging 3-benzyl coumarins were designed and evaluated for their anticancer activities.^107^ Among them, compound 60 (Fig. 56) showed the most potent antiproliferation activities with IC_50_ values ranging from 0.5 nM to 143 nM against nine drug-sensitive (HeLa, SKOV3, A549, OVCA429, OVCA433, A2780, MDA-MB-231, MCF-7 and KB) and four drug-resistant cancer cell lines (A2780/CDDP, MDA-MB-231/Gem, MCF-7/ADR, and KB-V). Preliminary pharmacological studies revealed that compound 60 acts by inducing early apoptosis and affecting the cell cycle. Furthermore, it gave 559- and 294-fold selectivity antiproliferation activity in the P-gp overexpressed drug-resistant cancer cell lines MCF-7/ADR and KB-V compared to the drug-sensitive MCF-7 and KB, implying that this compound may have an extra mechanism of anti-MDR-cancer with P-gp overexpression. Here, compound 60 contains fluorine. It is noteworthy to mention that fluorine-containing drugs are tremendously important and are successfully being used in the treatment of many diseases, *e.g.*, multiple myeloma, lymphoma, HIV, chronic heart failure, chronic myeloid leukemia, (ANCA)-associated vasculitis, migraines, von Hippel–Lindau disease, and non-small cell lung cancer.^108–115^

**Fig. 57:**
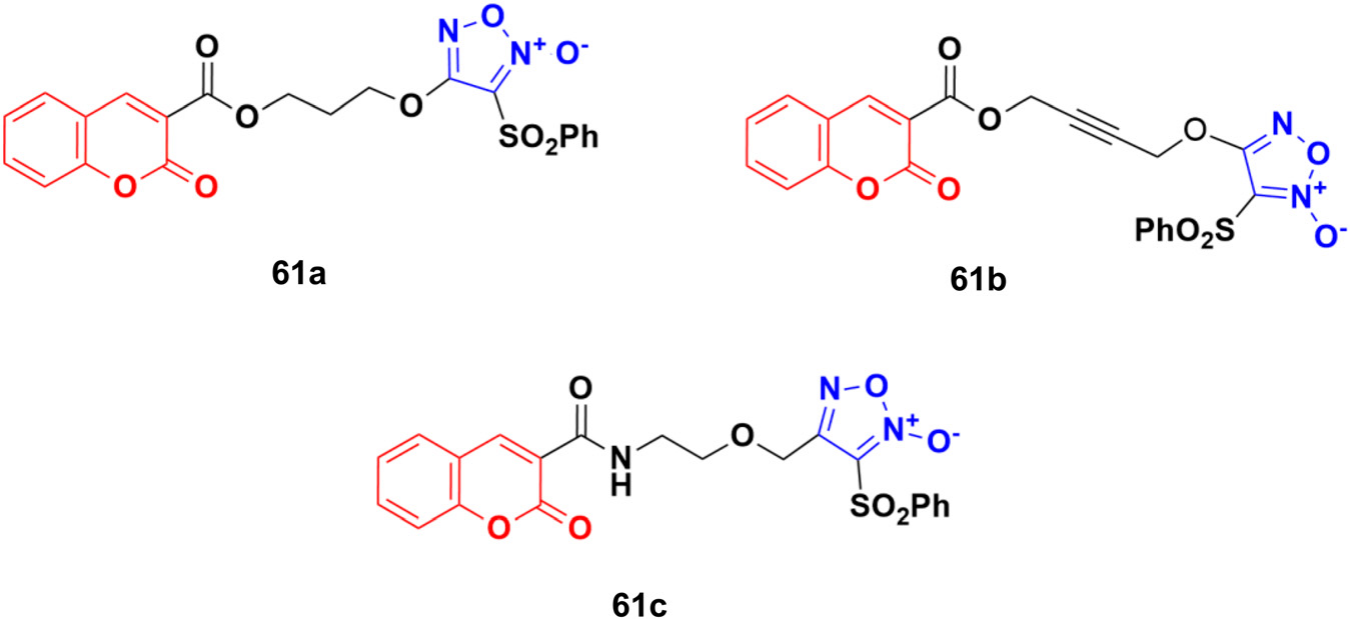
Chemical structures of coumarin–furoxan hybrid 61a–c.

A novel series of eleven furoxan–coumarin hybrids was developed and their antiproliferative activity studied on five human cancer cell lines including HeLa, SW620, HepG2, HCT116, and MCF7.^116^ Among them, compounds 61a–c (Fig. 57) showed potent anticancer activity and some evaluated to be more potent than the reference doxorubicin. Compound 61a was the most potent against the HepG2 cell line with IC_50_ = 3.86 μM, while compound 61b showed the maximum activity against the SW620 and HCT116 cell lines with IC_50_ values of 1.86 and 3.46 μM, respectively. Compound 61c was observed to be the most effective against the HeLa and MCF7 cell lines with IC_50_ values of 0.88 and 0.61 μM, respectively.

**Fig. 58:**
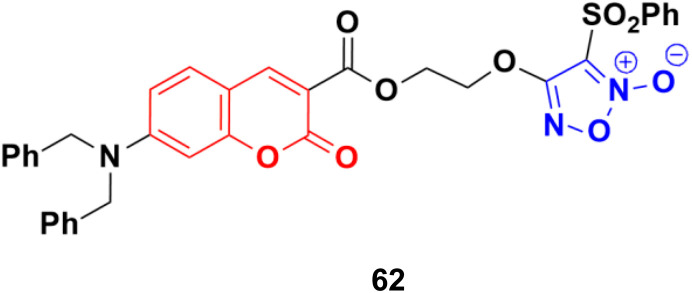
Chemical structure of coumarin–furoxan hybrid 62.

A series of furoxan conjugates of *N*,*N*-dialkyl carboxy coumarins was developed as potential anticancer agents and tested for their *in vitro* antiproliferative activities on various cell lines (MDA-MB-231, 4T1, WiDr, MCF10A, and HDFa cell lines).^117^ Among them, compound 62 (Fig. 58) showed the highest potency with IC_50_ values in the range of 0.02 to 38.6 μM. The *in vitro* mechanistic studies indicated that these compound generated substantial nitric oxide, inhibited colony formation, and caused apoptosis in cancer cells.

### 2.4. Coumarin–pyridine/pyrimidine hybrids

**Fig. 59:**
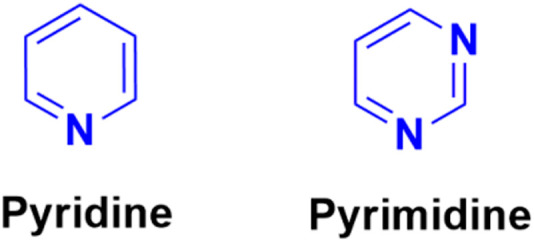
Chemical structures of pyridine and pyrimidine.

Pyridine (Fig. 59), one of the most basic heterocyclic compounds with widespread biological occurrence, forms the nucleus of numerous drugs. Pyridine derivatives are known to possess a variety of biological activities, namely, anti-asthmatic, antibacterial, anticonvulsant, antimalarial, antimuscarinic, antiprotozoal, anticancer, antidiabetic, and anti-inflammatory.

Similarly, pyrimidine (Fig. 59), which is structurally related to pyridine, the building unit of DNA and RNA, has also been found to possess marked pharmacological effects.

**Fig. 60:**
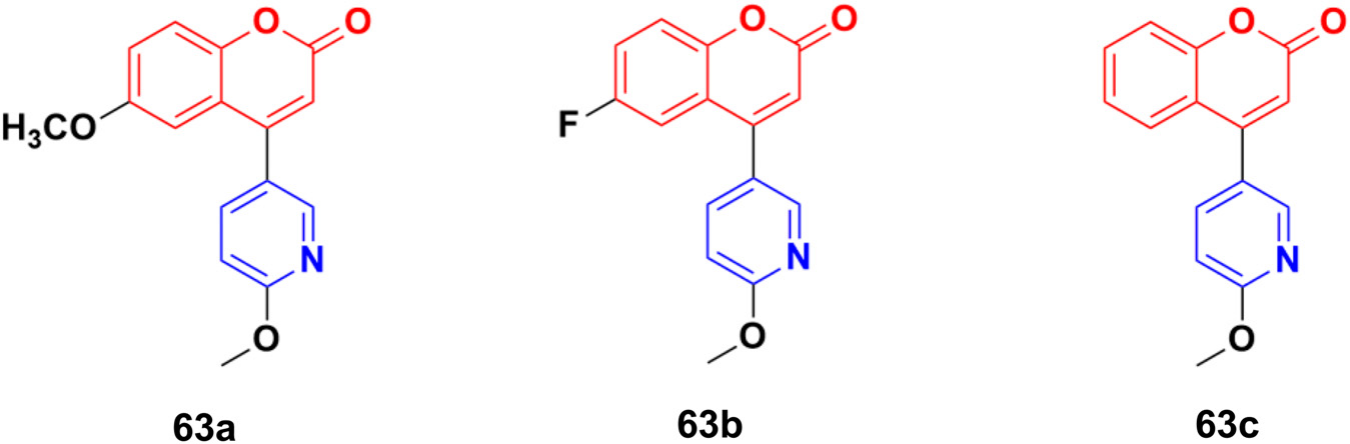
Chemical structures of coumarin–pyridine hybrid 63a–c.

The coumarin–pyridine hybrids 63a–c (Fig. 60) showed weak to moderate activity against the A549 cancer cell line with IC_50_ values in the range of 34.2 > 80 μM.^118^ The compounds were found to potently inhibit *in vitro* microtubule formation *via* a substoichiometric mode such as CA-4.

**Fig. 61:**
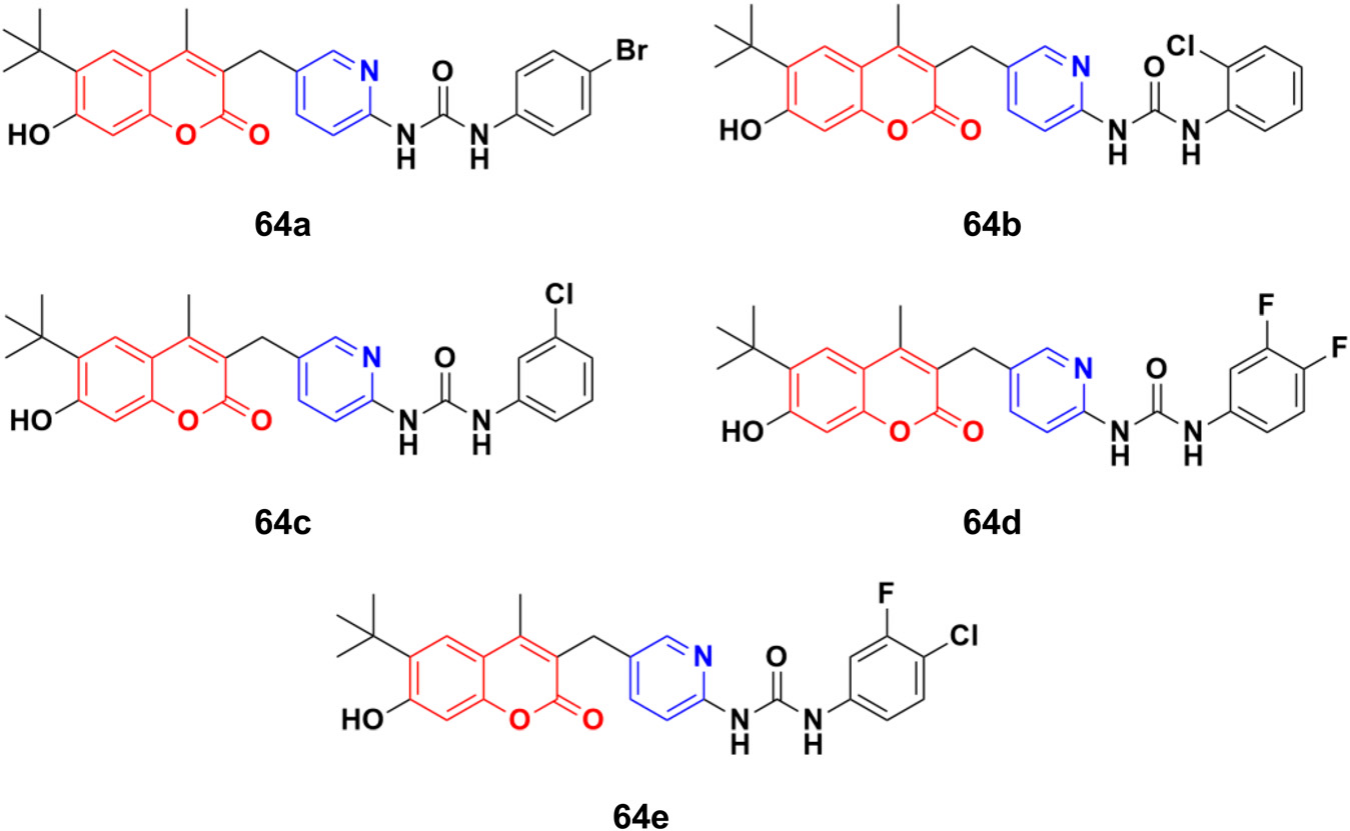
Chemical structures of coumarin–pyridine hybrid 64a–e.

A novel series of thirteen coumarin–pyridine derivatives was designed and their cytotoxic activities examined on four cancer cell lines including K562, HeLa, A549, and MCF7.^119^ Among them, compounds 64a–e (Fig. 61) showed the maximum potency against the MCF7, A549, HeLa, and K562 (64d and e) cell lines with IC_50_ values of 2.56 ± 0.17, 4.38 ± 0.09, 2.17 ± 0.45, 1.66 ± 0.09 and 1.66 ± 0.15 μM, respectively. Further investigation revealed that compounds 64a and 64c were much more potent PI3K inhibitors than S14161 and BENC-511 (reference). In addition, 64a was more selective to PI3Kα/β over PI3Kδ/γ, while 64c was a selective PI3Kα/β/δ inhibitor. 64c could also suppress the phosphorylation of Akt and induce K562 cell apoptosis.

**Fig. 62:**
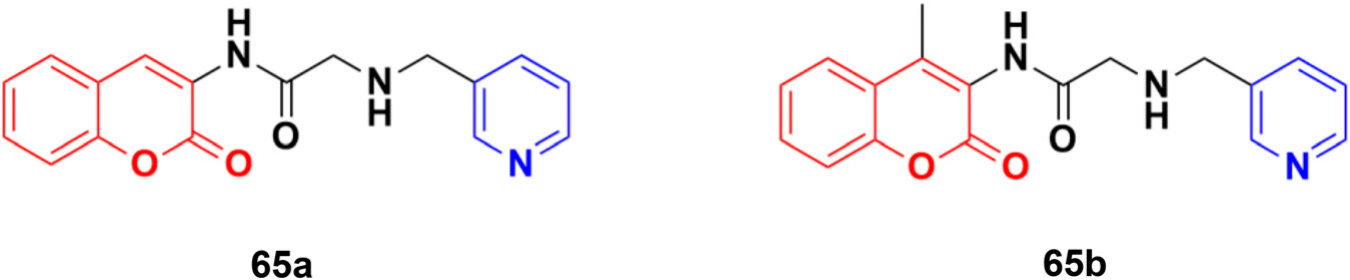
Chemical structures of coumarin–pyridine hybrid 65a and b.

Some other coumarin–pyridine hybrids such as 65a and b (Fig. 62) (IC_50_: 69.1–377.8 mM against both A549 and MCF-7 cancer cell lines, MTT assay) were also active against the tested cancer cell lines, but most of them were much less potent than the references.^120–124^

**Fig. 63:**
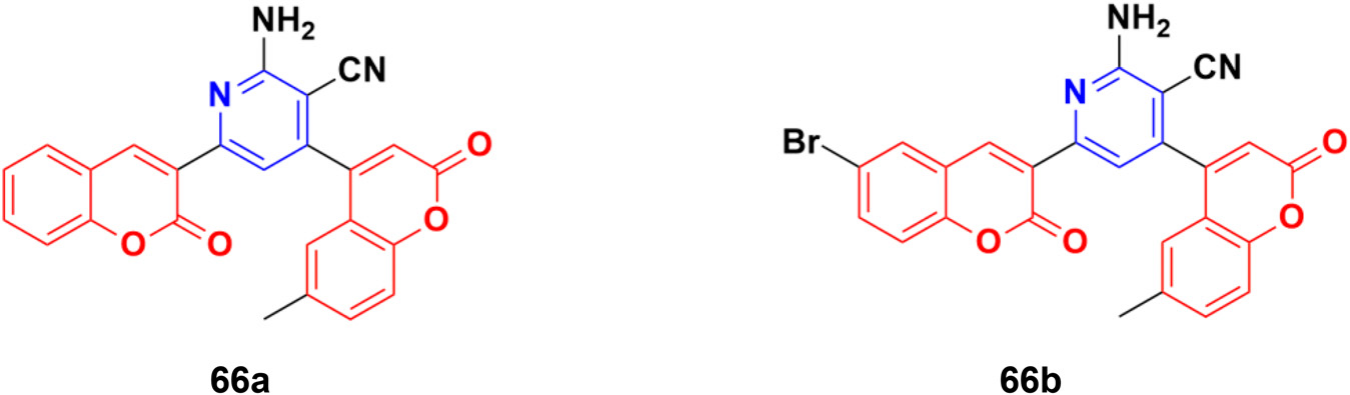
Chemical structures of coumarin–pyridine hybrid 66a and b.

A novel series of fifteen coumarin–pyridine hybrids was synthesized *via* a one-pot four-component coupling reaction under a neat microwave method and their antiproliferative properties investigated against several cancer cell lines.^125^ Among them, compounds 66a and 66b (Fig. 63) exhibited promising anticancer activity at low concentrations (10^−5^ M) against the NCI-60 cell line. These two potent anticancer molecules were screened for their CT-DNA cleavage and fluorescence quenching with BSA transport protein.

**Fig. 64:**
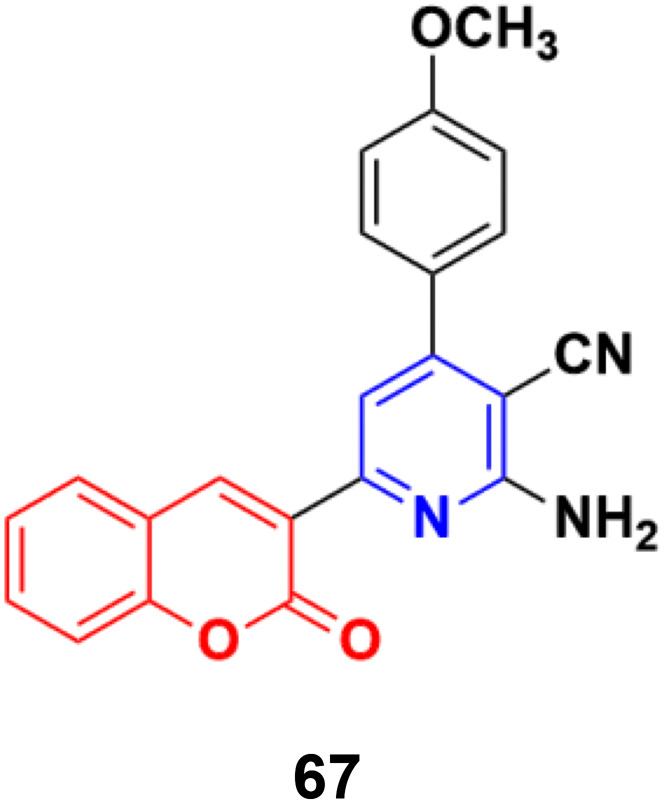
Chemical structures of coumarin–pyridine hybrid 67.

The similar synthesis of coumarin–pyridine conjugates was accomplished *via* conventional heating and microwave irradiation and the designed compounds were tested for their *in vitro* cytotoxicity.^126^ The preliminary screening results showed that most of the compounds had moderate cytotoxic activity against the HCT-116 and MCF-7 cell lines, although compound 67 (Fig. 64) exhibited potent activity against both cell lines with IC_50_ values of 9.9 ± 0.82 and 14.1 ± 1.14 μM, respectively, which was comparable with the standard drug 5-fluorouracil.

**Fig. 65:**
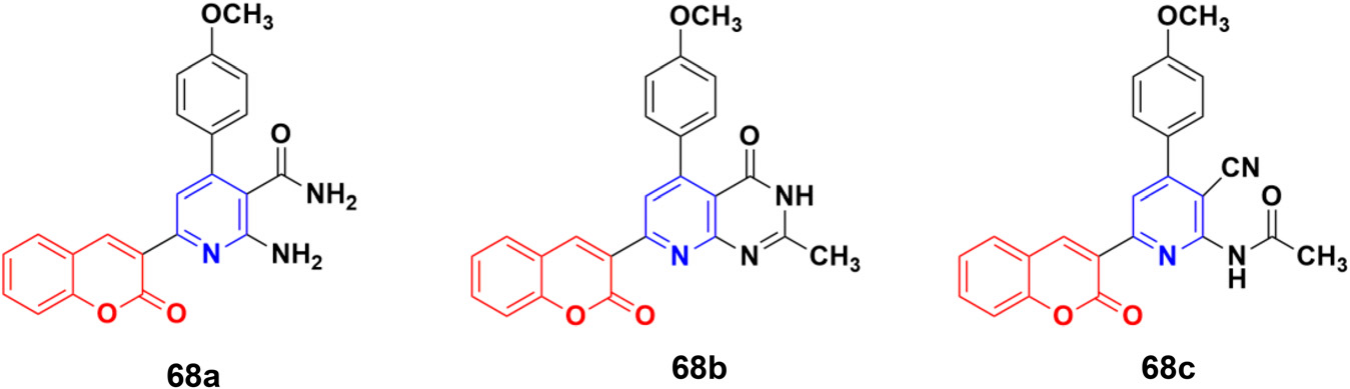
Chemical structures of coumarin–pyridine hybrid 68a–c.

A series of coumarin–pyridine hybrids was synthesized and their anticancer activity evaluated against the MCF-7, HCT-116, HepG-2, and A549 human cancer cell lines.^127^ Among them, compounds 68a–c (Fig. 65) showed the most potent growth inhibitory activities with IC_50_ values in the range of 1.1 to 2.4 μM against the MCF-7 cell line. Flow cytometric analysis revealed that these compounds induced cell cycle arrest in the G2/M phase followed by apoptotic cell death. Furthermore, the activity of caspase-3 in MCF-7 cells was tested. The results indicated that compounds 68a–c increased the caspase-3 activity significantly compared to the control group.

**Fig. 66:**
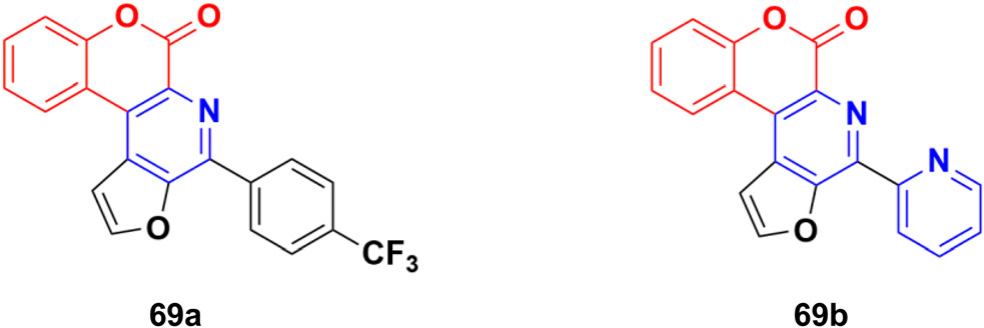
Chemical structures of coumarin–pyridine hybrid 69a and b.

Several thio/furo-fused pyridine moieties having a coumarin scaffold were synthesized using an FeCl_3_-catalyzed modified Pictet–Spengler reaction as the crucial final step and their biological activities were evaluated against three cancer cell lines including DU145, B16F10, and MCF-7.^128^ Compound 69a (Fig. 66) possessed significant anticancer activity against the DU145 and B16F10 cell lines with IC_50_ values of 20.88 and 12.98 μM, respectively, whereas compound 69b was the most potent against the MCF-7 cell line (IC_50_ = 8.00 μM).

**Fig. 67:**
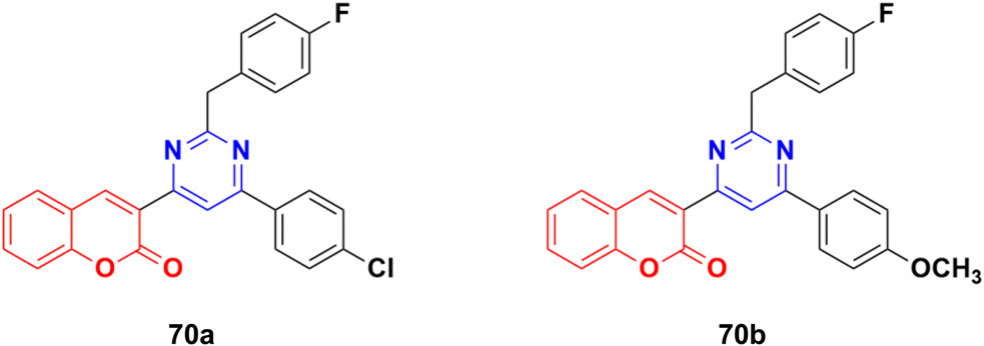
Chemical structures of coumarin–pyrimidine hybrid 70a and b.

A series of twelve coumarin–pyrimidine conjugates was synthesized under microwave irradiation and their cytotoxic activities evaluated against the A-549 and MDA-MB-231 cancer cell lines.^129^ Some of them were observed to be more potent than the reference *cis*-platin. Compound 70a (Fig. 67) showed the maximum potency against A549 with IC_50_ = 2.15 ± 0.12 μM, while 70b was the most potent against the MCF-7 cell line with IC_50_ = 2.23 ± 0.19 μM. The DNA cleavage study by the gel electrophoresis method revealed that compounds 70a and b inhibited the growth of the pathogenic organism by cleaving the genome given that no traces of DNA were found.

**Fig. 68:**
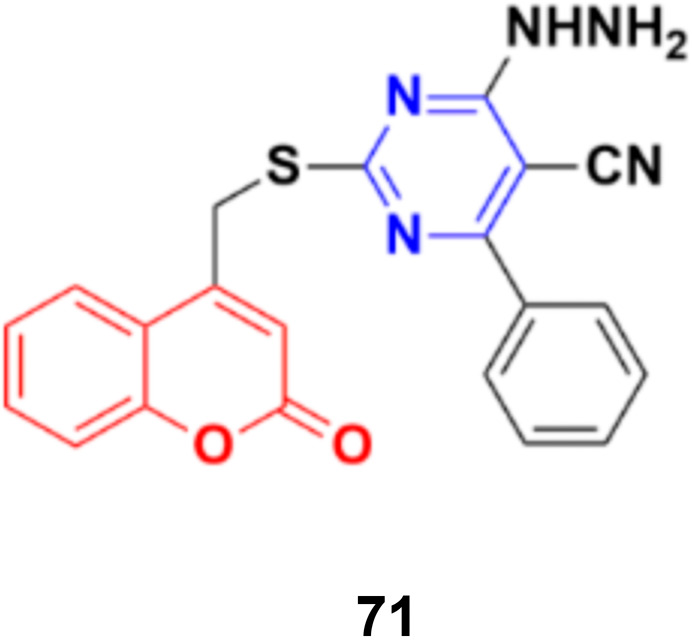
Chemical structure of coumarin–pyrimidine hybrid 71.

Coumarin–pyrimidine hybrid 71 (Fig. 68) showed significant antiproliferative activity against the HePG2 and MCF-7 cancer cell lines with IC_50_ values of 5.5 ± 0.19 and 6.9 ± 0.38 μg mL^−1^, respectively.^130^

**Fig. 69:**
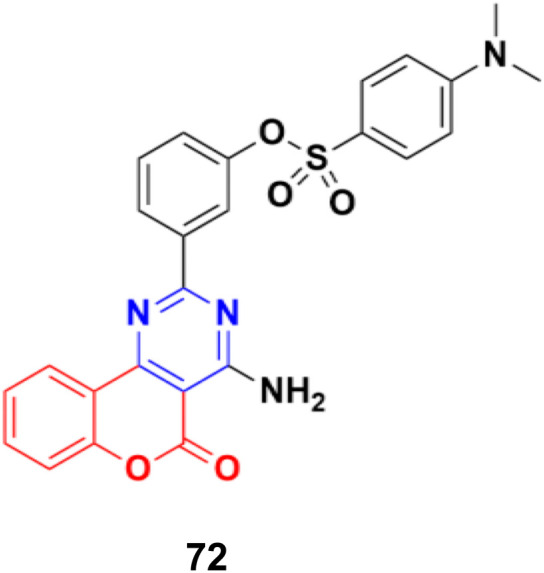
Chemical structure of coumarin–pyrimidine hybrid 72.

A series of 2-phenylpyrimidine coumarin derivatives with potential telomerase-inhibiting activity was designed and synthesized and all the compounds were screened for their antiproliferative activity against the CNE2, KB, and Cal27 cell lines *in vitro*.^131^ Among them, compound 72 (Fig. 69) exhibited the best activity (IC_50_ = 1.92 ± 0.13, 3.72 ± 0.54, and 1.97 ± 0.51 against the CNE2, KB, and Cal27 cell lines, respectively). Flow cytometry revealed that this compound can inhibit CNE2 proliferation. The molecular docking results indicated that compound 72 bonded with telomerase reverse transcriptase (TERT) through multiple interactions, including hydrogen bonding and hydrophobic interactions.

**Fig. 70:**
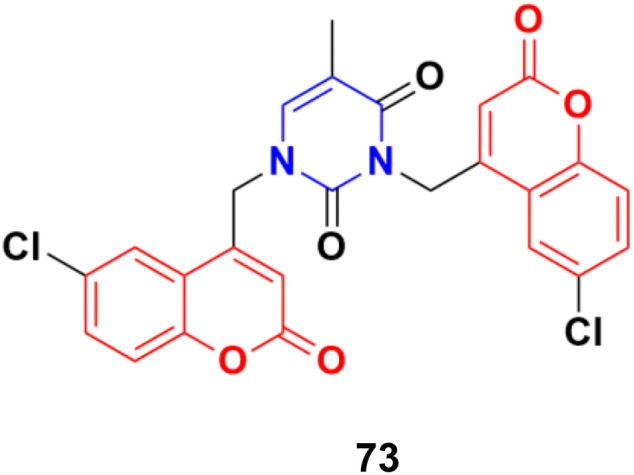
Chemical structure of coumarin–pyrimidine hybrid 73.

A series of C4–C4′ biscoumarin–pyrimidine hybrids was synthesized *via* S_N_2 reaction of substituted 4-bromomethyl coumarin with thymine and screened for *in vitro* anticancer activity against C6 rat glioma cells.^132^ Among the screened compounds, compound 73 (Fig. 70) was recognized to be the best antiproliferative candidate, having an IC_50_ value of 4.85 μM. All the compounds were found to be nontoxic toward healthy human embryonic kidney cells (HEK293). Furthermore, compound 73 displayed strong binding interactions with the drug carrier protein, human serum albumin, and exhibited good solution stability at biological pH conditions.

**Fig. 71:**
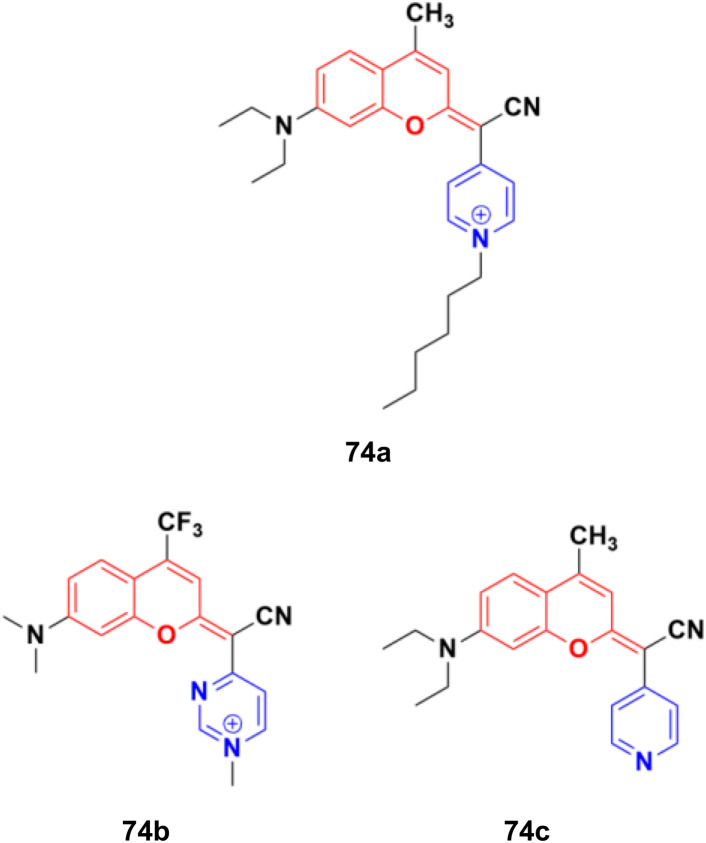
Chemical structures of potential PTT COUPY 74a–c.

A fascinating family of low-molecular-weight coumarins (COUPYs) was developed, in which the carbonyl group of the lactone function of the classical coumarin scaffold was replaced by cyano(4-pyridine/pyrimidine)methylene moieties, and investigated as potential photodynamic therapy (PTT) anticancer tools.^133^ Among them, three compounds, 74a–c (Fig. 71), exhibited effective *in vitro* anticancer activities upon visible-light irradiation under both normoxia and hypoxia (phototherapeutic index of up to 71) and minimal toxicity toward normal cells. In addition, their cytotoxicity was also evaluated in non-tumorigenic ovarian tissue-derived cells (CHO) to determine their differential selectivity for cancer cell lines. Compound 74a showed excellent cytotoxicity against the HeLa and A2780 cell lines with IC_50_ of values 0.19 ± 0.03, 0.09 ± 0.02 μM, respectively, with a selectivity factor of 15.6. Furthermore, compounds 74b and 74c were also effective against HeLa and A2780 with IC_50_ values 1.1 ± 0.1 and 1.1 ± 0.3 μM, respectively, but their selectivity factor was low, and even lower than the reference *cis*-platin. Acting as excellent theranostic agents targeting mitochondria, the mechanism of action of these compounds was investigated in detail in HeLa cells. The generation of cytotoxic ROS and induction of apoptosis and/or autophagy were identified as the cell death modes triggered after irradiation with low doses of visible light.

**Fig. 72:**
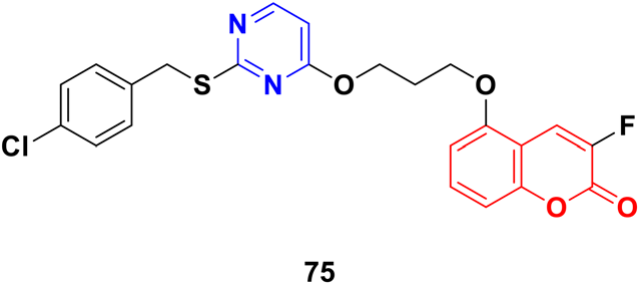
Chemical structure of coumarin–pyrimidine hybrid 75.

Coumarin-tagged pyrimidine scaffold 75 (Fig. 72) was found to selectively impede the proliferation of HER2-positive BC cells.^134^ It induced DNA damage and apoptosis in HER-2-positive BC cells more effectively compared to HER-2 negative BC cells. *In silico* and theoretical calculations revealed that compound 75 could interact with c-Jun N-terminal kinase (JNK), and *in vitro* studies showed that it increased JNK phosphorylation through ROS generation.

**Fig. 73:**
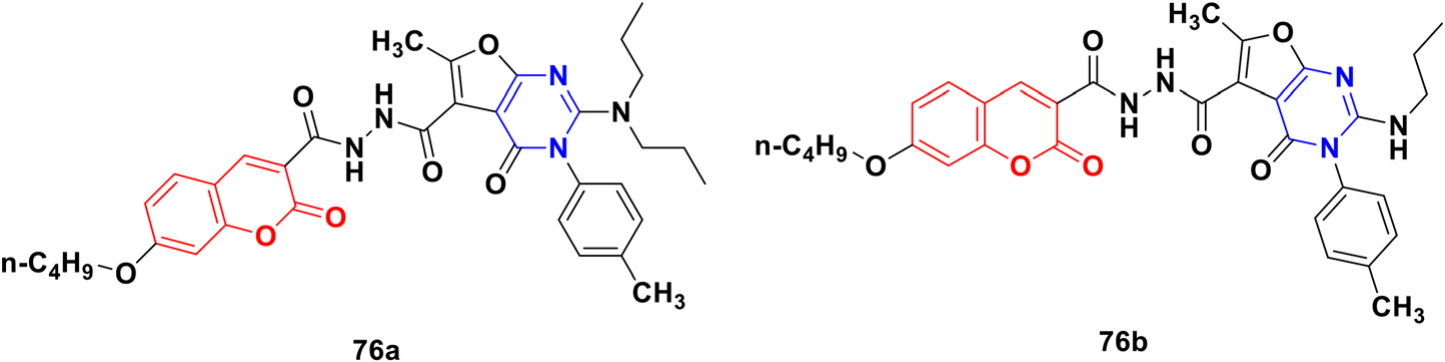
Chemical structures of coumarin-furo[2,3-*d*]pyrimidinone hybrid 76a and b.

A novel library of coumarin-furo[2,3-*d*]pyrimidinone hybrid derivatives was synthesized and assessed for their antiproliferative activities against the HepG2 and HeLa cell lines *in vitro*.^135^ Compound 76a (Fig. 73) showed maximum potency against HepG2 with an IC_50_ value of 4.87 μmol L^−1^. The kinase activity assay revealed that compound 76a may be a multi-target inhibitor. Alternatively, compound 76b was the most potent against the HeLa cell line with an IC_50_ value of 6.47 μmol L^−1^. The structure–activity relationship study showed that a more bulky and electro-positive group at the C-2 position the of furo[2,3-*d*]pyrimidinone ring enhanced the bioactivity.

### 2.5. Coumarin–indole/isatin hybrids

**Fig. 74:**
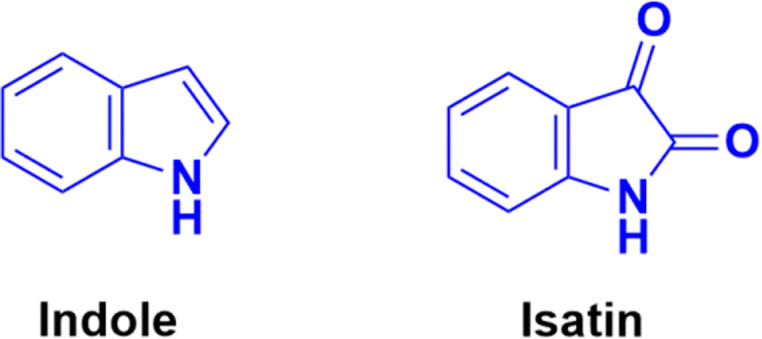
Chemical structures of indole and isatin.

Indole (Fig. 74), consisting of a six-membered benzene ring fused to a five-membered pyrrole ring, is an important structural scaffold of various drugs.^136,137^ More than 200 indole derivatives have already been marketed as drugs (*e.g.*, melatonin, indirubin, and sunitinib) or are in advanced stages of clinical trials. Similarly, isatin (Fig. 74), which is a derivative of indole, has also been observed to have efficient biological activity. This derivative possesses anticancer activities and can induce cell death.^138–140^ Hence, coumarin–indole/isatin hybrids may be important in the search for novel anticancer candidates.

**Fig. 75:**
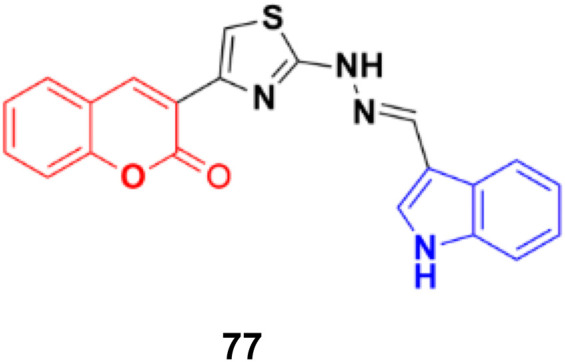
Chemical structure of coumarin–indole hybrid 77.

A series of indole-incorporated thiazolyl coumarins was developed and evaluated for their anticancer activities *in vitro*.^141^ Among them, compound 77 (NSC: 768621/1) (Fig. 75) showed excellent antiproliferative properties against the full panel of 60 human tumor cell lines. The five dose-level activity results revealed that compound 77 was active against all the cell lines. It showed potent activity against CCRFCEM (GI_50_: 0.33 μM), NCI-H522 (GI_50_: 1.03 μM), HCT116 (GI_50_: 1.60 μM), SF-539 (GI_50_: 1.58 μM), MALME-3 M (GI_50_: 1.59 μM), OVCAR-3 (GI_50_: 1.16 μM), UO-31 (GI_50_: 0.76 μM), PC-3 (GI_50_: 0.82 μM) and BT-549 (GI_50_: 1.13 μM).

**Fig. 76:**
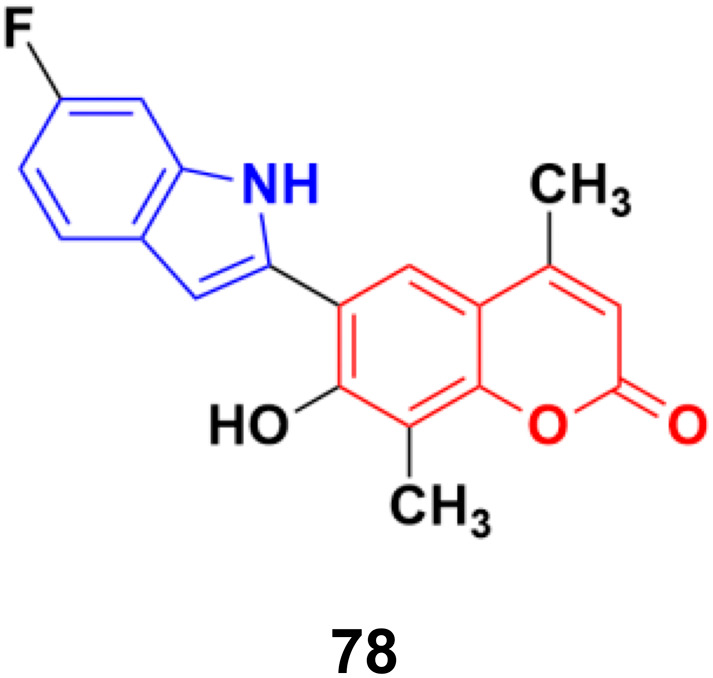
Chemical structure of coumarin–indole hybrid 78.

6-(6-Fluoro-1*H*-indol-2-yl)-7-hydroxy-4,8-dimethyl-2*H*-chromen-2-one (78), a coumarin–indole conjugate (Fig. 76), showed the highest level of antimitotic activity with mean GI_50_/TGI values of 3.28/13.24 mM and certain sensitivity profile towards the non-small cell lung cancer cell line HOP-92 (GI50/TGI/LC50 values 0.95/4.17/29.9 mM).^142^

**Fig. 77:**
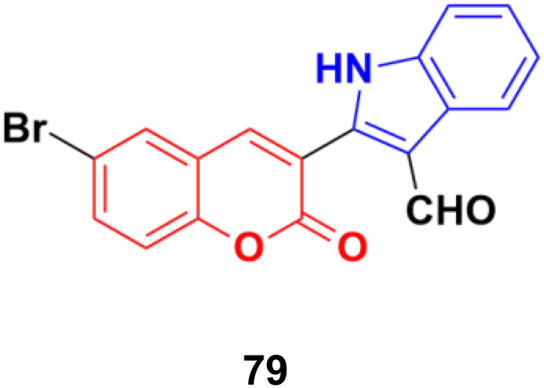
Chemical structure of coumarin–indole hybrid 79.

Another series of coumarin–indole derivatives was synthesized and their cytotoxic activities investigated *in vitro* against an MCF-7 cancer cell line together with a normal cell line.^143^ Among the characterized compounds, 79 (Fig. 77) showed the maximum potency against the MCF-7 cell line with IC_50_ = 7.4 μM. Flow cytometric cell cycle analysis of 79 exhibited the apoptotic mode of cell death due to cell cycle arrest in the G2/M phase.

**Fig. 78:**
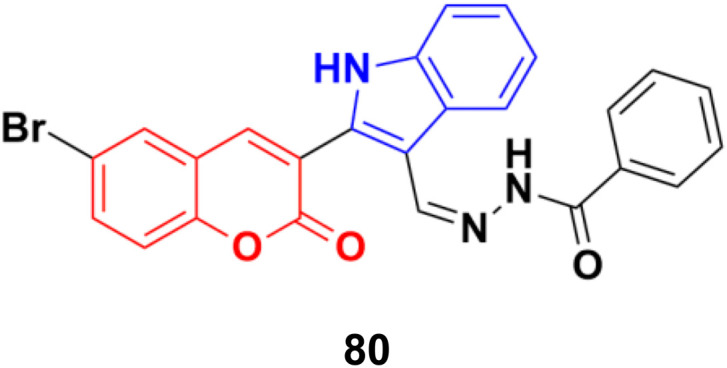
Chemical structure of coumarin–indole hybrid 80.

Further investigation revealed that hybrid 80 (Fig. 78) with a bromine atom in position-7 of the coumarin ring displayed excellent dose-dependent cytotoxic activity with high selectivity for MCF-7 cells in the MTT assay.^144^ Flow cytometry analysis of 80 showed cell cycle arrest in the S phase and the accumulation of cells in the subG1 phase. The apoptotic mode of cell death induced by 80 was further confirmed by annexin-V staining assay. The wound healing assay revealed a profound impairment in the migration of MCF-7 cells presumably due to the down-regulation of Bcl-2 and Bcl-xL proteins induced by 80, as observed in the immunoblotting analysis. 80 was found to bind favorably to Bcl-2 and Bcl-xL in the docking simulation analysis, suggesting that it is a probable small molecule Bcl-2/Bcl-xL inhibitor and a potential lead for breast cancer chemotherapy with apoptotic and anti-metastatic properties.

**Fig. 79:**
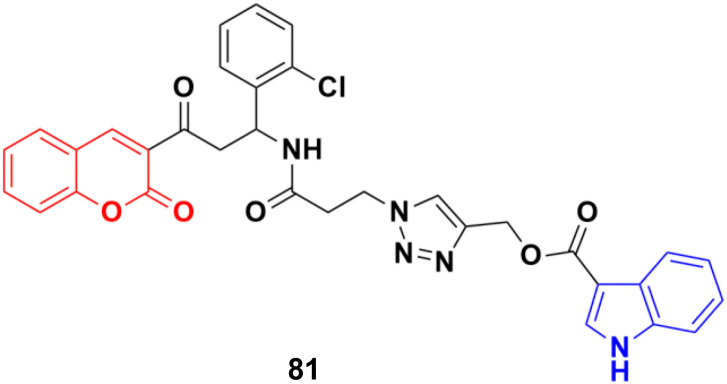
Chemical structure of coumarin–indole hybrid 81.

A new series of indole–triazole–coumarin hybrids was developed *via* copper(i)-catalyzed [3 + 2] azide–alkyne cycloaddition and showed excellent binding affinity towards CDK2 kinase with cytotoxicity against the human breast cancer cell line MCF-7.^145^ The IC_50_ value (17.5 μM) and binding affinity (−11.2 kcal mol^−1^) obtained for 81 (Fig. 79) against MCF-7 cells are promising and it can act as a lead to generate more potential anticancer moieties.

**Fig. 80:**
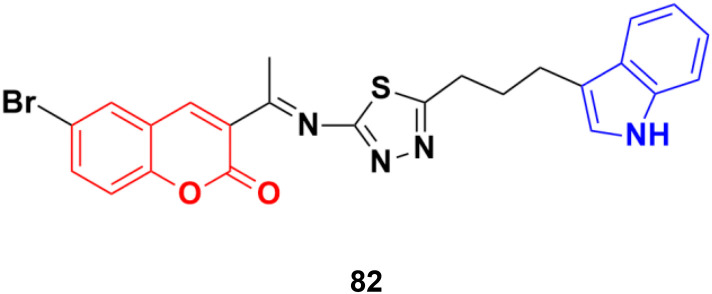
Chemical structure of coumarin–indole hybrid 82.

Another coumarin–indole hybrid, 3-(1-(5-(3-(1*H*-indol-3-yl)propyl)-1,3,4-thiadiazol-2-ylimino)ethyl)-6-bromo-2*H*-chromen-2-one (IPTBC) (82) (Fig. 80), exhibited dose-dependent cytotoxicity in breast adenocarcinoma (MCF-7) cells.^146^ This compound induced cell apoptosis through the active involvement of caspases.

**Fig. 81:**
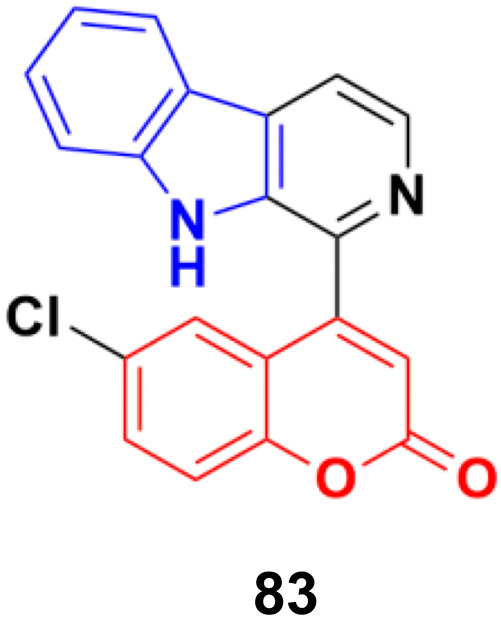
Chemical structure of coumarin-β-carboline system 83.

The coumarin-β-carboline system 83 (Fig. 81) showed antiproliferative activity against the HeLa cell line with a GI_50_ value of 23.4 μg mL^−1^.^147^*In silico* studies indicated the binding properties of 83 with the kinesin spindle protein (KSP) and tubulin protein. Gel electrophoresis studies revealed that compound 83 completely cleaved the CT-DNA.

**Fig. 82:**
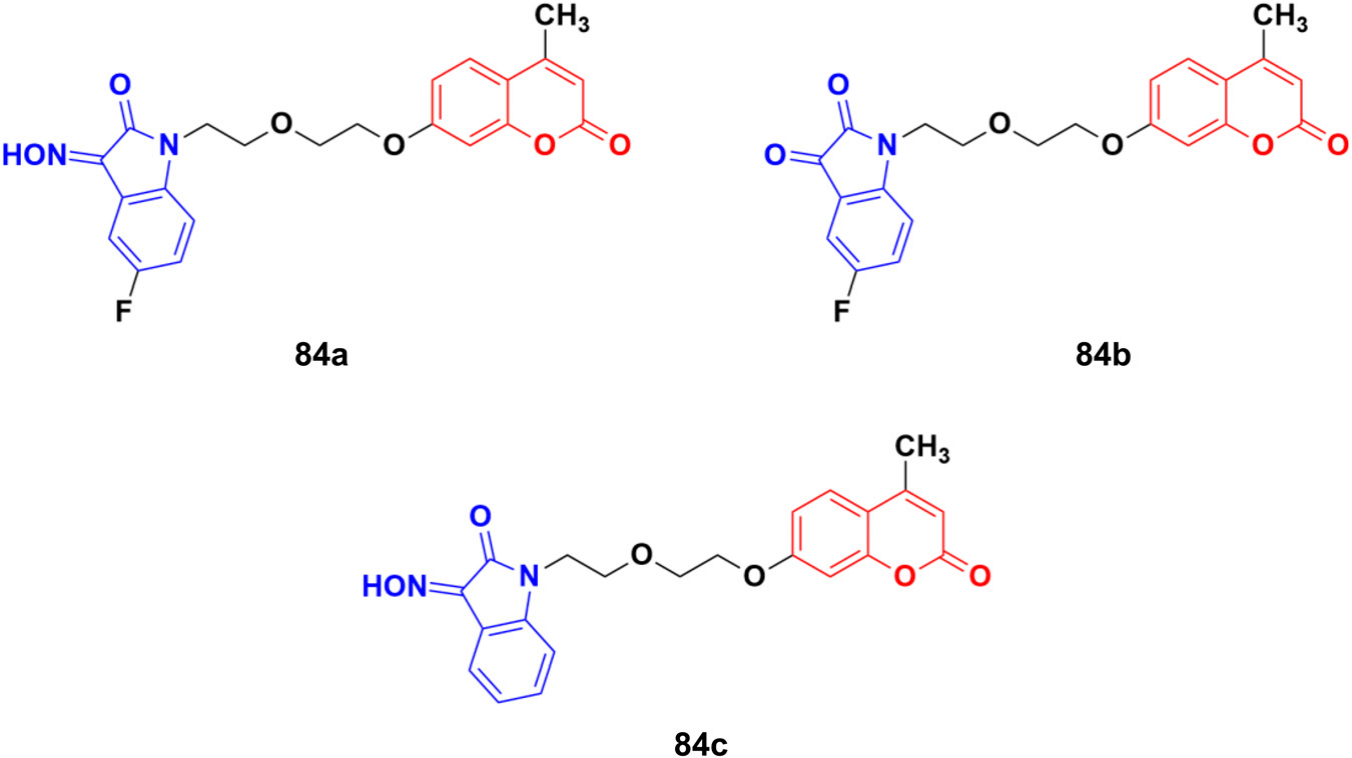
Chemical structures of coumarin–isatin hybrid 84a–c.

A novel series of twelve ethylene glycol-tethered coumarin–isatin hybrids were designed and evaluated for their *in vitro* anticancer activities against HepG2, HeLa, A549, DU145, SKOV3, and MCF-7, and drug-resistant MCF-7/DOX (doxorubicin-resistant MCF7) by SRB assay.^148^ Among them, compound 84a showed the maximum potency against HepG2, A549, DU145, MCF-7, and MCF-7/DOX with IC_50_ values of 10.28, 10.92, 20.80, 11.29, and 14.45 μM, respectively. Compounds 84b and 84c possessed the highest efficiency against the HeLa and SKOV3 cell lines with IC_50_ values of 11.54 and 18.63 μM, respectively (Fig. 82).

**Fig. 83:**
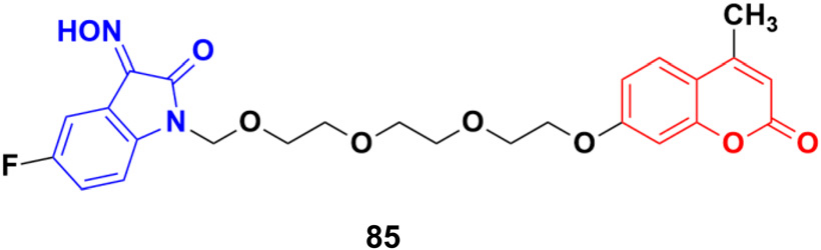
Chemical structure of coumarin–isatin hybrid 85.

Another series of twelve ethylene glycol-tethered coumarin–isatin hybrids was developed and examined for their *in vitro* cytotoxic activities against HepG2, HeLa, A549, DU145, SKOV3, and MCF-7 as well as drug-resistant MCF-7/DOX (doxorubicin-resistant MCF-7) human cancer cell lines.^149^ Most of them had very little or no anticancer activities. Compound 85 (Fig. 83) showed moderate activity against the SKOV3, and MCF-7 and MCF-7/DOX cell lines with IC_50_ values of 23.76, 11.90, and 18.85 μM, respectively.

**Fig. 84:**
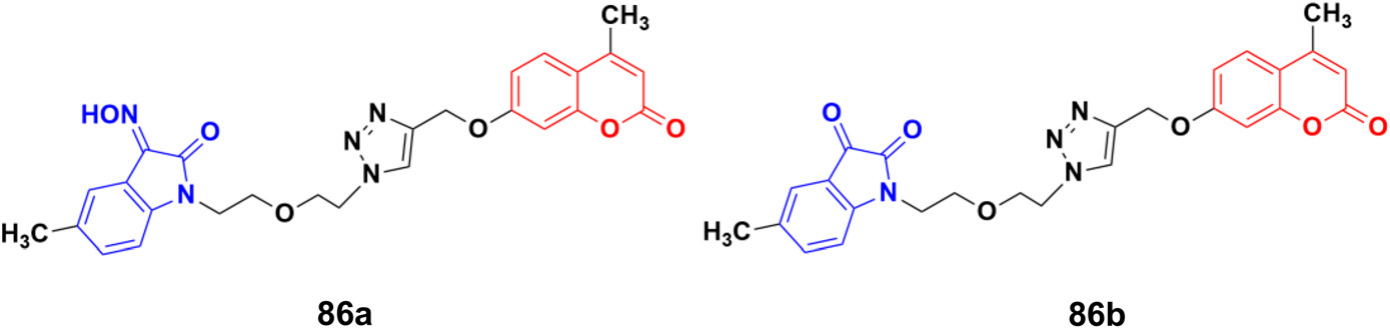
Chemical structures of coumarin–isatin hybrid 86a and b.

A series of twelve diethylene glycol-tethered isatin–1,2,3-triazole–coumarin hybrids was synthesized and investigated for their *in vitro* anticancer activities against HepG2, HeLa, A549, DU145, SKOV3, MCF-7, and MCF-7/DOX human cancer cell lines.^150^ Among them, compound 86a (Fig. 84) showed excellent potency against six of the seven cell lines (IC_50_ values of 19.89, 21.32, 18.67, 31.50, 17.96, and 15.46 μM against HepG2, HeLa, A549, DU145, SKOV3, and MCF-7/DOX cell lines, respectively). Compound 86b was most effective against the MCF-7 cell line with IC_50_ = 28.74 μM.

**Fig. 85:**
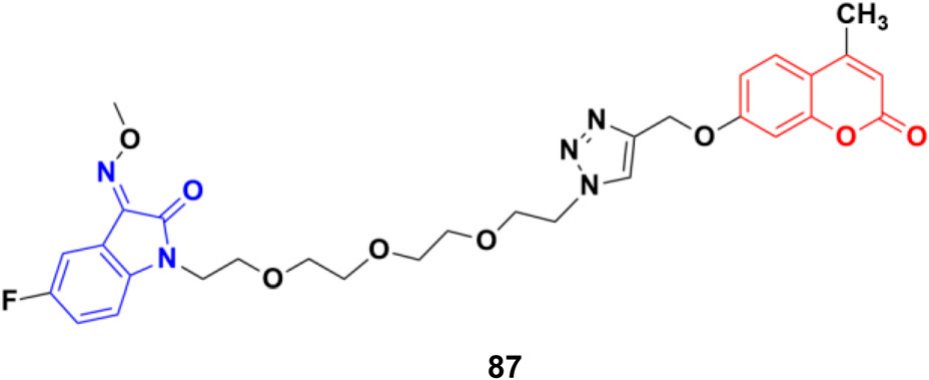
Chemical structure of coumarin–isatin hybrid 87.

A similar study was carried out on tetraethylene glycol-tethered isatin–1,2,3-triazole–coumarin hybrids.^151^ Among them, compound 87 (Fig. 85) possessed the maximum anticancer activity (IC_50_ values of 26.11, 25.49, 28.74, 33.42, 35.28, 29.25, and 20.09 against HepG2, HeLa, A549, DU145, SKOV3, MCF-7, and MCF-7/DOX, respectively).

**Fig. 86:**
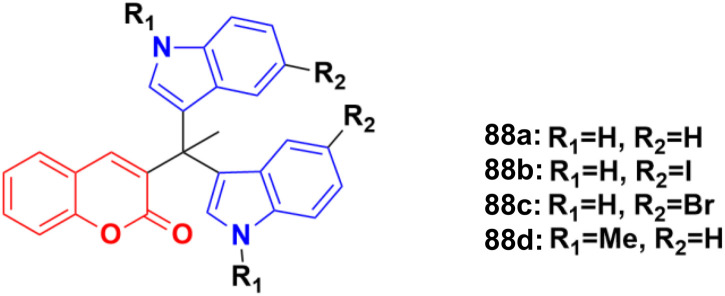
Chemical structures of coumarin–isatin hybrid 88a–d.

A sulphonic acid-functionalized nitrogen sulfur co-doped graphite (SO_3_H-NSG)-based catalyst was prepared by coating the surface of carbon with a sulphonic acid-bearing ionic liquid, which was used for the synthesis of biologically active coumarin-substituted bis(indolyl)methanes that were finally evaluated for their toxicity and anticancer properties.^152^ The cytotoxicities of compounds 88a–d (Fig. 86) were investigated against the human breast carcinoma cell line (MCF-7), osteosarcoma cell line (HOS), and normal kidney epithelial cell line (NKE) by the Amber blue reduction assay. Among them, 88a–c showed significant cytotoxicity towards the breast cancer cell line with IC_50_ of 19.75, 28.04, and 21.19 μM, respectively, whereas 88d was practically nontoxic. Similarly, 88a, b, and d showed significant cytotoxicity towards the osteosarcoma cell line with IC_50_ of 20.76, 8.75, and 12.23 μM, respectively, whereas 88c was practically nontoxic. These compounds were also evaluated for their possible cytotoxicity against normal human cell lines (NKE). However, they did not significantly affect the growth of normal human kidney cells (IC_50_ values of >50 μM).

**Fig. 87:**
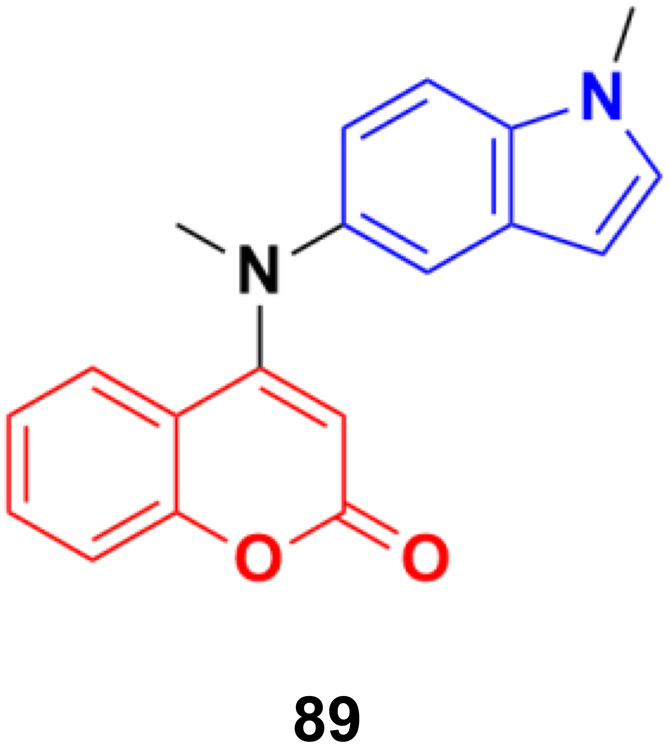
Chemical structure of coumarin–isatin hybrid 89 (MY-413).

A novel family of coumarin–indole derivatives was synthesized and evaluated as tubulin polymerization inhibitors targeting the colchicine binding site.^153^ Among them, compound MY-413 (89) (Fig. 87) displayed the most potent inhibitory activities against the gastric cancer cell line MGC-803 with an IC_50_ value of 0.011 μM. Furthermore, the IC_50_ values of compound 89 on fifteen cancer cell lines were lower than 100 nM and the IC_50_ values of 9 cancer cell lines were less than 50 nM. Compound 89 effectively inhibited tubulin polymerization (IC_50_ = 2.46 μM) by binding to the colchicine site. Screening for the inhibitory effects of the compound on 61 kinases revealed that compound MY-413 could inhibit MAPK 39 pathway-related kinases. Because of the inhibitory effects of compound MY-413 on tubulin polymerization and MAPK pathways, compound 89 induced cell apoptosis, arrested the cell cycle in the G2/M phase, induced cell proliferation inhibition and cell migration inhibition in the gastric cancer cell lines MGC-803 and HGC-27. In addition, compound MY-413 could significantly inhibit tumor growth in MGC-803 with tumor growth inhibition (TGI) rates of 70% (15 mg kg^−1^), 45% and 80% (30 mg kg^−1^) without obvious toxicity. Consistent with the *in vitro* results, compound MY-413 also inhibited the MAPK signaling pathway, and induced apoptosis and proliferation inhibition *in vivo*.

### 2.6. Coumarin–chalcone hybrids

**Fig. 88:**
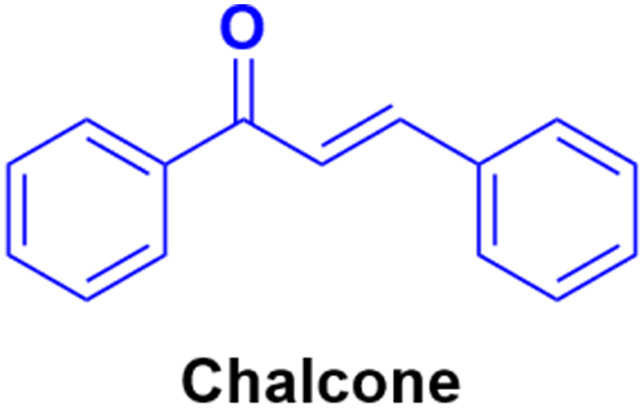
Chemical structure of chalcone.

Chalcone (Fig. 88), an α,β-unsaturated ketone, is biologically important and considered a privileged scaffold in medicinal chemistry. The beneficial effect of these substances has been studied in diabetes mellitus. Chalcone derivatives have been linked with anti-inflammatory, analgesic, antipyretic, antimutagenic, antileishmanial, antiproliferative, and antifungal effects.^154–160^ Therefore, the chalcone skeleton can be considered a useful scaffold, and its hybridization with the coumarin moiety may lead to the discovery of new potent anticancer drugs.

**Fig. 89:**
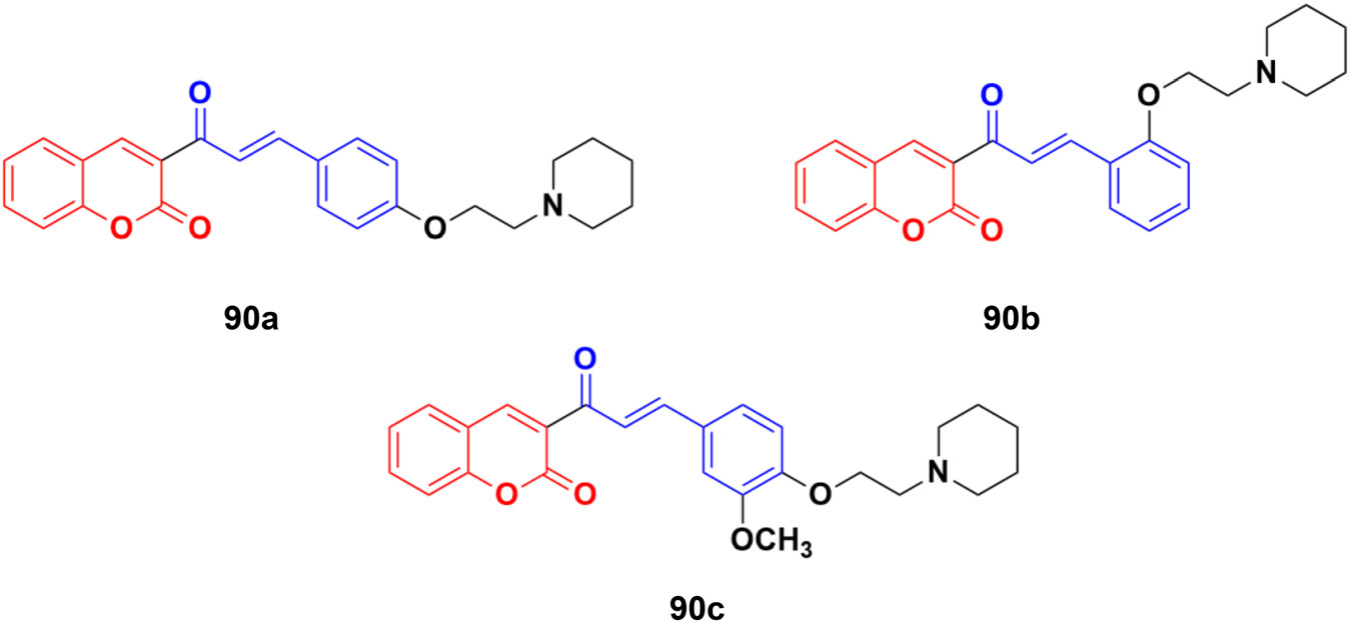
Chemical structures of coumarin–chalcone hybrid 90a–c.

A novel series of coumarin–chalcone hybrids was synthesized and evaluated for anti-proliferative activity against the estrogen receptor-positive MCF-7 and negative MDA-MB-435 breast cancer cell lines.^161^ Compounds 90a–c (Fig. 89) showed significant potency against the MCF-7 cell line with GI_50_ values of <10, 18.9, and 32.5 μg mL^−1^, respectively. Moreover, compound 90a was observed to be superior to *N*-methyl nitrosourea *in vivo* in terms of latency (5.5 weeks *vs.* 4.5 weeks) and reducing the tumor burden (3.1 *vs.* 4.45) and volume (3.3 mm^3^*vs.* 4.8 mm^3^), suggesting that it can act as a lead for the generation of more potent anticancer candidates.

**Fig. 90:**
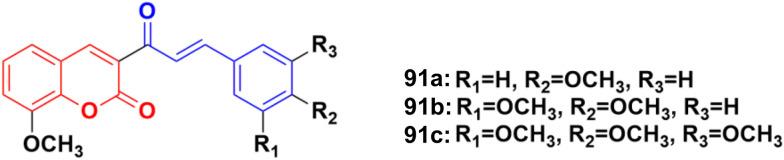
Chemical structures of coumarin–chalcone hybrid 91a–c.

Coumarin–chalcone hybrids 91a–c (Fig. 89) showed anticancer activity against the HepG2 and leukemia K562 cell lines.^162^ Compound 91a was the most potent against HepG2 with IC_50_ = 0.65 μM, while 91b showed the maximum efficiency against leukemia K562 with IC_50_ = 0.93 μM. Furthermore, cell cycle analysis of 91a showed the activation of apoptotic signals as a result of cell cycle arrest in the G2/M phase (Fig. 90).

**Fig. 91:**
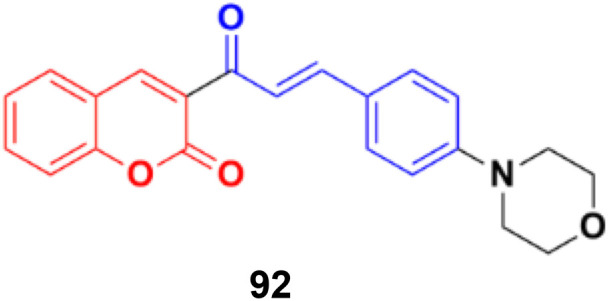
Chemical structure of coumarin–chalcone hybrid 92.

Another coumarin–chalcone hybrid (92) (Fig. 91), which is structurally similar to 90, was synthesized and its anticancer activity was investigated against the T47D breast cancer cell line and cervix cancer cell line HeLa.^163^ This compound has an IC_50_ of 0.90 μM for the T47D breast cancer cell line and 2.32 μM for the HeLa cervix cancer cell line.

**Fig. 92:**
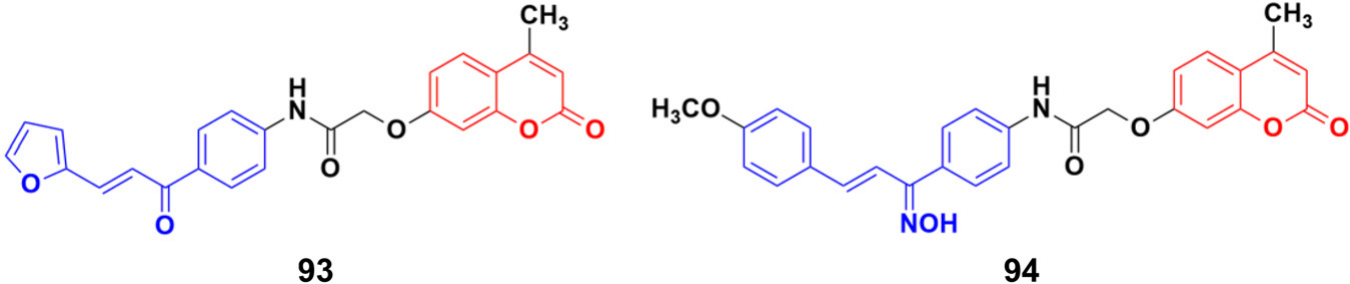
Chemical structures of coumarin–chalcone hybrid 93 and NO hybrid 94.

A novel series of coumarin–chalcone conjugates and their NO hybrids was designed and their antiproliferative properties investigated against the MCF-7 cancer cell line.^164^ The coumarin–chalcone hybrid 93 (Fig. 92) showed the maximum potency with an IC_50_ value of 9.62 μg mL^−1^. Among the NO hybrids, compound 94 was the most effective with an IC_50_ value of 20.9 μg mL^−1^.

**Fig. 93:**
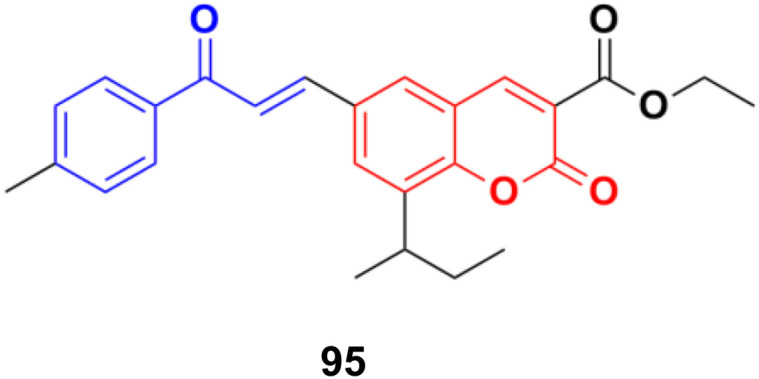
Chemical structure of coumarin–chalcone hybrid 95 (S009-131).

S009-131 (95) (Fig. 93), a coumarin–chalcone hybrid, possessed anti-proliferative and anti-tumor effects by triggering apoptosis.^165^ S009-131 caused DNA damage by potential binding to the minor groove, which led to the phosphorylation and activation of ATM and DNA-PK, but not ATR. S009-131 induced the DNA-damage-response-triggered activation of p53 through phosphorylation at its key residues.

**Fig. 94:**
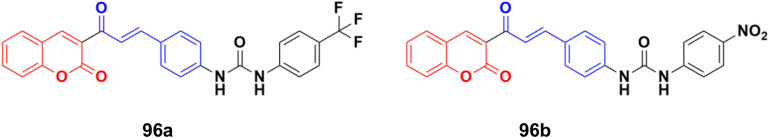
Chemical structures of coumarin–chalcone hybrid 96a and b.

A series of novel coumarin–chalcone derivatives containing a urea moiety was developed and screened for their *in vitro* antiproliferative activities against cancer cell lines (H4IIE and HepG2).^166^ In addition, the compounds were also tested on a normal cell line (CHO). Among them, compound 96a (Fig. 94) exhibited the maximum potency against H4IIE compared to sorafenib with an IC_50_ value of 1.62 ± 0.57 μM. Compound 96b also showed better inhibition against HepG2 than sorafenib with an IC_50_ value of 2.326 ± 0.23 μM. Particularly, 96a induced H4IIE apoptosis and arrested cell cycle in the S phase.

**Fig. 95:**
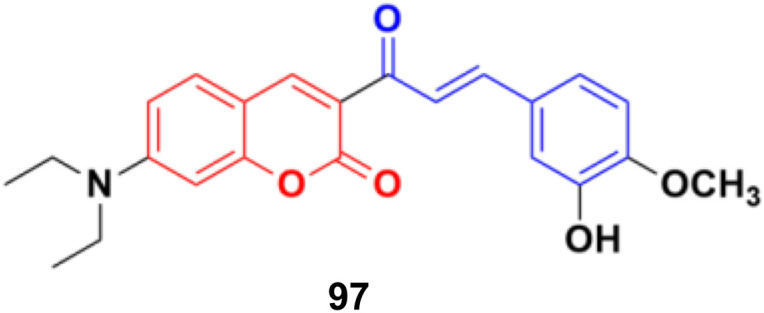
Chemical structure of coumarin–chalcone hybrid 97.

A series of coumarin–chalcone hybrids was synthesized as selenoprotein thioredoxin reductase (TrxRs) inhibitors.^167^ Most of them exhibited enhanced anticancer activity compared to xanthohumol (Xn). Among them, compound 97 (Fig. 95) (IC_50_ = 3.6 μM), a fluorescence agent, down-regulated the expression of TrxR and remarkably induced ROS accumulation to activate the mitochondrial apoptosis pathway. Furthermore, it inhibited cancer cell metastasis and abolished the colony formation ability of cancer cells.

**Fig. 96:**
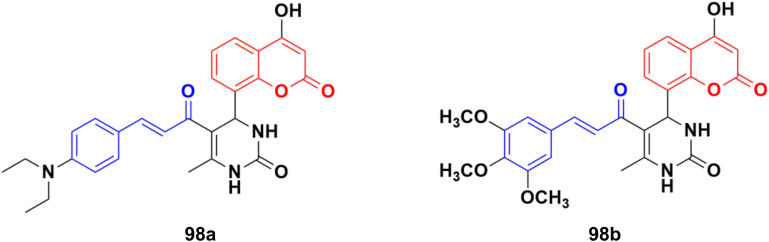
Chemical structures of coumarin–chalcone hybrid 98a and b.

A new series of thirteen coumarin-yl–chalcone derivatives was synthesized and *in silico* studies were performed to predict the anticancer activity of the compounds against Src, Alb tyrosine kinase, and homology model protein (PDB ID: 4csv).^168^ Derivatives 98a and b (Fig. 96) showed moderate binding energies. The *in vitro* cytotoxic activity was analyzed for these two compounds against three human cell lines, including A549, Jurkat, and MCF-7. The results indicated that the hybrids displayed significant anticancer activity but are less cytotoxic than the standard imatinib.

**Fig. 97:**
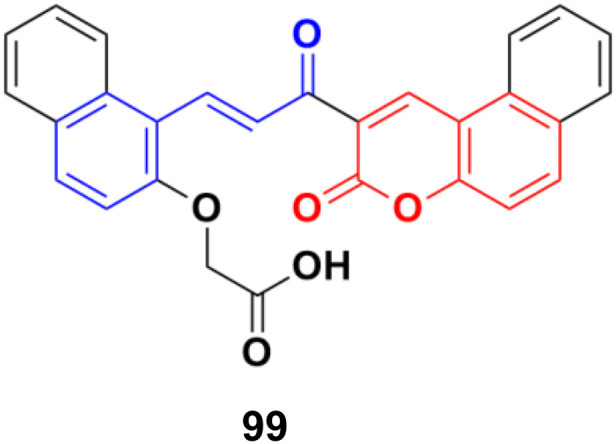
Chemical structure of coumarin–chalcone hybrid 99.

A water-soluble chemo-sensor (99) (Fig. 97) consisting of a chalcone–coumarin framework, which displayed excellent selectivity and sensitivity towards Al^3+^ ions, showed significant anticancer activity against the MCF-7 cancer cell line with an IC_50_ value of 15.38 μM.^169^

### 2.7. Coumarin-imine hybrids

**Fig. 98:**
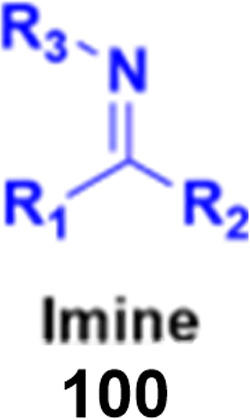
Chemical structure of imine.

Imine (Fig. 98), consisting of a carbon–nitrogen double bond, is regarded as an important pharmacophore and has been used in the synthesis of many drugs.^170^ It can bind with the various active sites of living organisms through non-covalent interaction, which can be used to design new drugs effectively.

**Fig. 99:**
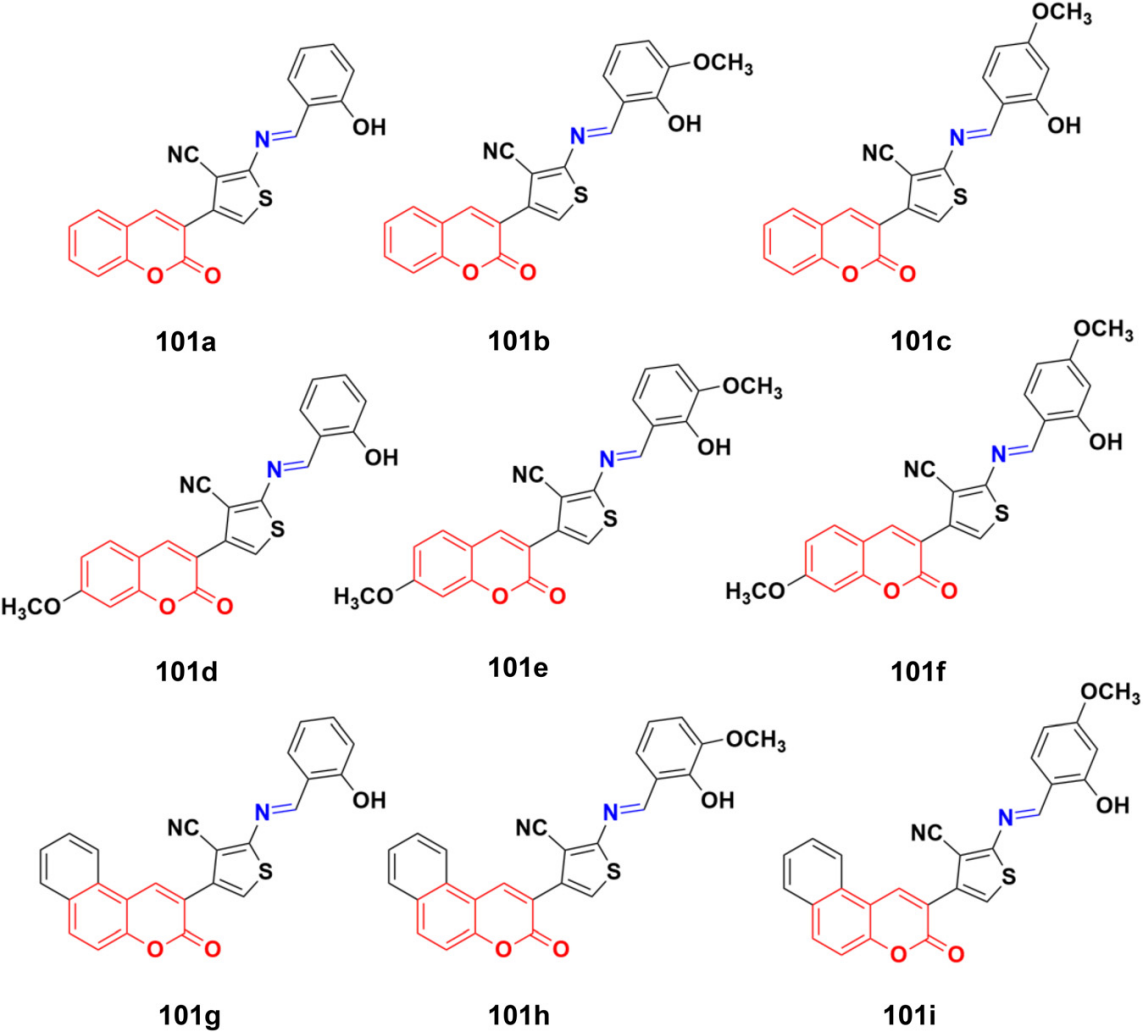
Chemical structures of coumarin-imine hybrids 101a–i.

A similar series of nine coumarin-imine hybrids (101a–i) (Fig. 99) was synthesized and their antiproliferative profile evaluated against fibroblast cell lines and A549 cancer cell line.^171^ The percentage of viable cells was determined at different concentrations in the range of 12.5 to 200 μg mL^−1^. In terms of the WST-1 results, the concentrations of the compounds did not have a prominent effect on cell mortality in the cell line. For the fibroblast cells, the results were significant for only 101a and 101b. However, for the other samples (101c–i), increasing the concentrations of the compounds caused an increase in cell death.

**Fig. 100:**
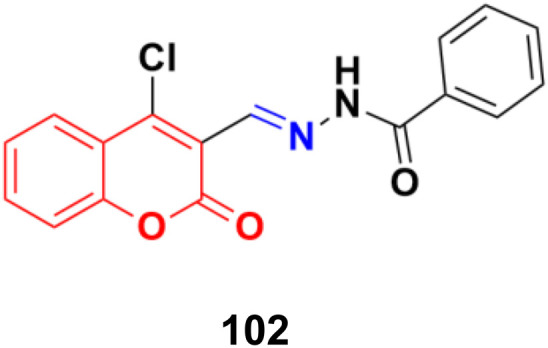
Chemical structure of coumarin-imine hybrid 102.

A series of coumarin–hydrazone hybrids was designed and evaluated for their anticancer activities against four cancer cell lines.^172^ Among them, compound 102 (Fig. 100) showed the most potency with IC_50_ = 2.9 ± 0.4, 5.3 ± 1.1, 7.2 ± 0.9, and 9.1 ± 1.2 μM against the HL-60, KE-37, K-562, and MDA-MB-231 cell lines, respectively.

### 2.8. Coumarin-sulfonamide/sulfamate/sulfonate hybrids

**Fig. 101:**
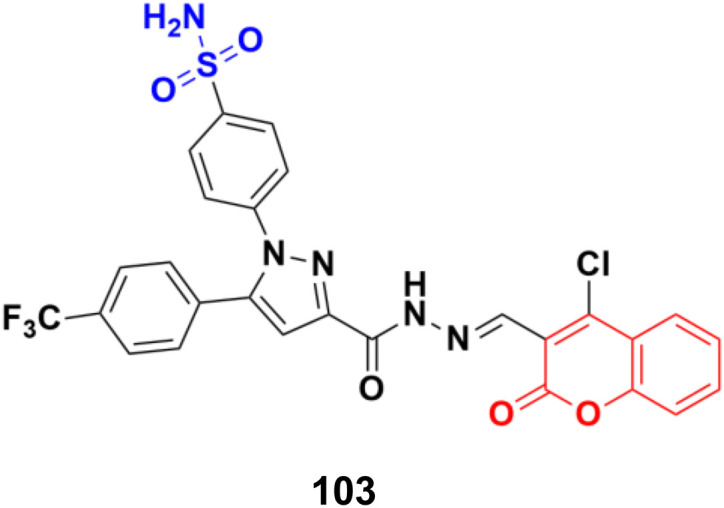
Chemical structure of coumarin-sulfonamide hybrid 103.

A novel series of coumarin sulfonamide derivatives was designed to improve the biological activities of COX-2 inhibition and anticancer.^173^ Among the synthesized compounds, 103 (Fig. 101) possessed the most powerful selective inhibitory and antiproliferative activity (IC_50_ = 0.09 μM for COX-2, 48.20 μM for COX-1, and 0.36 μM against HeLa cells), which is comparable to the control positive compound celecoxib (0.31 μM, 43.37 μM, and 7.79 μM). Compound 103 effectively induced HeLa cell apoptosis in a dose- and time-dependent manner. Moreover, it could significantly suppress cancer cell adhesion, migration, and invasion. The docking simulation results further indicated that 103 could bind well to the COX-2 active site and guided the reasonable design of a selective COX-2 inhibitor with an anticancer nature shortly.

**Fig. 102:**
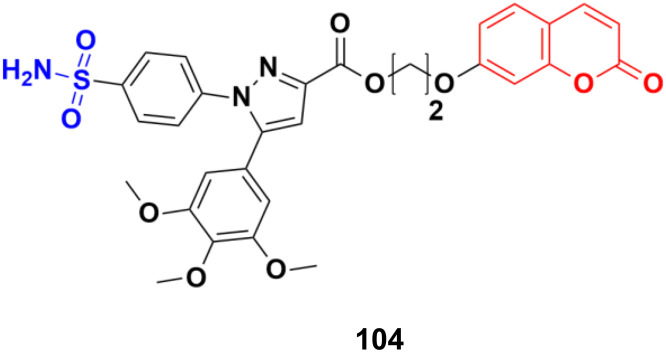
Chemical structure of coumarin-sulfonamide hybrid 104.

Coumarin-sulfonamide hybrid 104 (Fig. 102) was designed to obtain novel dual inhibitors of COX-2 and 5-LOX.^174^ Compound 104 (IC_50_ = 0.23 ± 0.16 μM for COX-2, 0.87 ± 0.07 μM for 5-LOX, and 4.48 ± 0.57 μM against A549) showed preliminary superiority compared with the positive controls celecoxib (IC_50_ = 0.41 ± 0.28 μM for COX-2 and 7.68 ± 0.55 μM against A549) and zileuton (IC_50_ = 1.35 ± 0.24 μM for 5-LOX). Further investigation confirmed that 104 could induce apoptosis and cell cycle arrest at the G2 phase in a dose-dependent manner in human non-small cell lung cancer A549 cells.

**Fig. 103:**
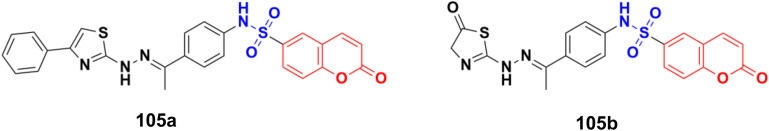
Chemical structures of coumarin-sulfonamide hybrid 105a and b.

Different novel sets of coumarin-6-sulfonamide derivatives were synthesized and their growth inhibitory activity evaluated *in vitro* towards the proliferation of three cancer cell lines, *i.e.*, HepG2, MCF-7, and Caco-2.^175^ Compounds 105a and 105b (Fig. 103) emerged as the most active members against HepG2 cells (IC_50_ = 3.48 ± 0.28 and 5.03 ± 0.39 mM, respectively). These compounds could induce apoptosis in HepG2 cells, as demonstrated by the upregulation of Bax and downregulation of Bcl-2, besides boosting the caspase-3 levels. Besides, compound 105a induced a significant increase in the percentage of cells at pre-G1 by 6.4-fold, with concurrent significant arrest in the G2-M phase by 5.4-fold compared to the control. Also, 105a displayed a significant increase in the percentage of annexin V-FITC-positive apoptotic cells from 1.75% to 13.76%.

**Fig. 104:**
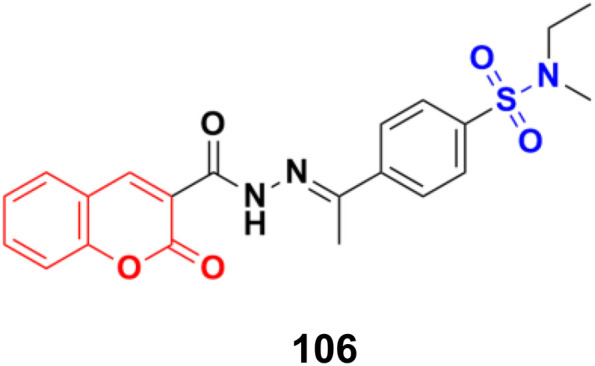
Chemical structure of coumarin-sulfonamide hybrid 106.

Coumarin-sulfonamide hybrid 106 (Fig. 104) showed excellent antiproliferative properties against the MCF-7 cancer cell line with IC_50_ = 2.53 μM, together with the selective index (SI) of 59.26.^176^

**Fig. 105:**
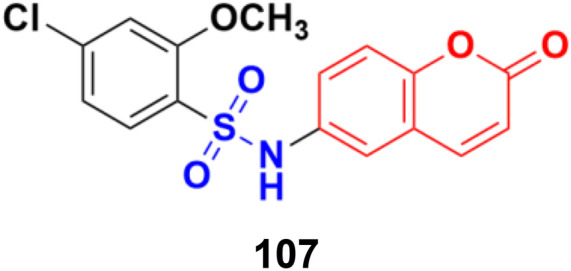
Chemical structure of coumarin-sulfonamide hybrid 107.

Coumarin-sulfonamide hybrid 107 (Fig. 105), synthesized as a new chemotype of BRD4 inhibitors, possessed excellent cytotoxic ability against A549 (IC_50_ = 4.63 μM), HepG2 (IC_50_ = 4.75 μM), PANC-1 (IC_50_ = 7.02 μM), and SGC-7901 (IC_50_ = 6.39 μM) cell lines.^177^ Moreover, compound 107 exhibited potent BRD4 binding affinity and cell proliferation inhibitory activity, and especially displayed a favorable PK profile with high oral bioavailability (*F* = 49.38%) and metabolic stability (*T*_1/2_ = 4.2 h), meaningfully making it a promising lead compound for further drug development.

**Fig. 106:**
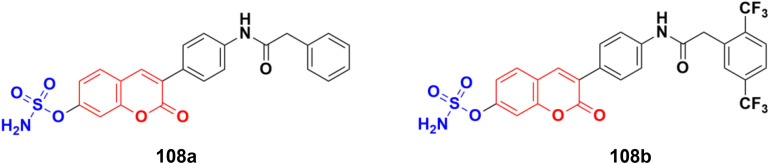
Chemical structures of coumarin-sulfamate hybrid 108a and b.

A novel series of coumarin-sulfamate hybrids was prepared as potential STS inhibitors.^178^ The inhibitory effects of the synthesized compounds were tested on STS isolated from the human placenta and against estrogen receptor-(ER)-positive MCF-7 and T47D cells, as well as ER-negative MDA-MB-231 and SkBr3 cancer cell lines. Among the synthesized compounds, 108a and 108b (Fig. 106) showed the highest inhibitory effect in enzymatic STS assays, both with IC_50_ values of 0.18 μM. Compound 108b exhibited the highest potency against the MCF-7 and T47D cell lines (15.9 μM and 8.7 μM, respectively).

**Fig. 107:**
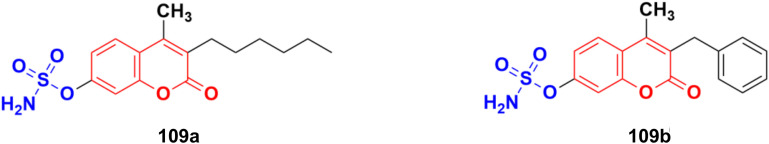
Chemical structures of coumarin-sulfamate hybrid 109a and b.

Potent bicyclic nonsteroidal sulfamate-based active-site-directed inhibitors of the enzyme steroid sulfatase (STS), an emerging target in the treatment of post-menopausal hormone-dependent diseases, including breast cancer, were designed.^179^ The compounds were examined for STS inhibition in intact MCF-7 breast cancer cells and in placental microsomes. 3-Hexyl-4-methylcoumarin-7-*O*-sulfamate 109a and 3-benzyl-4-methylcoumarin-7-*O*-sulfamate 109b (Fig. 107) were particularly effective inhibitors with IC_50_ values of 0.68 and 1 nM in intact MCF-7 cells and 8 and 32 nM for placental microsomal STS, respectively. They were docked in the STS active site for comparison with estrone 3-*O*-sulfamate and irosustat, showing their sulfamate group close to the catalytic hydrated formylglycine residue and their pendant group lying between the hydrophobic side-chains of L103, F178, and F488.

**Fig. 108:**
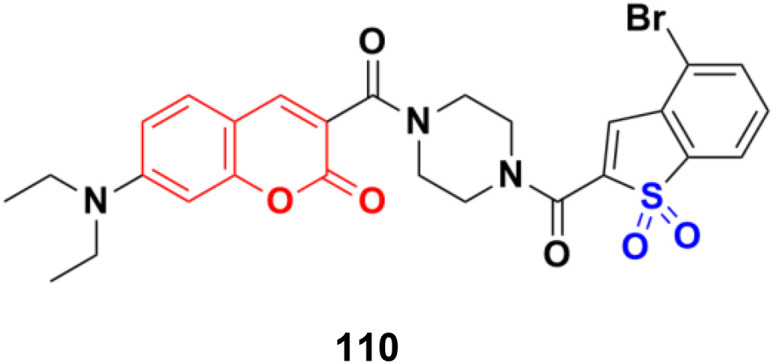
Chemical structure of coumarin–sulfonate hybrid 110.

A series of STAT3 inhibitors was developed and their anti-proliferative activity against four cancer cells investigated.^180^ Among them, compound 110 (Fig. 108) was the most potent with IC_50_ = 1.43 ± 0.30, 1.89 ± 0.42, 2.88 ± 0.69, and 3.33 ± 0.23 μM against the MDA-MB-231, HCT-116, HepG2, and MCF-7 cancer cell lines, respectively. STAT3 phosphorylation was inhibited by compound 110 at both Tyr705 and Ser727 residues. Compound 110 inhibited STAT3 phosphorylation, whereas it did not influence the phosphorylation levels of STAT1, 26 JAK2, Src, and Erk1/2, indicating its good selectivity. Moreover, compound 110 down-regulated the expression of the STAT3-target genes Bcl-2 and cyclin D1, increased ROS production, and remarkably reduced the mitochondrial membrane potential to induce the mitochondrial apoptotic pathway. It also suppressed breast cancer 4T1-implanted tumor growth *in vivo*.

**Fig. 109:**
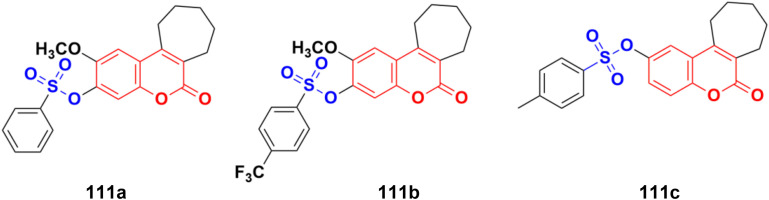
Chemical structure of coumarin–sulfonate hybrid 111a–c.

Fourteen new cycloalkane-fused tricyclic coumarin sulfonate derivatives were developed and evaluated for *in vitro* anticancer activity against the NCI-57 cancer cell line panel of nine different cancer types.^181^ Among the compounds, 111a, 111b, and 111c (Fig. 109) showed the highest activities. Compound 111b exerted the highest percentage of growth inhibition (91.91%) against the SNB-75 CNS cancer cell line at 10 μM concentration and was more active than carmustine against this cell line. Compound 111a also showed strong activity against HT29 colon, ACHN renal, and PC-3 prostate cancer cell lines. Furthermore, compound 111c was selective toward the HT29 colon cancer cell line.

**Fig. 110:**
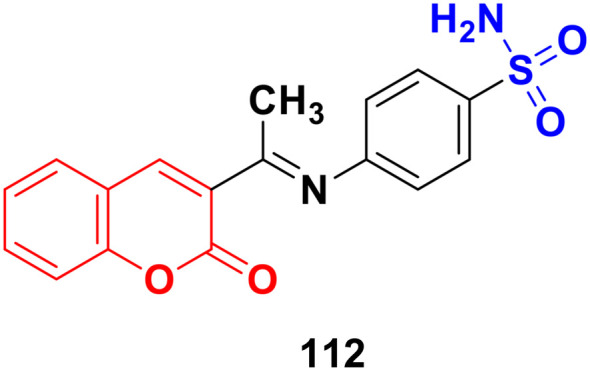
Chemical structure of coumarin-sulfonamide hybrid 112.

A series of coumarin-sulfonamide hybrids was synthesized by the condensation reaction of appropriate *N*-heteroaryl-4-amino benzenesulfonamide with derivatives of 3-acetyl coumarin and their antiproliferative property was screened against the MDA-MB-231, MIA PaCa-2, and H357 cancer cell lines.^182^ Among them, compound 112 (Fig. 110) demonstrated significant activity against the MDA-MB-231 cell line (IC_50_ = 7.78 ± 3.78 μM) and H357 cell line (IC_50_ = 8.68 ± 1.10 μM) after 72 h.

### 2.9. Dihydroxycoumarins

Dihydroxycoumarins are an important class of benzopyrones with different pharmacological properties such as antimicrobial and anticancer.

**Fig. 111:**
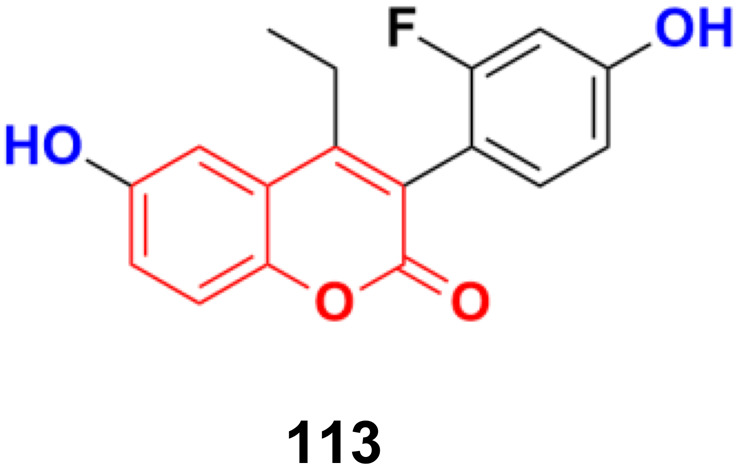
Chemical structure of dihydroxycoumarin 113.

A novel series of coumarin-based nonsteroidal-type fluorescence ligands for drug–target binding imaging was designed and developed.^183^ Among the synthesized compounds, 113 (Fig. 111) showed potent antiproliferative activity against the MCF-7 cancer cell line with IC_50_ = 16.1 ± 0.7 μM and against MDA-MB-453 with IC_50_ = 8.03 ± 0.6 μM. Furthermore, compound 113 could cross the cell membrane, localize, and image drug–target interaction in real time without cell washing.

**Fig. 112:**
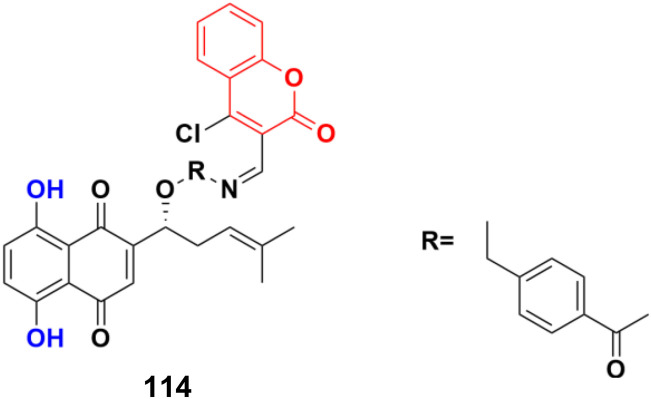
Chemical structure of dihydroxycoumarin 114.

A shikonin derivative, PMMB232 (114) (Fig. 112), showed antiproliferation activity with an IC_50_ value of 3.25 ± 0.35 μM.^184^ Further, the treatment of HeLa cells with a variety of concentrations of 114 resulted in a dose-dependent event marked by apoptosis. To identify the detailed role and mechanism of PMMB232 in the progression of human cervical cancer, the expression of HIF-1α and E-cadherin in HeLa cells was detected. The results revealed that expression of HIF-1α was downregulated, while E-cadherin protein was upregulated. Meanwhile, glycolysis-related protein PDK1 decreased in the HeLa cells. Conversely, the expression of PDH-E1α was upregulated. The docking simulation results further indicated that PMMB232 can be well bound to HIF-1α.

**Fig. 113:**
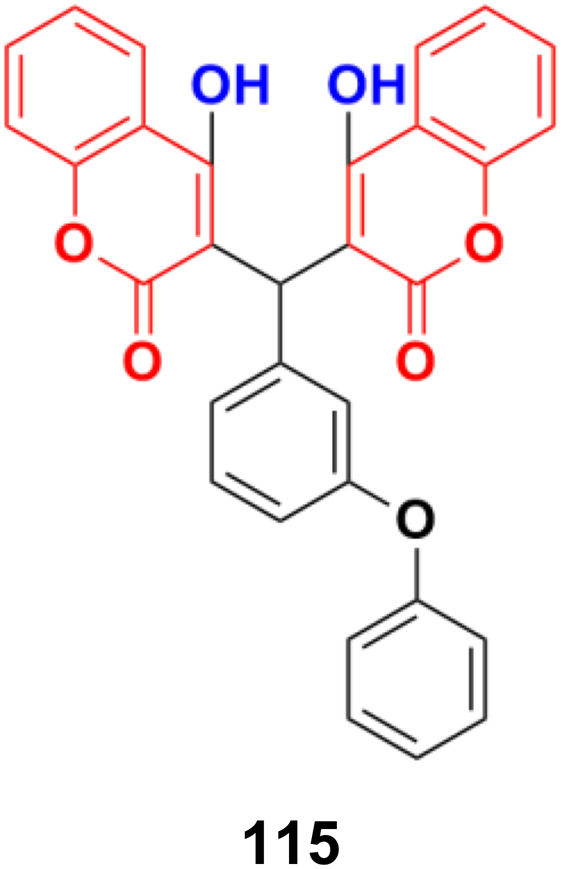
Chemical structure of dihydroxycoumarin 115.

Six 4-hydroxycoumarin derivatives were synthesized and their cytotoxic activities were investigated against four cancer cell lines (SMMC-7721, Bel-7402, MHCC97, and Hep3B).^185^ Compound 115 (Fig. 113) showed the maximum potency with IC_50_ values = 6 ± 1.4, 8 ± 2.0, 7 ± 1.7, 9 ± 2.0 μM against SMMC-7721, Bel-7402, MHCC97 and Hep3B, respectively.

**Fig. 114:**
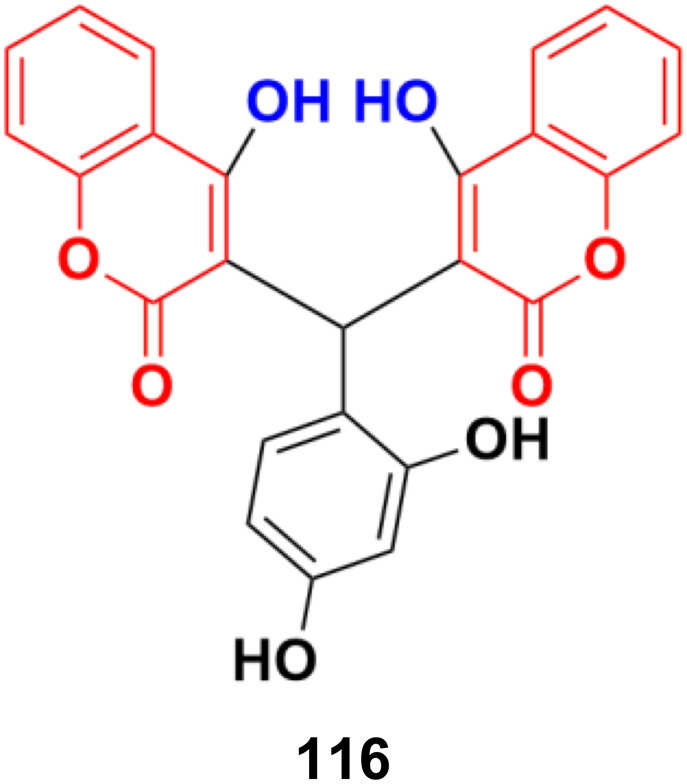
Chemical structure of dihydroxycoumarin 116.

A series of nine coumarin derivatives was synthesized and their anticancer activity tested against four human breast cancer cells *in vitro* using the MTT assay.^186^ Among them, compound 116 (Fig. 114) showed the maximum potency with IC_50_ = 25.3 ± 2.3, 15.2 ± 2.4, 25.7 ± 2.2, and 20.2 ± 3.0 μM against the MDA-MB-231, MDA-MB-468, Hst578, and HCC1937 cell lines, respectively.

**Fig. 115:**
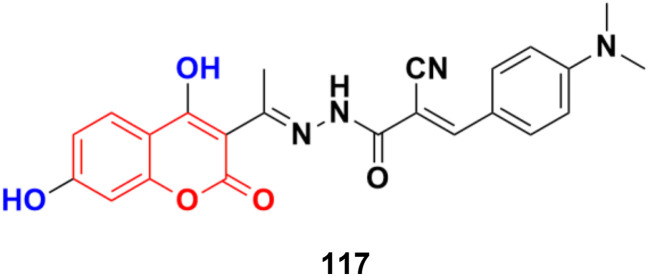
Chemical structure of dihydroxycoumarin 117.

A series of novel 4,7-dihydroxycoumarin-based acryloylcyanohydrazone derivatives was synthesized and evaluated for their antiproliferative activity against four different cancer cell lines (A549, HeLa, SKNSH, and MCF7).^187^ Compound, 117 (Fig. 115) was the most active with IC_50_ values of 4.31 ± 0.04, 5.14 ± 0.16, 6.09 ± 0.32, and 3.42 ± 0.52 μM against A549, HeLa, SKNSH, and MCF7, respectively. Further results revealed that compound 117 induced cell cycle arrest at the G2/M phase and inhibited tubulin polymerization. The experimental data from the tubulin polymerization inhibition assay was validated by the molecular docking technique and the results exhibited strong hydrogen bonding interactions with amino acids (ASN-101, TYR-224, ASN-228, and LYS-254) of tubulin.

### 2.10. Anilinocoumarin hybrids

In recent years, it has been observed that when the aniline moiety is tagged with a coumarin scaffold, the anticancer property of the hybrid increases. Thus, designing anilinocoumarin hybrids is an important strategy for the synthesis of new anticancer drugs.

**Fig. 116:**
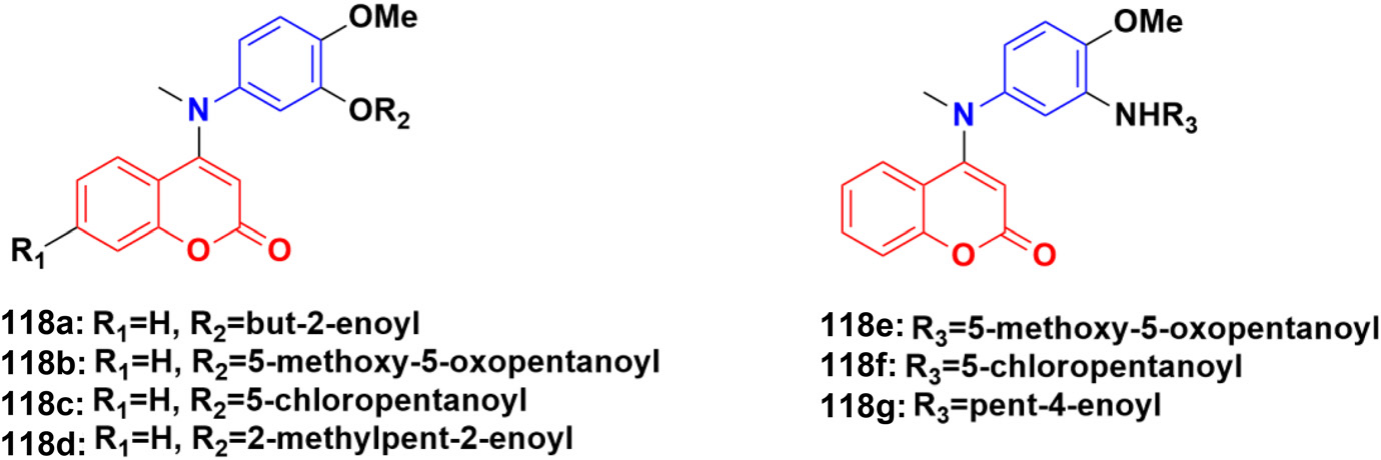
Chemical structures of anilinocoumarin 118a–g.

A series of novel 4-substituted coumarin derivatives was synthesized and their antiproliferative activity toward a panel of tumor cell lines was investigated.^188^ Among them, compounds 118a–g (Fig. 116) showed potent antiproliferative ability. 118f was the most potent (IC_50_ values = 7–47 nM) and retained full activity in multidrug-resistant cancer cells. 118f caused G2/M phase arrest and interacted with the colchicine-binding site in tubulin, reducing the cell migration and disrupting capillary-like tube formation in HUVEC cells. Importantly, compound 118f significantly and dose-dependently reduced tumor growth in four xenograft models including paclitaxel-sensitive and resistant ovarian tumors (A2780s and A2780/T) and adriamycin-sensitive and resistant breast tumors (MCF-7 and MCF-7/ADR, respectively).

**Fig. 117:**
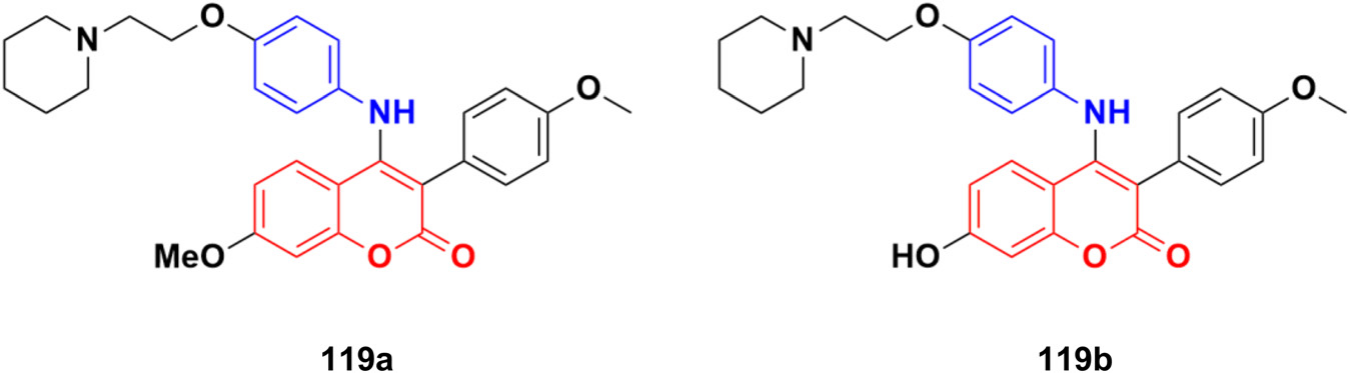
Chemical structures of anilinocoumarin 119a and b.

Eighteen selective ERα modulators (SERMs) were designed and their biological activity investigated against MCF-7 and Ishikawa cell lines.^189^ The piperidyl-substituted compounds such as 119a and 119b (Fig. 117) demonstrated strong ERα binding affinities and excellent anti-proliferative activities. Compound 119b displayed the most potent ERα binding affinity with an RBA value of 2.83%, while 119a exhibited the best anti-proliferative activity against MCF-7 cells with an IC_50_ value of 4.52 ± 2.47 μM.

**Fig. 118:**
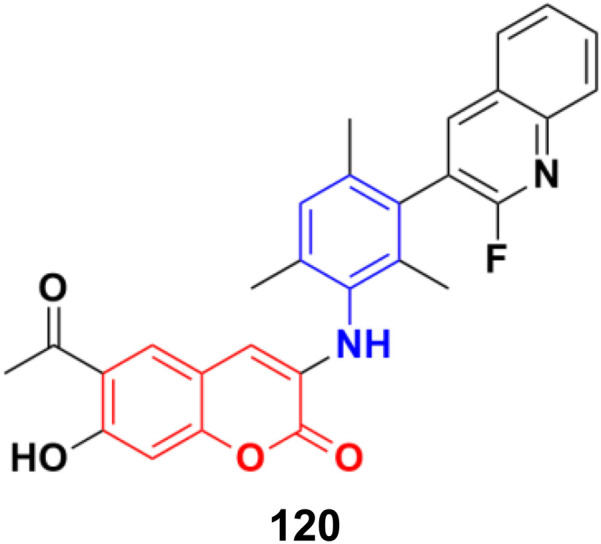
Chemical structure of anilinocoumarin 120.

A novel series of 3-(*N*-substituted)aminocoumarins was developed rapidly and efficiently and their antiproliferative activity examined against human cancer cell lines.^190^ Compound 120 (Fig. 118) showed excellent anticancer activity against MT-4, MDA-MB-231, and MCF-7 cancer cell lines with GI_50_ values of 12.6 ± 0.9, 11.8 ± 1.1 and 10.5 ± 1.2 μM, respectively.

**Fig. 119:**
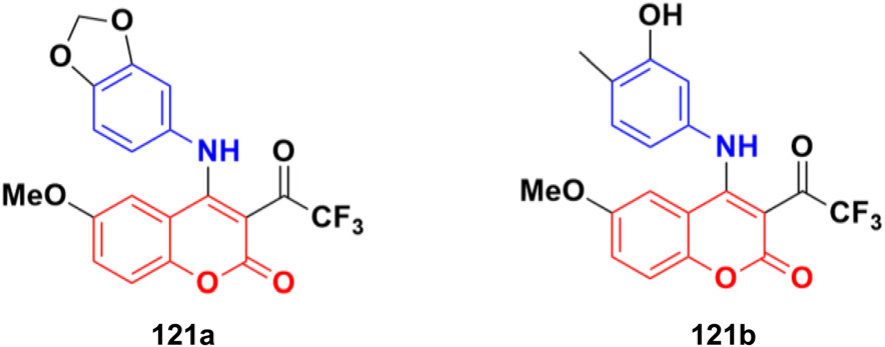
Chemical structures of anilinocoumarin 121a and b.

Various 3-substituted 4-anilino-coumarin derivatives were designed and synthesized and their *in vitro* cytotoxicity screening performed against MCF-7, HepG2, HCT116, and Panc-1 cancer cell lines by the MTT assay.^191^ Most of the synthesized compounds exhibited comparable anti-proliferative activity to the positive control 5-fluorouracil against the four tested cancer cell lines. Among the different substituents at the C-3 position the of coumarin scaffold, the 3-trifluoroacetyl group showed the most promising results. Especially, compounds 121a (Fig. 119) (IC_50_ = 16.57, 5.45, 4.42 and 5.16 μM) and 121b (IC_50_ = 20.14, 6.71, 4.62 and 5.62 μM) showed excellent anti-proliferative activities on MCF-7, HepG2, HCT116 and Panc-1 cell lines, respectively. In addition, cell cycle analysis and apoptosis activation revealed that 121a induced G2/M phase arrest and apoptosis in MCF-7 cells in a dose-dependent manner. The low toxicity of compounds 121a and 121b was observed against human umbilical vein endothelial cells (HUVECs), suggesting their acceptable safety profiles in normal cells. Furthermore, the results of *in silico* ADME studies indicated that both 121a and 121b exhibited good pharmacokinetic properties.

**Fig. 120:**
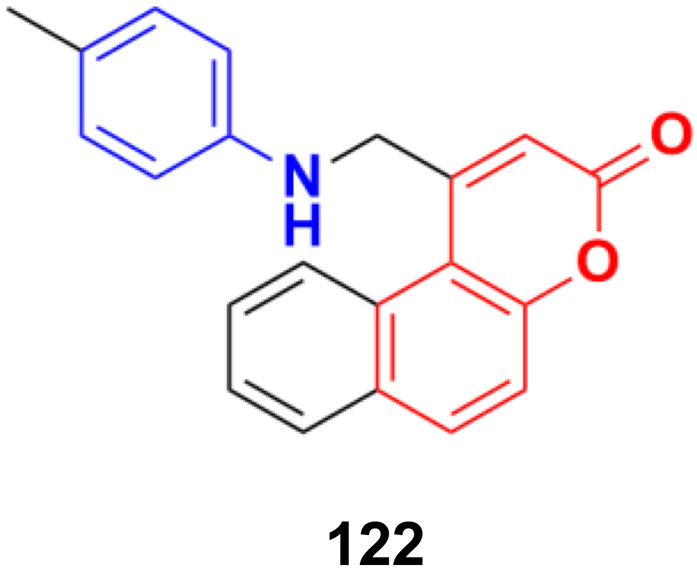
Chemical structure of anilinocoumarin 122.

A series of substituted aminomethyl benzocoumarin derivatives was synthesized and tested for their anticancer activity against the A549, MCF7, and A375 cancer cell lines.^192^ Among them, the anilinocoumarin compound 122 (Fig. 120) showed excellent growth inhibitory activity against the A549, MCF7, and A375 cancer cell lines with IC_50_ values of 4.29 μM, 5.17 μM, and 9.02 μM, respectively. Compound 122 was also found to be quite promising at very low concentrations as an anticancer agent against the MCF7 and A549 cell lines.

**Fig. 121:**
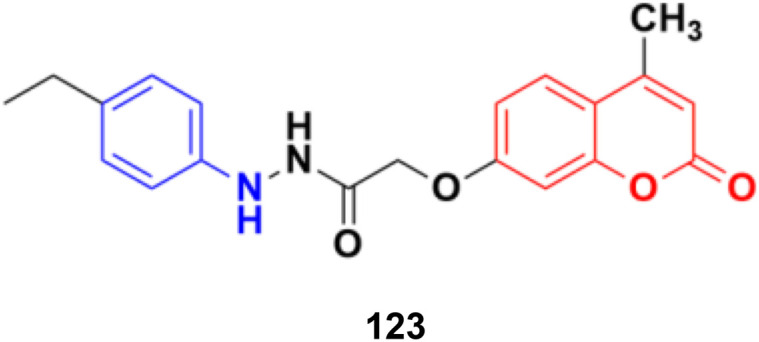
Chemical structure of anilinocoumarin 123.

Twenty-five coumarin-based derivatives were developed and investigated for their *in vitro* anticancer activity against the MCF-7 breast and PC-3 prostate cancer cell lines and further assessed for their *in vitro* VEGFR-2 kinase inhibitory activity.^193^ Among them, compound 123 (Fig. 121) (IC_50_ = 1.24 μM) exhibited exceptional activities superior to the positive control staurosporine (IC_50_ = 8.81 μM). Further study revealed that compound 123 was capable of inducing preG1 apoptosis, cell growth arrest at the G2/M phase, and activating caspase-9. A molecular docking study suggested that the most active anti-VEGFR-2 derivative 123 demonstrated the ability to interact with the key amino acids in the target VEGFR-2 kinase binding site.

**Fig. 122:**
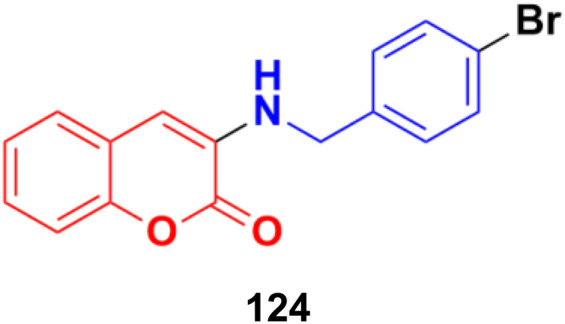
Chemical structure of anilinocoumarin 124.

A series of substituted coumarins was synthesized directly from coumarins and azides in the presence of Pr(OTf)_3_ without any additives or ligands and the cytotoxic activity of the compounds was tested against the MGC-803, A549, and NCI-H460 cancer cell lines.^194^ Compound 124 (Fig. 122) showed maximum potency having IC_50_ values of 10.19 ± 1.12, 8.75 ± 1.10, and 9.25 ± 1.28 μM against the MGC-803, A549, and NCI-H460 cell lines, respectively.

### 2.11. Coumarin-metal hybrids

Metal complexes play a vital role in various biochemical phenomena. The presence of metal ions accelerates the drug action, providing better drug delivery, and thus has become important in recent years for better pharmacological effect and enhanced efficiency of a particular metal-based drug.

#### 2.11.1. Coumarin–ferrocene hybrids

**Fig. 123:**
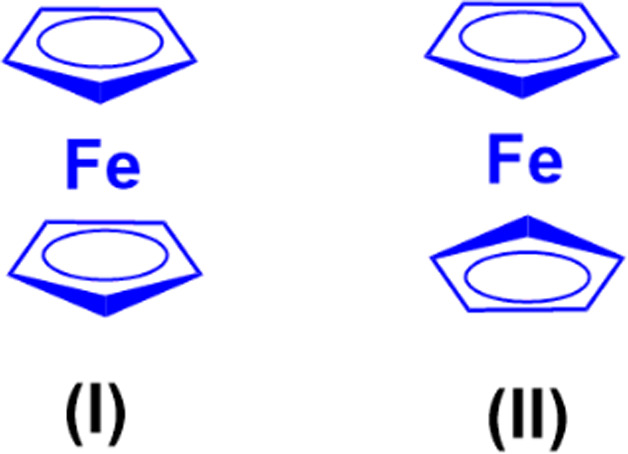
Chemical structure of ferrocene.

In recent years, organometallic compounds, especially ferrocene (Fig. 123), have emerged as important candidates for the preparation of anticancer drugs. Ferrocene derivatives show excellent structural and mechanistic diversity, inherent stability towards air, heat, and light, low toxicity, low cost, reversible redox, ligand exchange, and catalytic properties.^195^ Thus, the strategy of the hybridization of the ferrocene moiety with the coumarin scaffold may be fruitful for better pharmacological and pharmacokinetic effects.

**Fig. 124:**
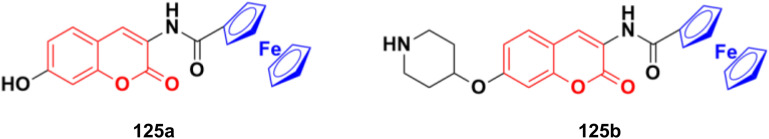
Chemical structures of coumarin–ferrocene hybrid 125a and b.

A series of tailored novobiocin–ferrocene conjugates was prepared and investigated for *in vitro* anticancer activity against the MDA-MB-231 breast cancer line.^196^ They all showed moderate antiproliferative character. Compounds 125a and 125b (Fig. 124) showed maximum potency with IC_50_ values of 11.7 and 11.8 μM, respectively.

**Fig. 125:**
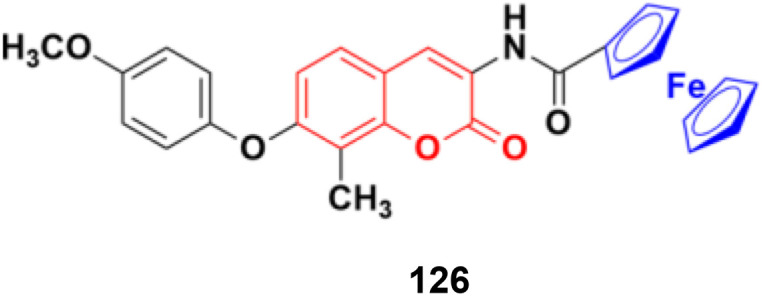
Chemical structure of coumarin–ferrocene hybrid 126.

The coumarin–ferrocene hybrid 126 (Fig. 125) showed good anticancer activity when examined against the HCC38 cancer cell line with IC_50_ = 1.06 μM.^197^

**Fig. 126:**
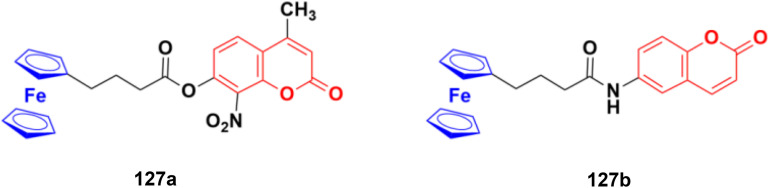
Chemical structures of coumarin–ferrocene hybrid 127a and b.

Seven novel coumarin–ferrocene conjugates were synthesized and their biological activities thoroughly investigated against several human cancer cell lines.^198^ Most of the hybrids showed moderate and good activity compared to the reference adriamycin. Compound 127a (Fig. 126) showed good potency against the BIU-87 and MCF-7 cancer cell lines with the IC_50_ values of 1.09 and 12.10 μM, respectively, while 127b was the most effective against SGC-7901 with IC_50_ = 3.56 μM.

#### 2.11.2. Miscellaneous coumarin-metal hybrids

**Fig. 127:**
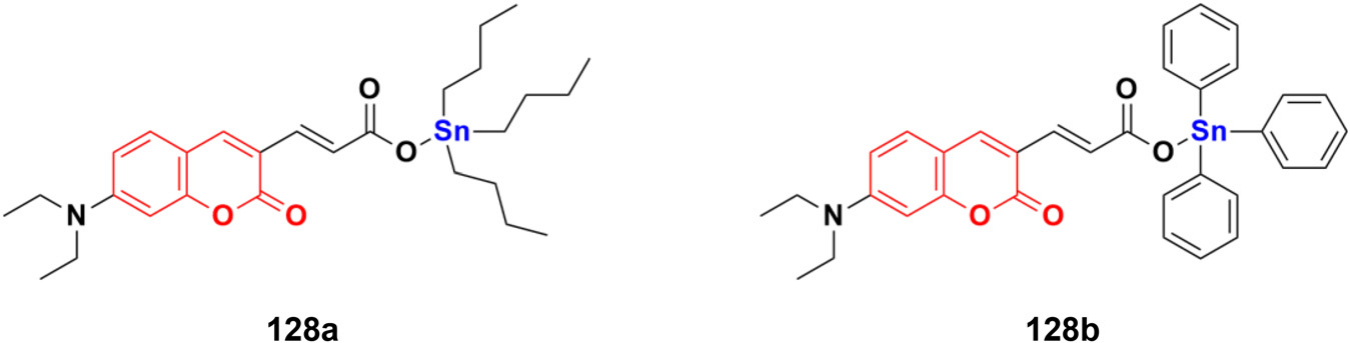
Chemical structures of coumarin–organotin hybrid 128a and b.

Two organotin(iv) carboxylate complexes containing a coumarin moiety were designed and their antitumor properties routinely investigated.^199^ The results indicated that complexes 128a and 128b (Fig. 127) could induce apoptotic cell death through mitochondrial dysfunction and ROS elevation pathways.

**Fig. 128:**
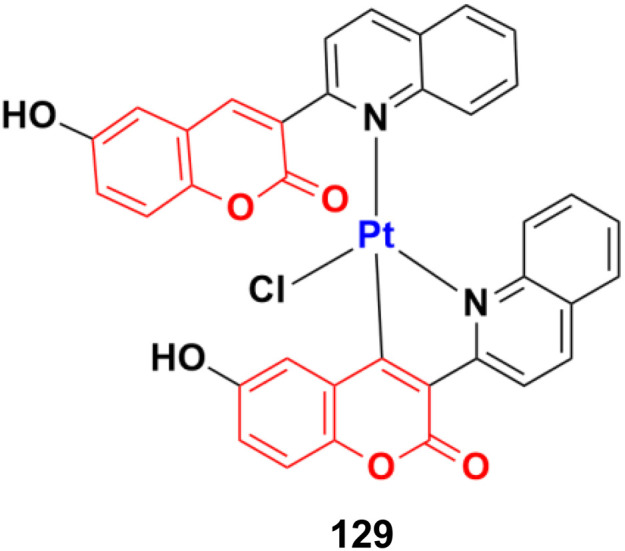
Chemical structure of organoplatinum(ii) complex with coumarin–quinoline moiety 129.

A series of novel organoplatinum(ii) complexes was designed and their cytotoxic effects on various cancer cell lines and drug-resistant cancer cell lines examined.^200^ Among them, complex 129 showed marked potency against the HeLa and A549/DDP cell lines with IC_50_ values of 0.15 ± 0.09 and 0.10 ± 0.05 μM, respectively (Fig. 128).

**Fig. 129:**
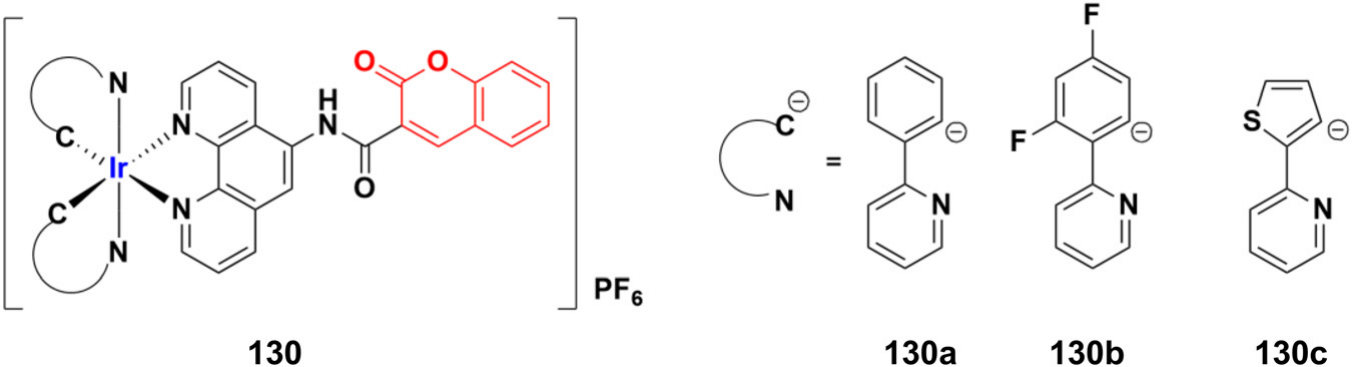
Chemical structures of iridium(iii) complex 130a–c.

Three coumarin-appended phosphorescent cyclometalated iridium(iii) complexes, 130a–c (Fig. 129), were explored as mitochondria-targeted theranostic anticancer agents.^201^ All three complexes could specifically target mitochondria and show better antiproliferative activities than cisplatin against various cancer cells including cisplatin-resistant cells. They could penetrate human cervical carcinoma (HeLa) cells quickly and efficiently, and carried out theranostic functions by simultaneously inducing and monitoring the morphological changes in the mitochondria. The mechanism studies showed that they exert their anticancer efficacy by initiating a cascade of events related to mitochondrial dysfunction. Genome-wide transcriptional and connectivity map analyses revealed that the cytotoxicity of complex 130c is associated with pathways involved in mitochondrial dysfunction and apoptosis.

**Fig. 130:**
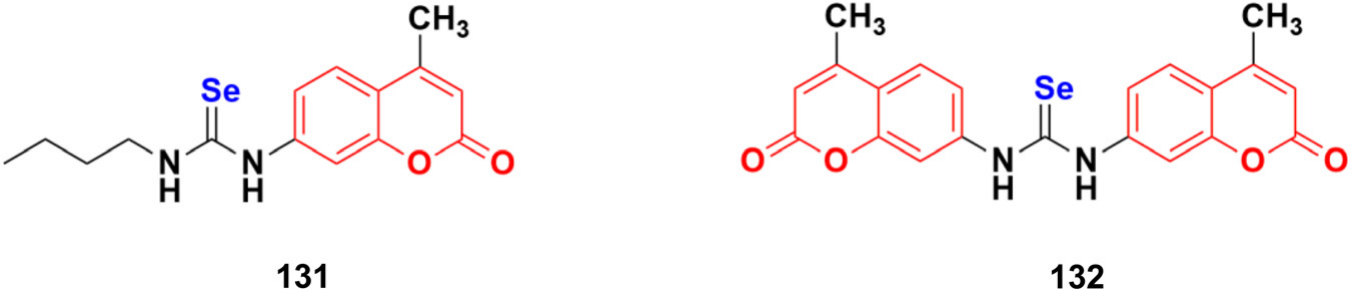
Chemical structures of selenocoumarin 131 and 132.

Three families, namely isoselenocyanate, selenocarbamates, and selenoureas, were designed and tagged with the coumarin moiety, and their antiproliferative properties were analyzed.^202^ Among them, selenourea 131 (Fig. 130) showed significant cytotoxic activities with GI_50_ values of 3.0 ± 0.5, 2.3 ± 0.7, 5.2 ± 0.4, 2.9 ± 1.1, and 3.8 ± 1.4 μM against the A549, HBL-100, HeLa, T-47D, WiDr cell lines, respectively. Moreover, dimer 132 also showed prominent cytotoxic activities with GI_50_ values of 3.2 ± 0.7, 3.5 ± 1.4, 2.9 ± 0.1, 3.6 ± 0.4, and 4.5 ± 1.0 μM against the above-mentioned cancer cell lines, respectively.

**Fig. 131:**
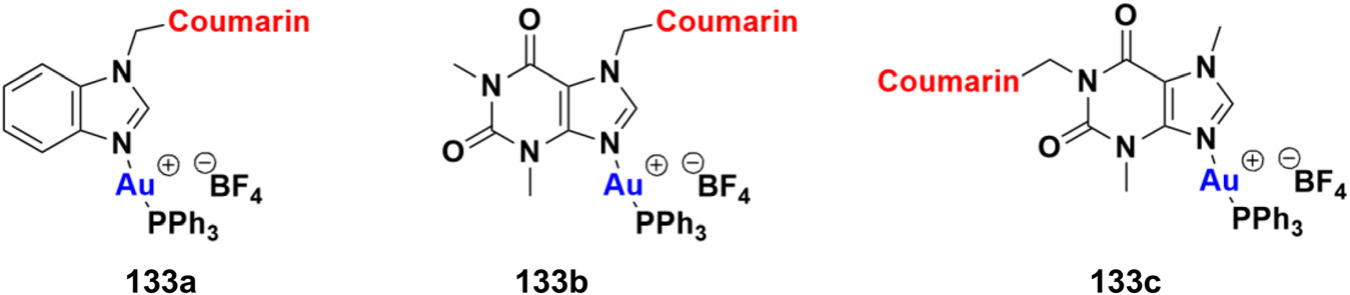
Chemical structures of gold(i)-coumarin 133a–c.

Three new gold(i)-coumarin-based trackable therapeutic complexes (133a–c) (Fig. 131) were synthesized and their antiproliferative properties on several types of cancer cell lines including colon, breast, and prostate investigated.^203^ They all displayed moderate anticancer activities against MDAMB-231, PC3, SW480, and HEK293T.

**Fig. 132:**
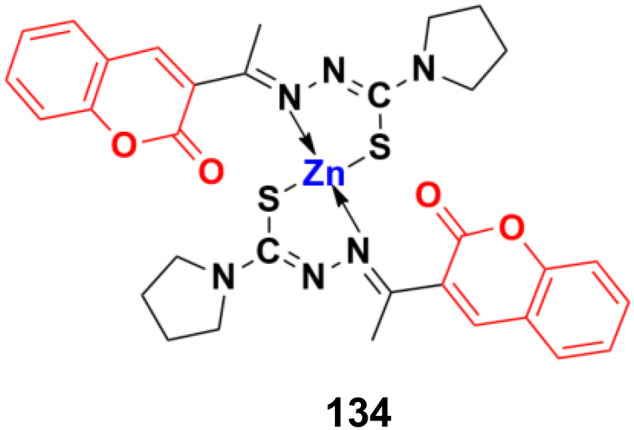
Chemical structure of zinc(ii)-coumarin complex 134.

The zinc(ii) complex of 3-acetylcoumarin thiosemicarbazone (134) (Fig. 132) showed significant cytotoxicity against human liver carcinoma (HepG-2) and lymphoblastoid multiple myeloma (IM-9) cell lines with the IC_50_ value of 25 μg mL^−1^.^204^ The spectroscopic results suggested that the complex interacted with CT-DNA through the intercalative binding mode.

**Fig. 133:**
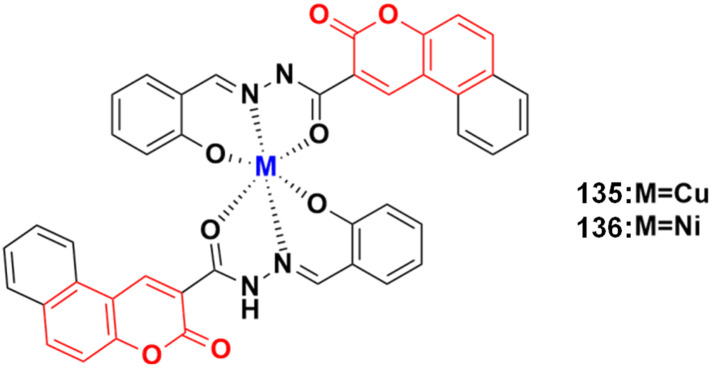
Chemical structure of copper(ii)-coumarin complex 135 and nickel(ii)-coumarin complex 136.

Two new copper(ii) (135) and nickel(ii) (136) (Fig. 133) complexes with a new coumarin derivative were synthesized and their cytotoxic activities determined by the MTT assay.^205^ The results showed that the designed drugs have significant cytotoxic activity against the HepG2, HL60, and PC3 cell lines. Cell apoptosis was detected by annexin V/PI flow cytometry and the results showed that the two complexes can induce apoptosis of the three human tumor cells.

**Fig. 134:**
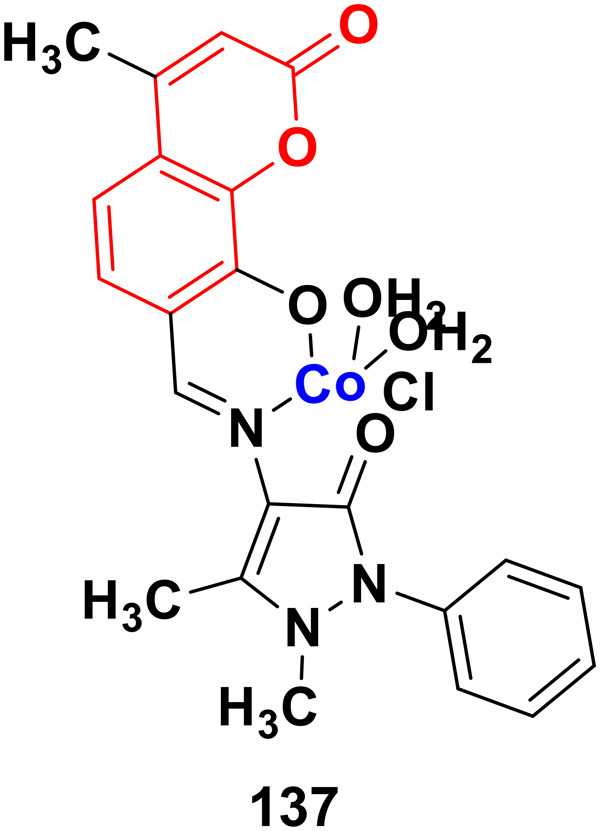
Chemical structure of cobalt(ii)-coumarin complex 137.

A Co(ii) complex of coumarin [Co(CUAP)(H_2_O)_2_Cl] (137) (Fig. 134) exhibited significant antiproliferative properties against the MCF-7 and K-562 cancer cell lines with IC_50_ values of less than 10 μg mL^−1^.^206^

**Fig. 135:**
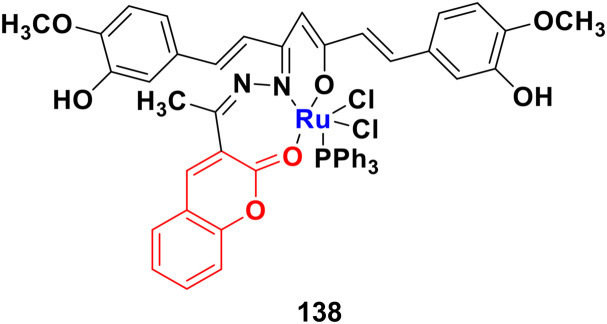
Chemical structure of Ru(iii)-coumarin complex 138.

A Ru(iii) complex synthesized from a coumarin derivative (138) (Fig. 135) was observed to be potent against the MCF-7 cancer cell line with an IC_50_ value of less than 10 μg mL^−1^.^207^

Besides these findings, various metal-based coumarin complexes show significant cytotoxic activities against various cancer cell lines and this strategy is becoming increasingly important, which has a bright prospect.^208^

### 2.12. Miscellaneous coumarin hybrids

**Fig. 136:**
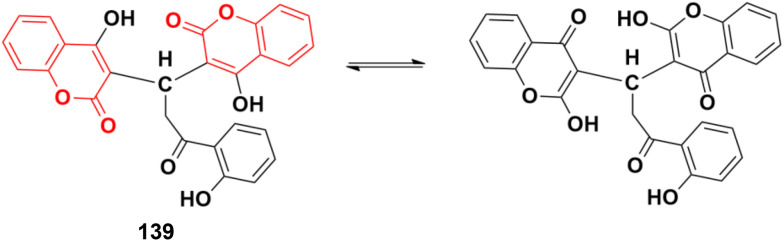
Chemical structure of coumarin dimer 139.

The coumarin derivative bis(4-hydroxy-2*H*-chromen-2-one)coumarin 139 (Fig. 136) possessed marked antitumor activity against the MCF-7 cancer cell line by inducing cell cycle arrest in the G2/M phase.^209^

**Fig. 137:**
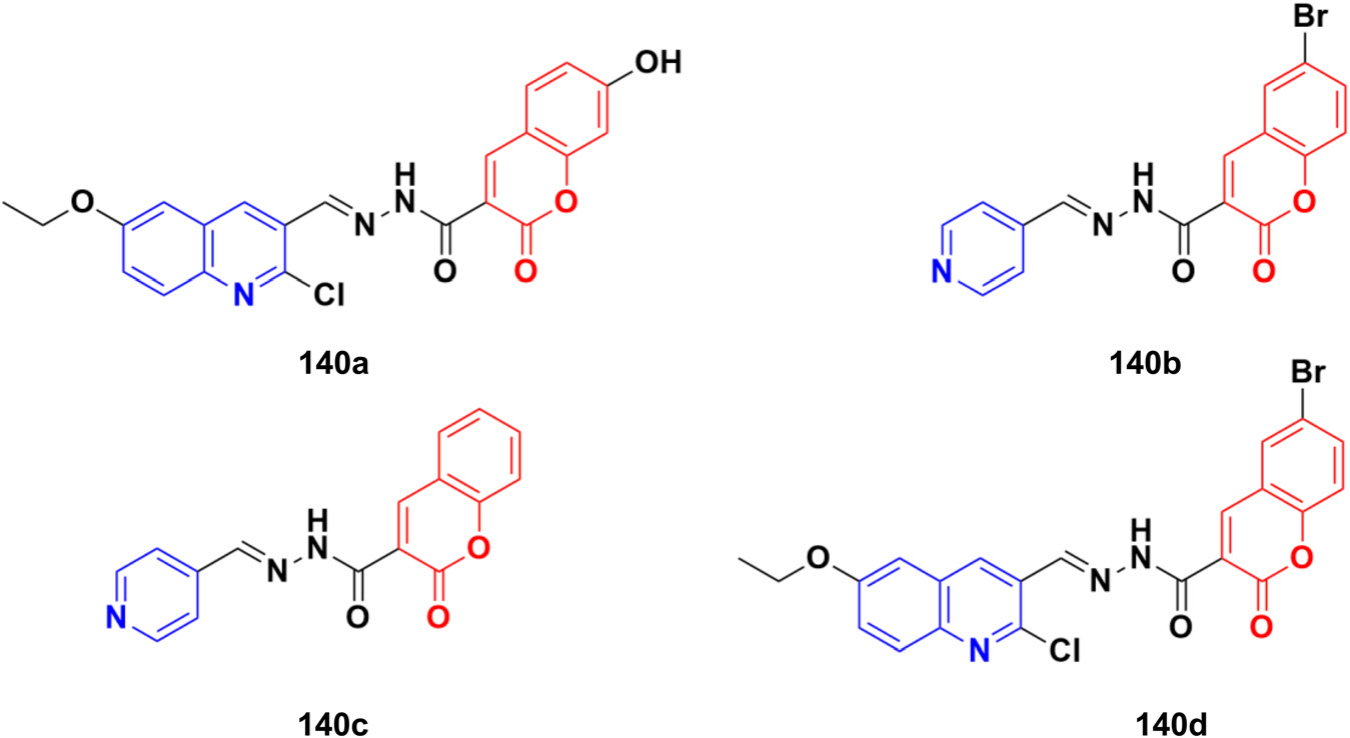
Chemical structures of coumarin derivative 140a–d.

A series of hydrazide–hydrazone and amide-substituted coumarin derivatives was synthesized and evaluated *in vitro* for their antitumor activity.^210^ Among them, compound 140a (Fig. 137) showed the maximum potency against the Panc-1 cell line with IC_50_ = 0.129 ± 0.019 μM and selectivity ratio of >387.60. Compound 140b possessed significant effectivity against the HepG2 cell line with IC_50_ = 4.892 ± 0.086 μM and selectivity ratio of >10.22. Compound 140c showed marked potency against CCRF cells with IC_50_ = 3.108 ± 0.439 μM and selectivity ratio of >16.09. Besides, compound 140d was effective against all the cell lines with IC_50_ values of 5.449 ± 1.380, 9.417 ± 0.548, and 7.448 ± 4.579 against Panc-1, HepG2, and CCRF cells with the corresponding selective ratio of >9.18, >5.31, and >6.71, respectively.

**Fig. 138:**
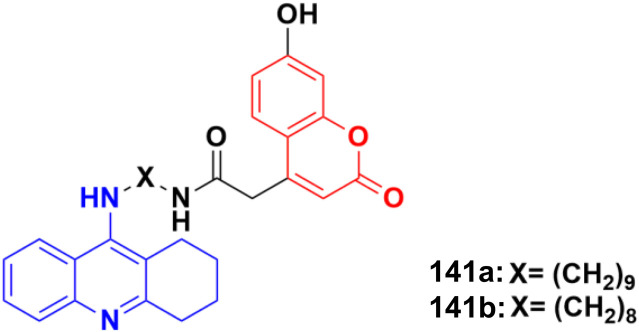
Chemical structures of coumarin-tacrine hybrid 141a and b.

Tacrine-coumarin hybrids 141a and 141b (Fig. 138) showed significant anti-metabolic activity against the 4T1 cell line, with IC_50_ values of 5.7 μM and 7.0 μM, respectively, while compound 141a also showed promising activity against the MCF-7 cell line, with an IC_50_ value of 6.0 μM.^211^

**Fig. 139:**
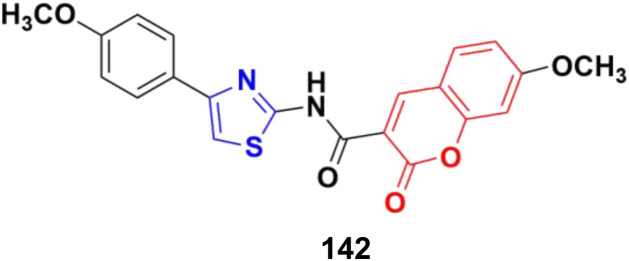
Chemical structure of coumarin–thiazole hybrid 142.

A series of quinoline and thiazole-containing coumarin analogs was developed and their cytotoxic ability examined on mouse leukemic cells.^212^ The result indicated that 142 (Fig. 139) showed potent activity against EAC and DLA cells in the MTT assay (15.3 μM), trypan blue (15.6 μM), and LDH (14.2 μM) leak assay with 5-fluorouracil as the standard. The experimental data showed that compound 142 induced apoptotic cell death by activating apoptotic factors such as caspase-8 &-3, CAD, cleaved PARP, γ-H2AX, and by degrading genomic DNA of cancer cells, and thereby decreasing the ascitic tumor development in mice. The molecular docking study revealed that compound 142 has a very good interaction with caspase 3 protein by binding with the Arg 207 amino acid through a hydrogen bond.

**Fig. 140:**
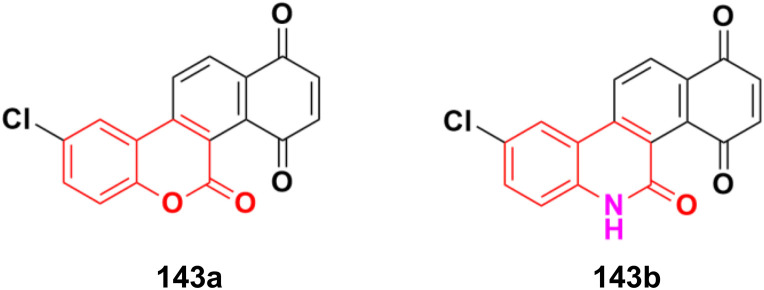
Chemical structures of coumarin derivative 143a and b.

An interesting fact can be nicely shown from the work by Zwergel *et al.*^213^ The antiproliferative property of a particular coumarin-based hybrid can be significantly enhanced by slight modification in the coumarin moiety (Fig. 140, Table 1).

**Table 1:** Biochemical data for compound 143a and b

Compound	Inhibition data (IC_50_, μM)		Viability (MTT assay, IC_50_, μM)					
	CDC25A	CDC25C	A549	MCF7	PC3	U373n	Hs683	SKMEL28
143a	2.57 ± 0.2	1.44 ± 0.2	25	13	19	12	6	33
143b	4.33 ± 0.4	5.72 ± 0.5	0.3	1.2	0.3	0.1	0.2	0.3

**Fig. 141:**
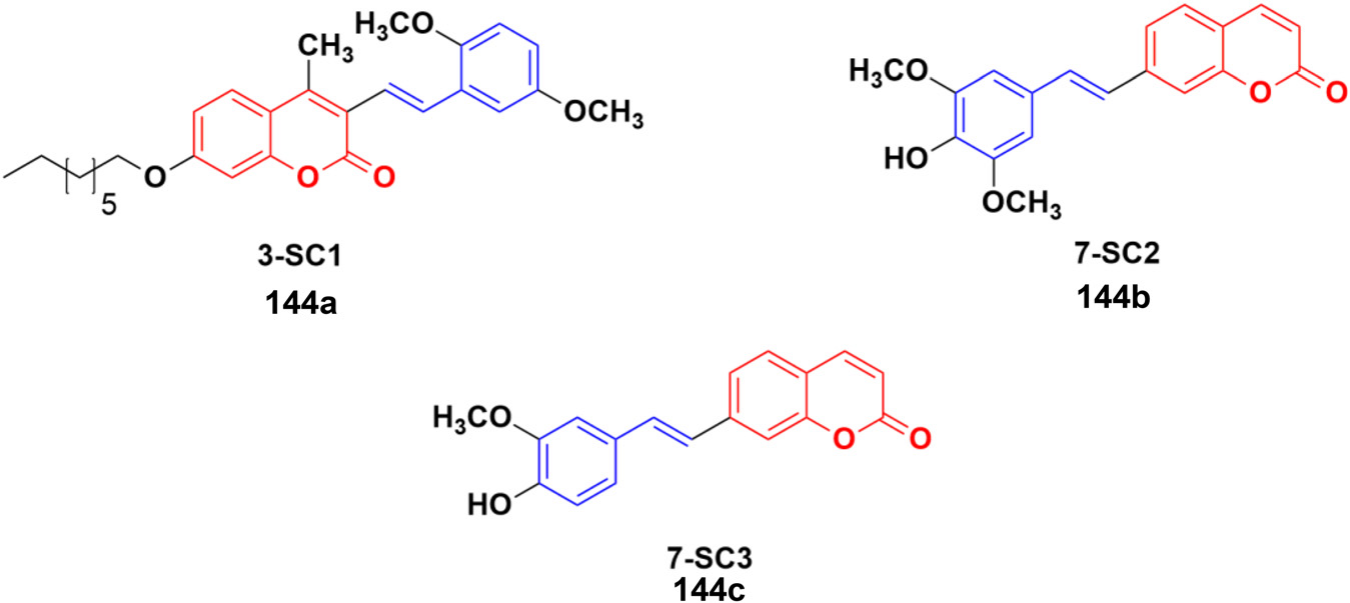
Chemical structures of styryl coumarin 144a–c.

The styryl coumarin hybrids 3-SC1 (144a), 7-SC2 (144b), and 7-SC3 (144c) (Fig. 141) decreased the cell viability of SW480 in a time- and concentration-dependent manner (IC_50_-SW480/48 *h* = 6.92; 1.01 and 5.33 μM, respectively) with high selectivity indices after 48 h of treatment (>400; 67.8 and 7.2, respectively).^214^ Among them, the most active molecule 7-SC2 induced a greater production of ROS in comparison with the control (*p* < 0.05) together with a significant increase in the expression of p53 and caspase-3, and a significant reduction in the production of interleukin-6 of SW480 cells. When colon carcinogenesis was induced in Balb/c mice by intraperitoneal injection of azoxymethane, a significant reduction (*p* < 0.05) in the number of preneoplastic lesions of the mice treated with styryl coumarin hybrid 7-SC2 was observed in the control group. Moreover, no side effects were associated with the administration of the compound. All these *in vitro* results and the effective reduction of preneoplastic lesions *in vivo* suggest that styryl coumarin 7-SC2 induces apoptosis in primary tumor cells and implies its potential ability at the early post-initiation phases of colon carcinogenesis.

**Fig. 142:**
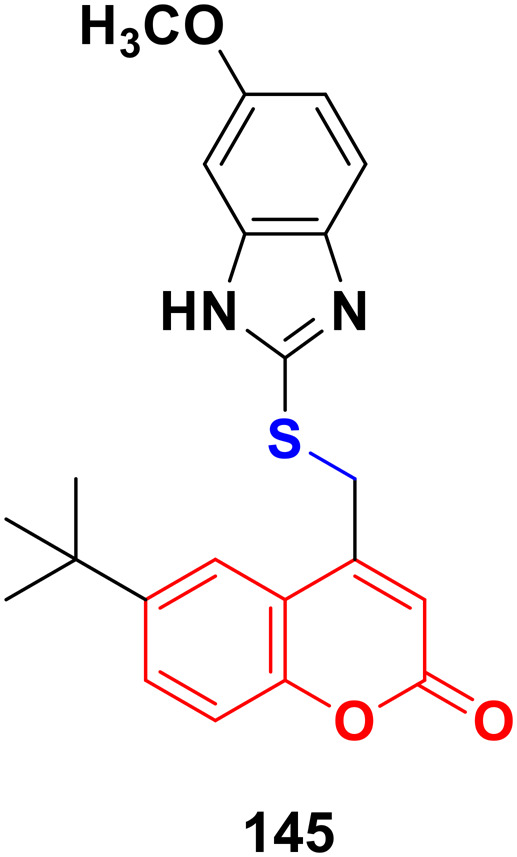
Chemical structure of methylene thio-linked coumarin derivative 145.

A series of methylene thio-linked coumarin derivatives was prepared by the reaction of substituted 4-(bromomethyl)-2*H*-chromen-2-one with various heterocyclic mercapto compounds *via* S_N_2 reactions in the presence of K_2_CO_3_ as a catalyst and their *in vitro* anticancer activity screened against the MCF-7 cancer cell line.^215^ Compound 145 (Fig. 142) with a methoxy-substituted benzimidazole ring was found to be the most effective with an IC_50_ value of 0.18 μM.

**Fig. 143:**
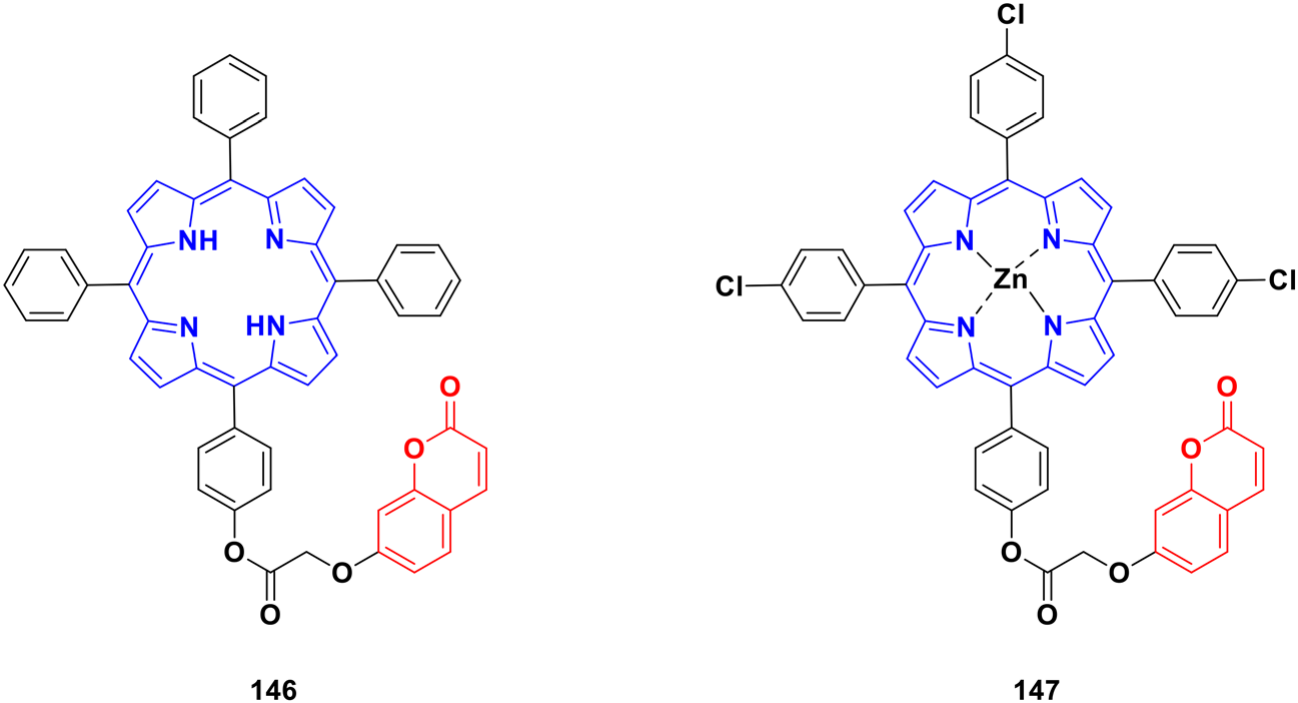
Chemical structures of coumarin–porphyrin complex 146 and zinc-coumarin–porphyrin complex 147.

Twenty porphyrin–coumarin compounds were synthesized *via* the condensation reaction of porphyrins with coumarin derivatives and their cytotoxic activity evaluated against A549 and HepG2 cells under light irradiation.^216^ The structure–activity relationship studies indicated that the coumarin derivatives with shorter alkyl chains to porphyrins exhibited both photodynamic therapy (PDT) and chemotherapy. Moreover, the insertion of metal Zn in the porphyrins also increased the PDT effect of the compounds. Compound 146 (Fig. 143) was found to be the most effective against the HepG2 cell line with an IC_50_ value of 67.66 ± 0.61 μmol L^−1^. Alternatively, the Zn-containing compound 147 exhibited the maximum potency against the A549 cell line with an IC_50_ value of 52.37 ± 1.17 μmol L^−1^ (both under light irradiation).

Besides the above-mentioned coumarin hybrids, coumarin-hydroxamic acid,^217^ coumarin–ergosterol peroxide,^218^ coumarin–formonontin,^219^ coumarin–costunolide,^220^ coumarin–curcuminoid,^221^ coumarin–furan,^222^ coumarin–maltol,^223^ coumarin–carbazole,^224^ coumarin–quinazoline,^225^ coumarin-steroidal,^226,227^ coumarin-sugar,^228–232^ coumarin-thiazin-2-thione,^233^ coumarin–thiophosphate,^234^ coumarin-triazolothiadiazines,^235^*N*-heterocyclocoumarin,^236^ hydroxymercapto-methylcoumarin,^237^ triazole-tethered coumarin–isatin,^238^ and others^239–241^ also showed certain anticancer activities.

## 3. Patents

Coumarin hybrids have been successfully used as potential anticancer agents and many international patents have been filed in this regard.

**Fig. 144:**
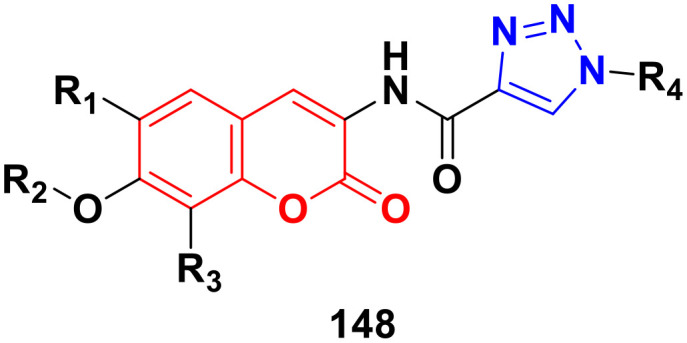
General chemical structure of triazole-modified coumarin-based compound 148.

In 2015, some triazole-modified coumarin-based compounds (148) were patented, which were proposed to be used in the treatment of various cancers such as breast and prostate cancer.^242^ The general structure of the compounds is shown in Fig. 144.

**Fig. 145:**
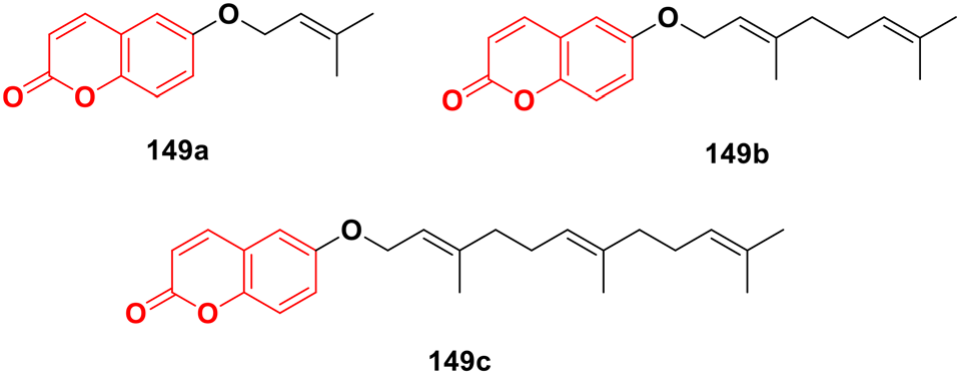
Chemical structures of 6-substituted coumarins 149a–c.

In 2016, various coumarin derivatives 149a–c with substituents at the 6-position with five or more than five carbon atoms were patented, which were used to treat pancreatic cancer.^243^ The structures of some of the compounds are shown in Fig. 145.

**Fig. 146:**
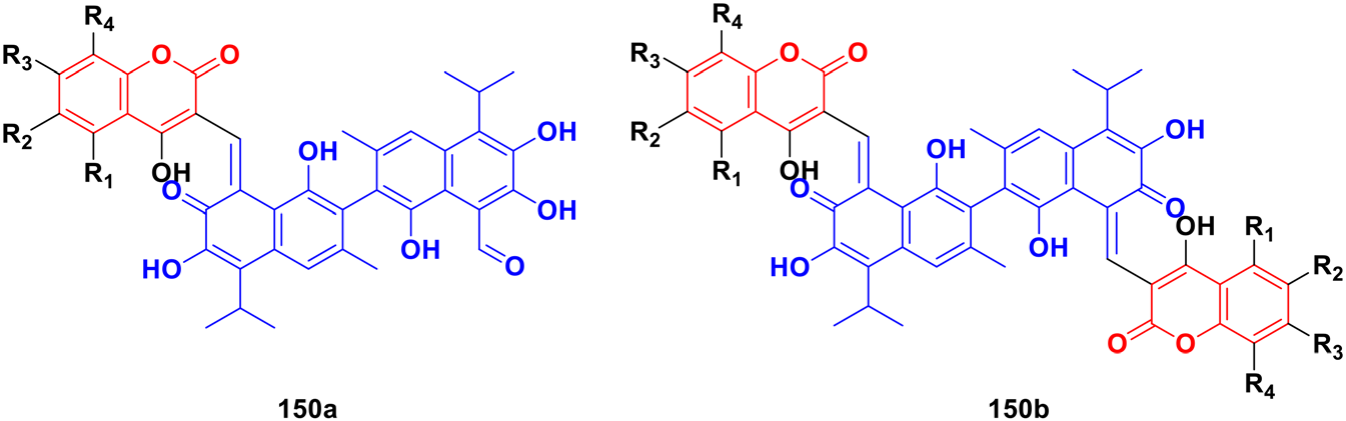
Chemical structures of coumarin–gossypol hybrid 150a and b.

In 2017, the design of different coumarin–gossypol hybrids with antitumor activities was patented,^244^ which had two different general structures (150a and 150b), as shown in Fig. 146.

**Fig. 147:**
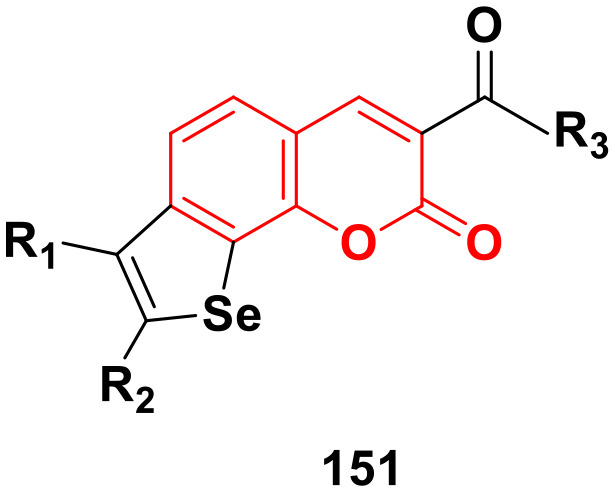
Chemical structure of selenium-containing coumarin derivative 151.

In 2018, the method for the synthesis of a library of selenium-containing coumarin derivatives (151) was patented.^245^ The general structure of the compounds is presented in Fig. 147. These compounds were proposed to prevent primary cancer.

**Fig. 148:**
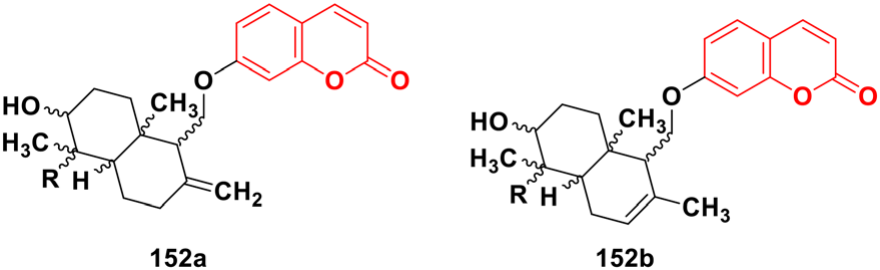
Chemical structure of terpenic coumarin derivative 152a and b.

In 2019, a patent was filed for the use of terpenic coumarin derivatives as potential anticancer agents with high selectivity and low side effects.^246^ The general structures of two types of these derivatives are shown in Fig. 148.

**Fig. 149:**
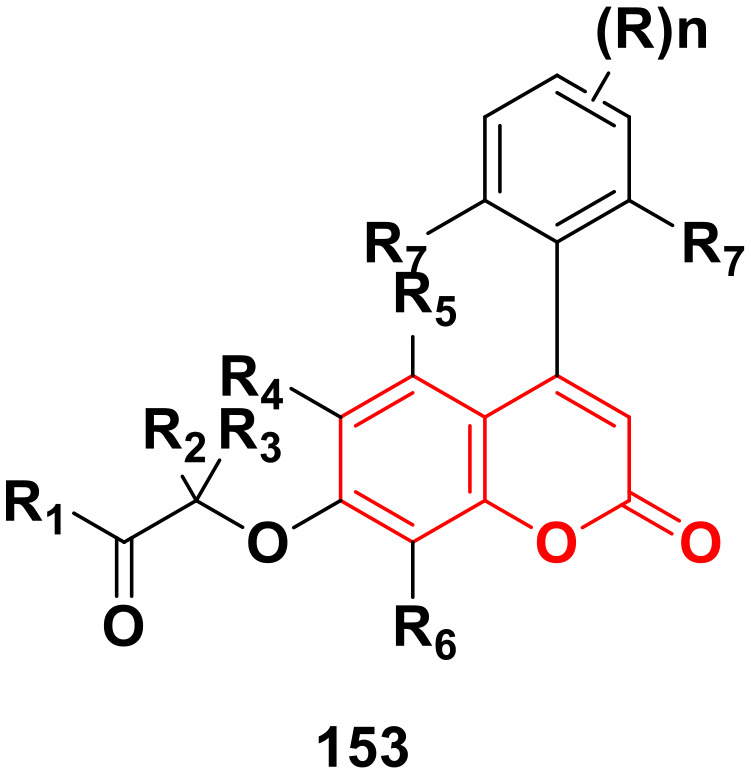
General chemical structure of 4-phenylcoumarin derivative 153.

In 2020, a patent was filed for several novel 4-phenyl-coumarin derivatives.^247^ The compounds were prepared and used as specific mitochondrial RNA polymerase inhibitors for cancer treatment. The general structure of compound 153 is presented in Fig. 149.

**Fig. 150:**
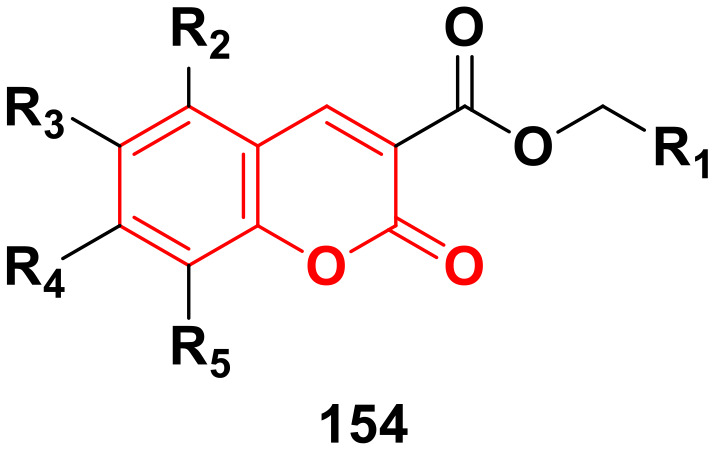
General chemical structure of coumarin derivative 154.

In 2022, the preparation of some coumarin derivatives (154) used for their anticancer and antioxidant properties was patented.^248^ The general structure of the compounds is shown in Fig. 150.

## 4. Conclusion

Presently, the high mortality rate of cancer represents a great concern in society. In this case, the coumarin scaffold has become a privileged molecule in the design of anticancer drugs in recent years. Besides, the strategy of its hybridization with other pharmacophores has also become widely accepted in the case of enhanced efficacy. However, the preclinical trials, *i.e.*, the study of *in vivo*, toxicity, specificity, and interaction of drugs is time-consuming and not cost-effective for researchers of non-profit organizations. Several coumarin derivatives with high anticancer potency were highlighted in this review but the discovery of novel therapeutic drugs is still pending. Moreover, the redox features of the dihydroxy group attached to simple coumarin may not be favorable *in vivo* and lead to side effects. Further, modification of the pyrone rings is needed to overcome these side effects.

The above-mentioned studies also revealed that coumarin azole derivatives bind directly to DNA, which can lead to further improvement in oxidative stress. Coumarin-metal hybrids will also flourish to synthesize new drugs.

We hope, in the future, that the problems of side effects and toxicity regarding the coumarin scaffold will be overcome and coumarin-based anticancer drugs will be available in the market to fight against cancer and help cost-effectively eradicate cancer.

## Conflicts of interest

There is no conflict of interest to declare.

## Supplementary Material
